# Examination of aminophenol-containing compounds designed as antiproliferative agents and potential atypical retinoids

**DOI:** 10.1016/j.bmc.2023.117214

**Published:** 2023-02-24

**Authors:** Ramesh M. Chingle, Masahiko Imai, Sarah Altman, Daisuke Saito, Noriko Takahashi, Terrence R. Burke

**Affiliations:** a Chemical Biology Laboratory, Center for Cancer Research, National Cancer Institute, National Institutes of Health, Frederick, MD 21702, USA; b Laboratory of Physiological Chemistry, Institute of Medicinal Chemistry, Hoshi University, 2-4-41 Ebara, Shinagawa, Tokyo 142-8501, Japan

**Keywords:** Retinoic acid, Fenretinide, *p*-Dodecylaminophenol, Retinoic acid receptors, Atypical retinoid

## Abstract

Retinoic acid (RA, **1**), an oxidized form of vitamin A, binds to retinoic acid receptors (RAR) and retinoid X receptors (RXR) to regulate gene expression and has important functions such as cell proliferation and differentiation. Synthetic ligands regarding RAR and RXR have been devised for the treatment of various diseases, particularly promyelocytic leukemia, but their side effects have led to the development of new, less toxic therapeutic agents. Fenretinide (4-HPR, **2**), an aminophenol derivative of RA, exhibits potent antiproliferative activity without binding to RAR/RXR, but its clinical trial was discontinued due to side effects of impaired dark adaptation. Assuming that the cyclohexene ring of 4-HPR is the cause of the side effects, methylaminophenol was discovered through structure–activity relationship research, and *p*-dodecylaminophenol (*p*-DDAP, **3**), which has no side effects or toxicity and is effective against a wide range of cancers, was developed. Therefore, we thought that introducing the motif carboxylic acid found in retinoids, could potentially enhance the anti-proliferative effects. Introducing chain terminal carboxylic functionality into potent *p*-alkylaminophenols significantly attenuated antiproliferative potencies, while a similar structural modification of weakly potent *p*-acylaminophenols enhanced growth inhibitory potencies. However, conversion of the carboxylic acid moieties to their methyl esters completely abolished the cell growth inhibitory effects of both series. Insertion of a carboxylic acid moiety, which is important for binding to RA receptors, abolishes the action of *p*-alkylaminophenols, but enhances the action of *p*-acylaminophenols. This suggests that the amido functionality may be important for the growth inhibitory effects of the carboxylic acids.

## 1. Introduction

### 1.1. Background

**Fig. 1.**
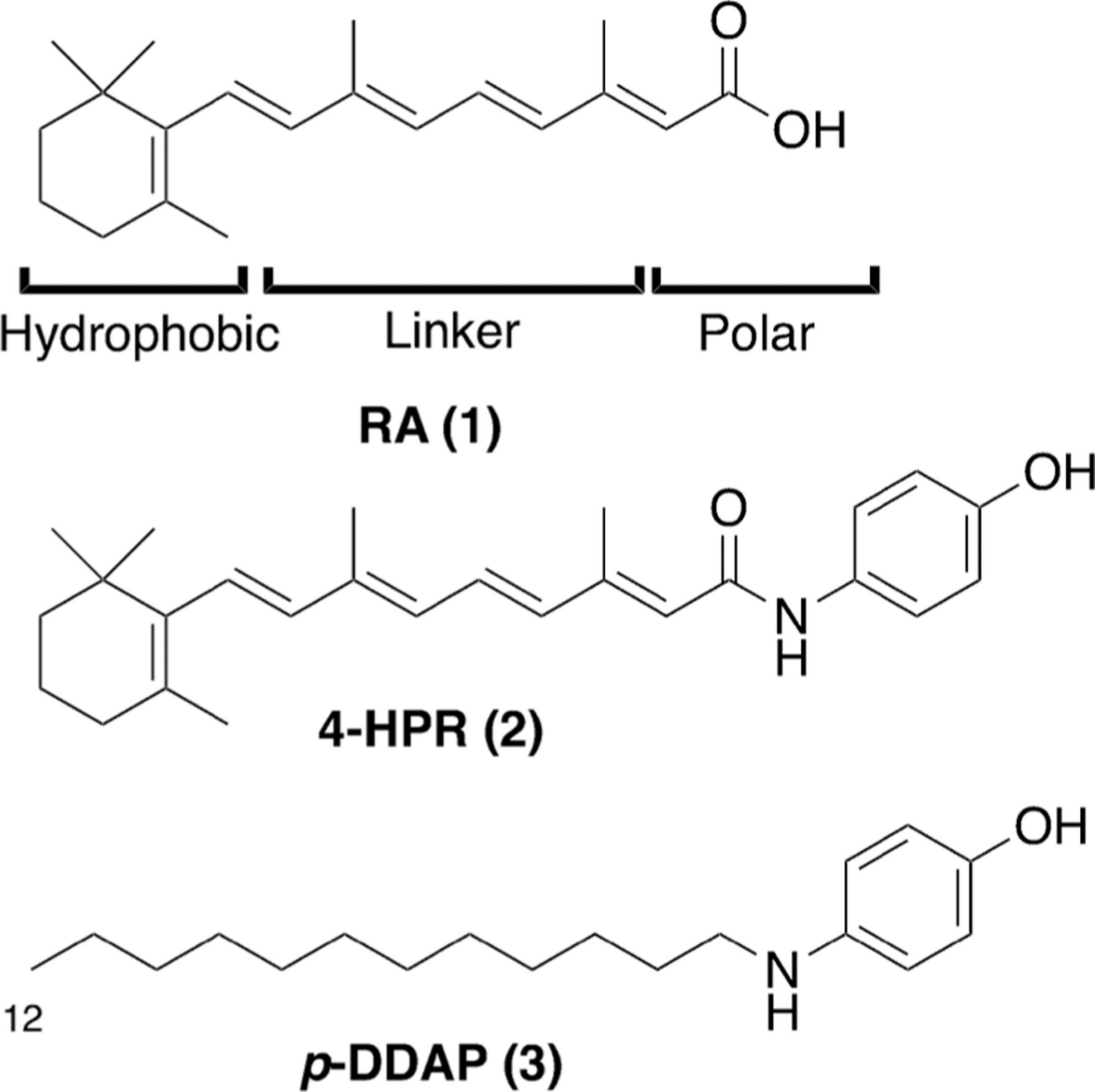
Structures of parent compounds **1**–**3**.

Retinol, also known as vitamin A, is a polyisoprenoid natural product that plays important roles in a variety of physiological processes. Oxidative transformation of its chain-terminating primary hydroxyl group yields the corresponding aldehyde retinal, which serves a central role in vision. *All-trans* retinoic acid (RA, **1**, Fig. 1) is an oxidized form of retinol that binds to retinoic acid receptors (RARs), resulting in modulation of gene expression related to vital physiological processes, including cell growth, differentiation, survival, and death. In contrast, the isomeric RA variant, *9-cis* retinoic acid binds to retinoid X receptors (RXRs), which can exist as heterodimers with RARs or other nuclear receptors.^1^ Retinoids (synthetic ligands that engage RARs) and rexinoids (RXR-binding compounds) have therapeutic value against a variety of metabolic diseases and cancer, particularly for the treatment of promyelocytic leukemia. Because retinoids, and to a lesser extent rexinoids, exert unwanted side-effects, there is a need to develop new therapeutic agents that are less toxic. “Atypical retinoids” are synthetic analogs that bind and transactivate RARs and have therapeutic promise as anticancer drugs.^2^

Fenretinide, [*N*-(4-hydroxyphenyl)retinamide, 4-HPR, **2**, Fig. 1], is an atypical retinoid that has chemo-preventative and anti-proliferative properties, but these are accompanied by side effects that include night blindness and ocular toxicity. Despite its structural similarity to RA, 4-HPR does not bind to RARs and it is a poor inducer of differentiation. Its antiproliferative activity is thought to be related to its ability to induce apoptosis by a mechanism that is RAR-independent and that might be related to the generation of reactive oxygen species (ROS).^3^ This is somewhat paradoxical, since 4-HPR functions as an antioxidant and accordingly, it might participate in redox cycling reactions.^4^ RA and retinoids are typically composed of three structural components; a hydrophobic region that is often composed of ring structures; a linker region and polar functionality at the terminus of the linker segment (Fig. 1).^2,5^ While sharing with RA the first two of these structural elements, 4-HPR includes a neutral 4-hydroxyamido group at the terminus of the linker, rather than the characteristic polar functionality. We have previously reported antiproliferative compounds that contain the 4-aminophenol moiety found in 4-HPR as well as long-chain *N*-alkyl groups that are somewhat reminiscent of the extended polyene chains found in 4-HPR and other retinoids.^6^ We found that antiproliferative potencies of the alkylaminophenols are related to alkyl chain lengths. Optimum antiproliferative potencies were obtained with the 12-methylene-long chain-containing *p*-dodecylaminophenol (*p*-DDAP, **3**, Fig. 1), which may be viewed as structurally simplified variant of 4-HPR.^7^ We have shown that *p*-DDAP is more potent than RA in suppressing the growth of breast,^8^ prostate,^9^ promyelocytic leukemia,^7^ neuroblastoma,^10–11^ pancreatic,^12^ and cholangiocarcinoma^12^ cells in culture. Furthermore, we have found that *p*-DDAP has antitumor effects on prostate cancer xenograft-bearing mice, while not affecting blood retinol concentrations, which is an undesirable side effect of treatment with 4-HPR and which can lead to reduced night vision.^9^ We wondered whether it might be possible to enhance antiproliferative potency in compounds structurally related to *p*-DDAP by introducing motifs found in retinoids and rexinoids.

### 1.2. Analog design

**Fig. 2.**
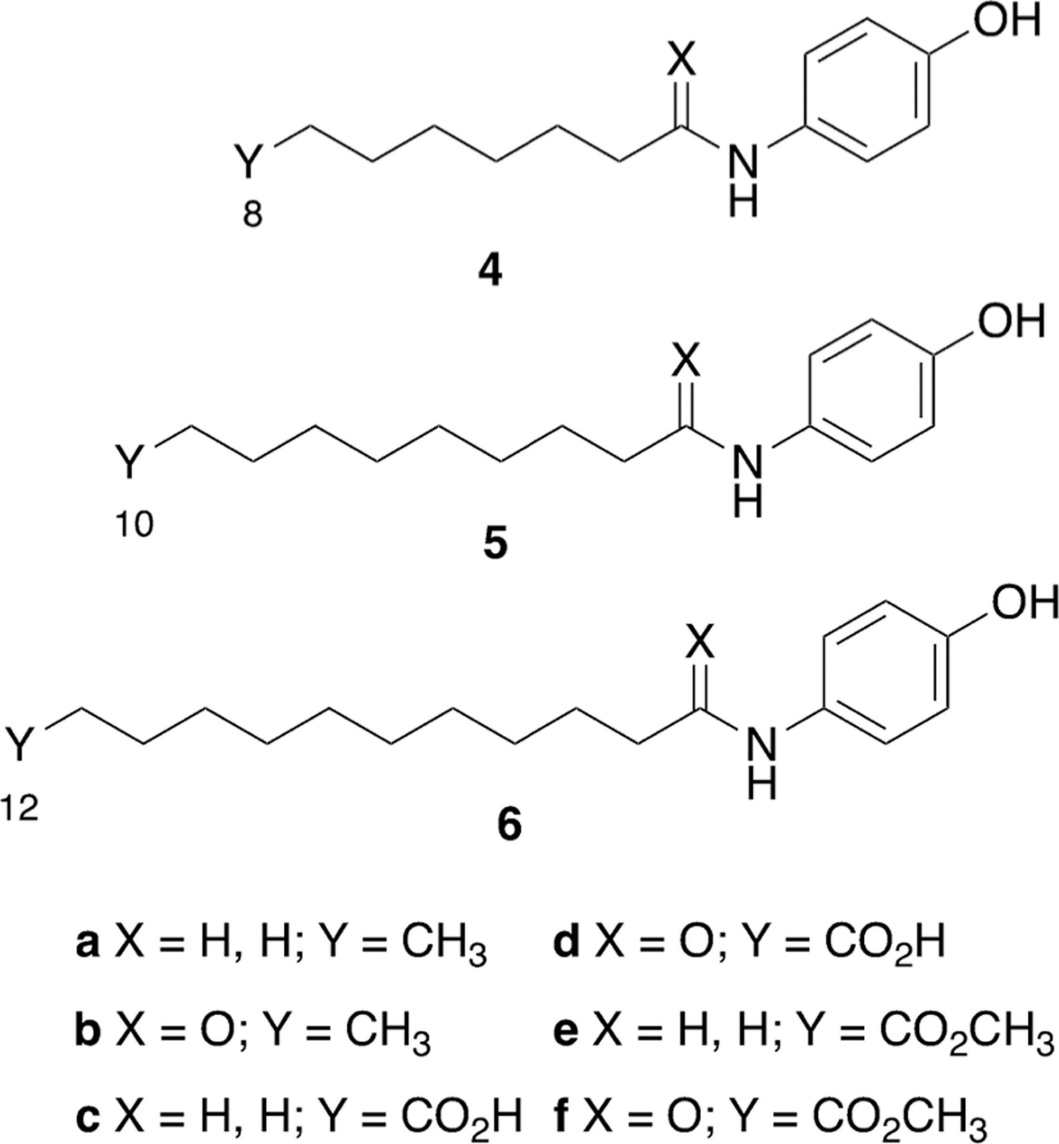
Structures of analogs examined in the current study (**4a**-**f** to **6a**-**f**). [Note: **6a** = **3**]

Structure-activity relationship studies conducted on 4-HPR have revealed that the 4-aminophenol moiety is key to its ability to suppresses the growth of cancer cells.^13^ Among a series of 4-alkylaminophenols (**4a** – **6a**), antiproliferative potencies have been shown to be enhanced by longer chain lengths. Increasing alkylaminophenol chain length also increases superoxide trapping properties and reduces lipid peroxidation.^7^ However, we found that conversion of the amines to their corresponding amides (**4b** – **6b**), significantly decreases growth inhibitory potencies and ability to function as lipid peroxidation inhibitors (Fig. 2).^7,11^ In our current study we present the design, synthesis and biological evaluation of a series of analogs that incorporate elements of 4-HPR or RA into our alkylaminophenol platforms that explore this possibility. We designed analogs focused on the incorporation of terminal carboxylic acid functionality that would introduce key chain-terminal polar groups present in parent RA. We prepared carboxylic acid-containing variants of the aminoalkyls (**4a** – **6a**) to yield the corresponding acids (**4c** – **6c**) and the amidoalkyls (**4b** – **6b**) to yield the corresponding acids (**4d** – **6d**). Finally, we prepared the methyl esters (**4e** – **6e**, **4f** – **6f**) of all acids to potentially facilitate cellular uptake (Fig. 2).

## 2. Materials and methods

### 2.1. Chemicals

RA, ethylenediaminetetraacetic acid (EDTA) and dimethyl sulfoxide (DMSO) were obtained from Sigma Chemical (St. Louis, MO, USA). All other chemicals were of reagent grade.

### 2.2. Cell lines and culture conditions

Human breast cancer cell line MCF-7 and human prostate cancer cell line PC-3 were obtained from the RIKEN Cell Bank (Tsukuba, Ibaraki, Japan). MCF-7 and PC-3 cells were maintained in RPMI 1640 Medium (GIBCO, Grand Island, NY, USA) supplemented with 10% fetal bovine serum (FBS, GIBCO) and 1 mM 4-(2-hydroxyethyl)-1-piperazineethanesulfonic acid (HEPES, GIBCO). All cells described above were incubated at 37 °C in a humidified atmosphere of 5% CO_2_ in air. Cell number was estimated using an electric particle counter (Coulter Electronics, Hialeah, FL, USA).

### 2.3. Cell culture

Cells (1 ~ 4 × 10^4^ cells/mL) were incubated at 37 °C in a humidified atmosphere of 5% CO_2_ in air. After 24 h, various concentrations of compounds were added to the cultures, and then were incubated for 72 h. Control cells were treated with 0.1% DMSO.

### 2.4. Cell growth

Viable cell number was estimated using 3-(4,5-dimethylthiazol-2-yl)-2,5-diphenyltetrazolium bromide (MTT) as described previously.^14^ Values for percent net cell growth were calculated with the following formula: [(absorbance of experimental cell concentration) – (absorbance of initial cell concentration)/(absorbance of control cell concentration) – (absorbance of initial cell concentration)] × 100.

### 2.5. In vitro RARα and RXRα reporter gene assays

Compound **6d** was investigated with commercially available assays for human RARα (product number: IB02201–32) and RXRα (product number: IB00801–32; INDIGO Biosciences, Inc., State College, PA, USA; Purchased from Cosmo Bio Co., LTD, Tokyo, Japan). RAR and RXR assay kits included RARα and RXRα reporter cells, cell recovery medium, compound screening medium, positive control (10 mM 9-*cis*-retinoic acid in DMSO, reference agonist for RARα or RXRα), detection substrate, detection buffer, snap-in 8-well strips, and plate frame were conducted as described the manufacturer-supplied protocols.

Briefly, for RARα and RXRα assays, cells were seeded into well strips (100 μL/well) and then culture media with test compound was added (100 μL/well) and incubated at 37 °C for 24 h. The media was removed, luciferase detection reagent (detection substrate dissolved in detection buffer) was added (100 μL/well), and light emission was quantified using a 2030 multilabel reader ARVO^™^ X2 (PerkinElmer, Waltham County, MA, USA). Each assay strip included three replicate wells per vehicle control. Compound was initially tested across a range of doses including 0.5, 1, 4, 10, and 20 μM on the active dose response range. Positive control (9-*cis*-retinoic acid) was run across a range of doses as specified by the manufacturer protocols (0.5, 5, 50, and 500 nM).

### 2.6. Flow cytometry

MCF-7 and PC-3 cells (2 × 10^4^ cells/mL) were incubated at 37 °C in a humidified atmosphere of 5% CO_2_ in air. After 24 h, cells were treated with 10 μM **6d** or DMSO for 24 h. Cells were harvested and fixed in 70% ethanol at 4 °C overnight. Before the analysis, cells were washed with phosphate buffered saline (PBS) twice, treated with 100 μg/mL RNase A at 37 °C for 30 min, and then stained with 10 μg/mL propidium iodide (PI). Cell cycle analysis was performed using a FACSVerse flow cytometer (Becton Dickinson, San Jose, CA, USA).

### 2.7. Immunoblotting analysis

Whole cell lysates were prepared with NP40 lysis buffer [25 mM Tris-HCl (pH 8), 150 mM NaCl, 0.5% NP40, 4 mM NaF, 0.1 mM Na_3_VO_4_, and protease inhibitor cocktail].^15^ The protein concentration of the whole cell lysates was determined by the Bicinchoninic Acid (BCA) Protein Assay (Pierce, Rockford, IL, USA). Whole lysates were subjected to SDS-polyacrylamide gel electrophoresis (SDS-PAGE), and were transferred to Immobilon-P membranes (Millipore, Billerica, MA, USA). The membranes were probed with polyclonal antibodies against Bcl-2, Bcl-xL, ERK, phospho-ERK (p-ERK), Akt, phospho-Akt (p-Akt), LC3 (Cell Signaling, Danvers, MA, USA), and β-actin (Santa Cruz Biotechnology, Santa Cruz, CA, USA). Proteins were visualized using ECL-plus (Invitrogen).

### 2.8. Statistical analyses

The data are expressed as the mean ± standard deviation (SD). Data were analyzed using Graph Pad Prism v.6. Statistical significance was assessed using *t*-test or one-way ANOVA followed by Dunnett’s multiple comparisons test or Bonferroni’s multiple comparisons test. *p < 0.05, **p < 0.01, and ***p < 0.001 vs control, ^#^*p* < 0.05, ^##^*p* < 0.01, and ^###^*p* < 0.001 vs synthetic compounds (**4a**-**f** to **6a**-**f**) at the same concentration.

### 2.9. General synthetic methods

All reactions involving moisture-sensitive compounds were conducted under anhydrous conditions (positive argon pressure) using standard syringe, cannula, and septa apparatus. Commercial reagents such as all the methoxy acids, 4-(benzyloxy)aniline hydrochloride, *N*,*N*’-diisopropylcarbodiimide, diisopropylethylamine, sodium borohydride, iodine, lithium hydroxide, palladium on carbon (10%), as were purchased from Sigma, TCI America, Acros, Aapptec, or Chem-Impex and used as received. All solvents were purchased in anhydrous form (Aldrich) and used directly. HPLC-grade hexanes, EtOAc, CH_2_Cl_2_, and MeOH were used in silica gel Combi-Flash chromatography employing a Teledyne Combi*Flash* R_*f*_ 200i instrument with either hexane/EtOAc or CH_2_Cl_2_/MeOH gradients with 1% AcOH for purification of compounds with terminal carboxylic acid groups. Analytical thin-layer chromatography (TLC) was performed using Analtech precoated plates (Uniplate, silica gel GHLF, 250 nm) containing a fluorescence indicator. NMR spectra were recorded using a Varian Inova 500 MHz spectrometer. Coupling constants are reported in Hertz, and peak shifts are reported in *δ* (ppm) relative to CDCl_3_^1^H 7.26 ppm; ^13^C 77.16 ppm), CD_3_OD^1^H 3.31 ppm; ^13^C 49.00 ppm), or dimethyl sulfoxide (DMSO)-*d*_6_^1^H 2.50 ppm, ^13^C 39.52 ppm). Low-resolution mass spectra (ESI) were measured with either an Agilent 260 1200 LC/MSD-SL system or a Shimadzu 2020 LC/MS system. High-resolution mass spectra (HRMS) were obtained by positive ion, ESI analysis on a Thermo Scientific LTQ-XL Orbitrap mass spectrometer with HPLC sample introduction using a short narrow-bore C_18_ reversed-phase column with MeCN/H_2_O gradients. Preparative HPLC of final products was performed using a Waters 2545 binary pump equipped with a reverse-phase Gemini C_18_ column (Phenomenex Inc., pore size: 110 Å, particle size: 10 μm, 250 mm × 21.2 mm) with a gradient of 5–99% MeCN/H_2_O containing 0.1% TFA over 30 min at a flow rate of 10 mL/minutes and monitored with a UV detector at 214 and 254 nm. Semi-preparative HPLC purification was performed using an Agilent 1200 series quaternary pump (MeCN/H_2_O gradient containing 0.1% TFA) with a reverse phase Phenomenex Kinetix-C_18_ column (pore size: 110 Å, particle size: 5 μm, 250 mm × 10.0 mm) at a flow rate 3 mL/minutes and monitored with a UV detection at 210 nm. Fractions containing pure compounds were combined and lyophilized to obtain a white powder. Analytical HPLC of final peptides was performed using an Agilent 1200 series quaternary pump (MeCN/H_2_O gradient containing 0.1% TFA) with a Phenomenex Gemini-C_18_ column (pore size: 110 Å, particle size:5 μm, 250 × 4 mm), at a flow rate of 1 mL/min, and UV detection at 210 nm.

### 2.10. General method A: anhydride-based synthesis of amides 4b – 6b

**Scheme 1.**
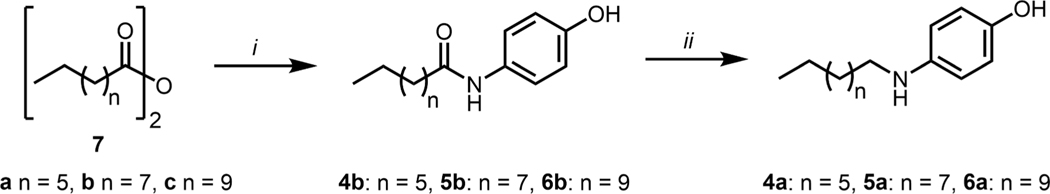
Synthesis of indicated compounds. Reagents and conditions: (*i*) 4-aminophenol, THF; (*ii*) NaBH_4_, I_2_, THF.

To a cold solution of 4-aminophenol (0.109 g, 1.00 mmol) in dry THF (6.9 mL) at 0 °C under N_2_ was added anhydride **7a-7c** (1.10 mmol) and the mixture was allowed to come to room temperature with stirring (16 h). The solvent was removed under reduced pressure and the residue was washed with CHCl_3_ (6.9 mL) and stirred (1 h). The resulting solid was collected and washed to yield **4b-6b** as white crystals (Scheme 1).

#### 2.10.1. *N*-(4-Hydroxyphenol)octanamide (4b).

Synthesis of *N*-(4-hydroxyphenol)octanamide (**4b**) was conducted according to General Method A. The reaction was performed on a 1.00 mmol scale, providing the desired compound in 76% yield (306 mg). HPLC purity: 99%. ^1^H NMR (500 MHz, DMSO-*d*_6_) *δ* 9.56 (s, 1H), 9.12 (s, 1H), 7.37 – 7.30 (m, 2H), 6.71 – 6.60 (m, 2H), 2.25 – 2.18 (m, 2H), 1.60 – 1.51 (m, 2H), 1.31 – 1.19 (m, 8H), 0.90 – 0.80 (m, 3H); ^13^C NMR (125 MHz, DMSO-*d*_6_) *δ* 170.5, 153.1, 131.1, 120.8 (2C), 115.0 (2C), 36.2, 31.2, 28.7, 28.5, 25.3, 22.1, 14.0.

#### 2.10.2. *N*-(4-(Hydroxyphenol)decanamide (5b).

Synthesis of *N*-(4-hydroxyphenol)decanamide (**5b**) was conducted according to General Method A. The reaction was performed on a 1.00 mmol scale, providing the desired compound in 76% yield (306 mg). HPLC purity: 99%. ^1^H NMR (500 MHz, DMSO-*d*_6_) *δ* 9.59 – 9.53 (s, 1H), 9.47 – 9.16 (broad s, 1H), 7.37 – 7.29 (m, 2H), 6.69 – 6.60 (m, 2H), 2.26 – 2.16 (m, 2H), 1.62 – 1.47 (m, 2H), 1.32 – 1.19 (m, 12H), 0.88 – 0.81 (m, 3H); ^13^C NMR (125 MHz, DMSO-*d*_6_) *δ* 170.5, 153.1, 131.0, 120.8 (2C), 115.0 (2C), 36.2, 31.3, 31.28, 28.9, 28.8, 28.7, 25.3, 22.1, 14.0.

#### 2.10.3. *N*-(4-(Hydroxyphenol)decanamide (6b).

Synthesis of *N*-(4-(hydroxyphenol)dodecanamide (**6b)** was conducted according to General Method A. The reaction was performed on a 1.53 mmol scale, providing the desired compound in 86% yield (860 mg). HPLC purity: 99%. ^1^H NMR (500 MHz, DMSO-*d*_6_) *δ* 9.61 – 9.51 (s, 1H), 9.17 – 9.06 (s, 1H), 7.40 – 7.28 (m, 2H), 6.70 – 6.58 (m, 2H), 2.27 – 2.17 (m, 2H), 1.60 – 1.50 (m, 2H), 1.43 – 1.07 (m, 16H), 0.90 – 0.80 (m, 3H); ^13^C NMR (125 MHz, DMSO-*d*_6_) *δ* 170.5, 153.1, 131.1, 120.8 (2C), 115.0 (2C), 36.3, 31.3, 29.04, 29.01, 28.96, 28.8, 28.73, 28.70, 25.3, 22.1, 14.0.

### 2.11. General Method B: Reduction of Amides to Amines 4a, 5a and 6a

To a solution of *p*-alkyl-amidophenol (**4b-6b**) (0.05 g, 0.172 mmol) in anhydrous THF (3 mL, 0.33 M) at 0 °C under N_2_ was added NaBH_4_ (0.016 g, 0.429 mmol, 2.5 eq.) and the reaction mixture was stirred. The suspension was treated dropwise with I_2_ (0.044 g, 0.172 mmol) in anhydrous THF and the reaction mixture was refluxed (3 h) and monitored by TLC then cooled to room temperature. The reaction mixture was treated with 3 M aqueous HCl (2.00 mL, 65.8 mmol), neutralized with 1 M NaOH and extracted with EtOAc. The combined organic extract was washed with H_2_O and brine and dried over Na_2_SO_4_. The solvent was removed under vacuum to provide a crude product, which was purified by silica gel chromatography by elution with EtOAc and hexanes (gradient: 5–30% over 25 min) to yield **4a, 5a** and **6a** as white solids (Scheme 1).

#### 2.11.1. 4-(Octylamino)phenol (4a).

The synthesis of 4-(octylamino)phenol (**4a**) was conducted according to General Method B. The reaction was performed on a 0.651 mmol scale, providing the desired compound in 24% yield (34 mg). HPLC purity: 99%. ^1^H NMR (500 MHz, DMSO-*d*_6_) *δ* 8.33 (s, 1H), 6.56 – 6.48 (m, 2H), 6.45 – 6.38 (m, 2H), 4.79 (s, 1H), 2.92 – 2.82 (m, 2H), 1.54 – 1.43 (m, 2H), 1.38 – 1.18 (m, 10*H*), 0.90 – 0.80 (m, 3H); ^13^C NMR (125 MHz, DMSO-*d*_6_) *δ* 148.0, 142.2, 115.6 (2C), 113.2 (2C), 44.0, 31.3, 28.96, 28.95, 28.7, 26.8, 22.1, 14.0.

#### 2.11.2. 4-(Decylamino)phenol (5a).

Synthesis of 4-(decylamino) phenol (**5a**) was conducted according to General Method B. The reaction was performed on a 1.502 mmol scale, providing the desired compound in 9% yield (33 mg). HPLC purity: 99%. 1H NMR (400 MHz, CDCl_3_) *δ* 6.69 (m, 2H), 6.54 (m, 2H), 3.04 (m, 2H), 1.63 (m, 3H), 1.25 (m, 14H), 0.87 (m, 3H).

#### 2.11.3. 4-(Dodecylamino)phenol (6a).

Synthesis of 4-(dodecylamino)phenol (**6a**) was conducted according to General Method C. The reaction was performed on a 0.172 mmol scale, providing the desired compound in 20% yield (46 mg). HPLC purity: 99%. ^1^H NMR (500 MHz, DMSO-*d*_6_) *δ* 8.37 – 8.29 (s, 1H), 6.58 – 6.48 (m, 2H), 6.44 – 6.33 (m, 2H), 4.85 – 4.72 (s, 1H), 2.91 – 2.83 (m, 2H), 1.53 – 1.44 (m, 2H), 1.40 – 1.15 (m, 18H), 0.91 – 0.79 (m, 3H); ^13^C NMR (125 MHz, DMSO-*d*_6_) *δ* 148.0, 142.1, 115.6 (2C), 113.2 (2C), 44.0, 31.3, 29.08 (2C), 29.03 (2C), 28.99, 28.96, 28.73, 26.8, 22.1, 14.0.

### 2.12. General method C: Coupling reaction for the synthesis of 4d – 6d

**Scheme 2.**
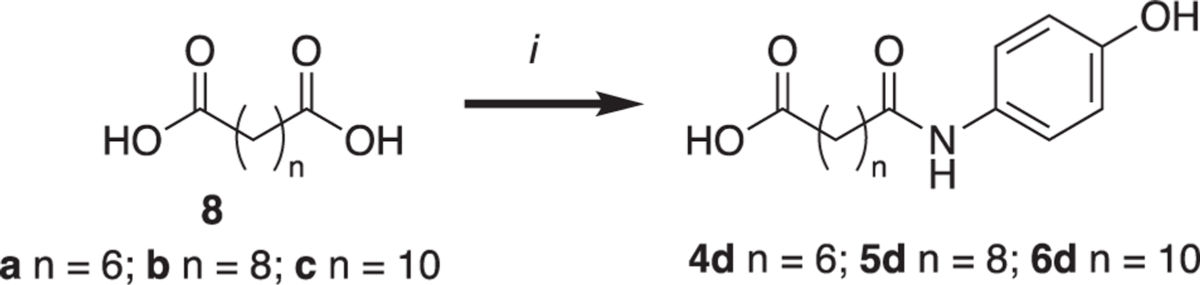
Synthesis of indicated compounds. (*i*) EDC⋅HCl, HABt, DMF.

To a solution of di-carboxylic acid **8** (0.371 g, 1.833 mmol) in DMF (20 mL) and 3*H*–[1,2,3]triazolo[4,5-*b*]pyridin-3-ol (0.499 g, 3.67 mmol) (HABt) and 1-ethyl-3-(3-dimethylaminopropyl)carbodiimide (EDC•HCl) (0.703 g, 3.67 mmol) was added dropwise 4-aminophenol (0.10 g, 0.916 mmol) dissolved in DMF and the reaction mixture was stirred at room temperature under N_2_ (overnight). The solvent was removed under rotary evaporation and the residue was washed with H_2_O and brine and extracted with EtOAc. The organic extract was dried over Na_2_SO_4_ and the solvent was removed under vacuum to provide a crude product, which was purified by silica gel chromatography by elution with 1–5% MeOH in CH_2_Cl_2_ with 1% AcOH to yield of **4d-6d** as white solids (Scheme 2).

#### 2.12.1. 8-((4-Hydroxyphenol)amino)-8-oxododecanoic Acid (4d).

The synthesis of 8-((4-hydroxyphenol)amino)-8-oxododecanoic acid (**4d**) was accomplished using diacid **8a** according to General Method C. The reaction was performed on a 1.83 mmol scale, providing the desired compound in 15% yield (40 mg). HPLC purity: 99%. ^1^H NMR (500 MHz, DMSO-*d*_6_) *δ* 9.62 – 9.53 (s, 1H), 7.36 – 7.31 (m, 2H), 6.70 – 6.62 (m, 2H), 2.26 – 2.14 (m, 4H), 1.59 – 1.43 (m, 4H), 1.34 – 1.23 (m, 4H); ^13^C NMR (125 MHz, DMSO-*d*_6_) *δ* 174.6, 170.5, 153.1, 131.0, 120.8 (2C), 115.0 (2C), 36.2, 33.9, 28.43, 28.37, 25.1, 24.5.

#### 2.12.2. 10-((4-Hydroxyphenol)amino)-10-oxodecanoic Acid (5d).

Synthesis of 10-((4-hydroxyphenol)amino)-10-oxodecanoic acid (**5d**) was accomplished using diacid **8b** according to General Method C. The reaction was performed on a 1.52 mmol scale, providing the desired compound in 7% yield (30 mg). HPLC purity: 99%. ^1^H NMR (500 MHz, DMSO-*d*_6_) *δ* 9.63 – 9.49 (s, 1H), 7.39 – 7.27 (m, 2H), 6.75 – 6.62 (m, 2H), 2.28 – 2.12 (m, 4H), 1.65 – 1.41 (m, 4H), 1.39 – 1.18 (m, 8H); ^13^C NMR (125 MHz, DMSO-*d*_6_) *δ* 174.6, 170.5, 153.1, 131.1, 120.8 (2C), 115.0 (2C), 36.2, 33.8, 28.7, 28.68 (2C), 28.57, 25.2, 24.6.

#### 2.12.3. 12-((4-Hydroxyphenol)amino)-12-oxododecanoic Acid (6d).

Synthesis of 12-((4-hydroxyphenol)amino)-12-oxododecanoic acid (**6d**) was accomplished using diacid **8c** according to General Method C. The reaction was performed on a 0.088 mmol scale, providing the desired compound in 19% yield (28 mg). HPLC purity: 99%. ^1^H NMR (500 MHz, DMSO-*d*_6_) *δ* 9.67 – 9.55 (s, 1H), 7.38 – 7.29 (m, 2H), 6.71 – 6.60 (m, 2H), 2.25 – 2.17 (m, 2H), 2.15 – 2.06 (m, 2H), 1.61 – 1.40 (m, 5H), 1.33 – 1.17 (m, 11*H*); ^13^C NMR (125 MHz, DMSO-*d*_6_) *δ* 174.7, 170.5, 153.1, 131.1, 120.8 (2C), 115.0 (2C), 36.2, 30.7, 28.88, 28.84, 28.76, 28.72, 28.69, 28.66, 25.2, 24.9.

### 2.13. Coupling reaction for the synthesis of 10a – 10c

#### 2.13.1. Methyl 8-((4-(Benzyloxy)phenyl)amino)-8-oxooctanoate (10a).

**Scheme 3.**
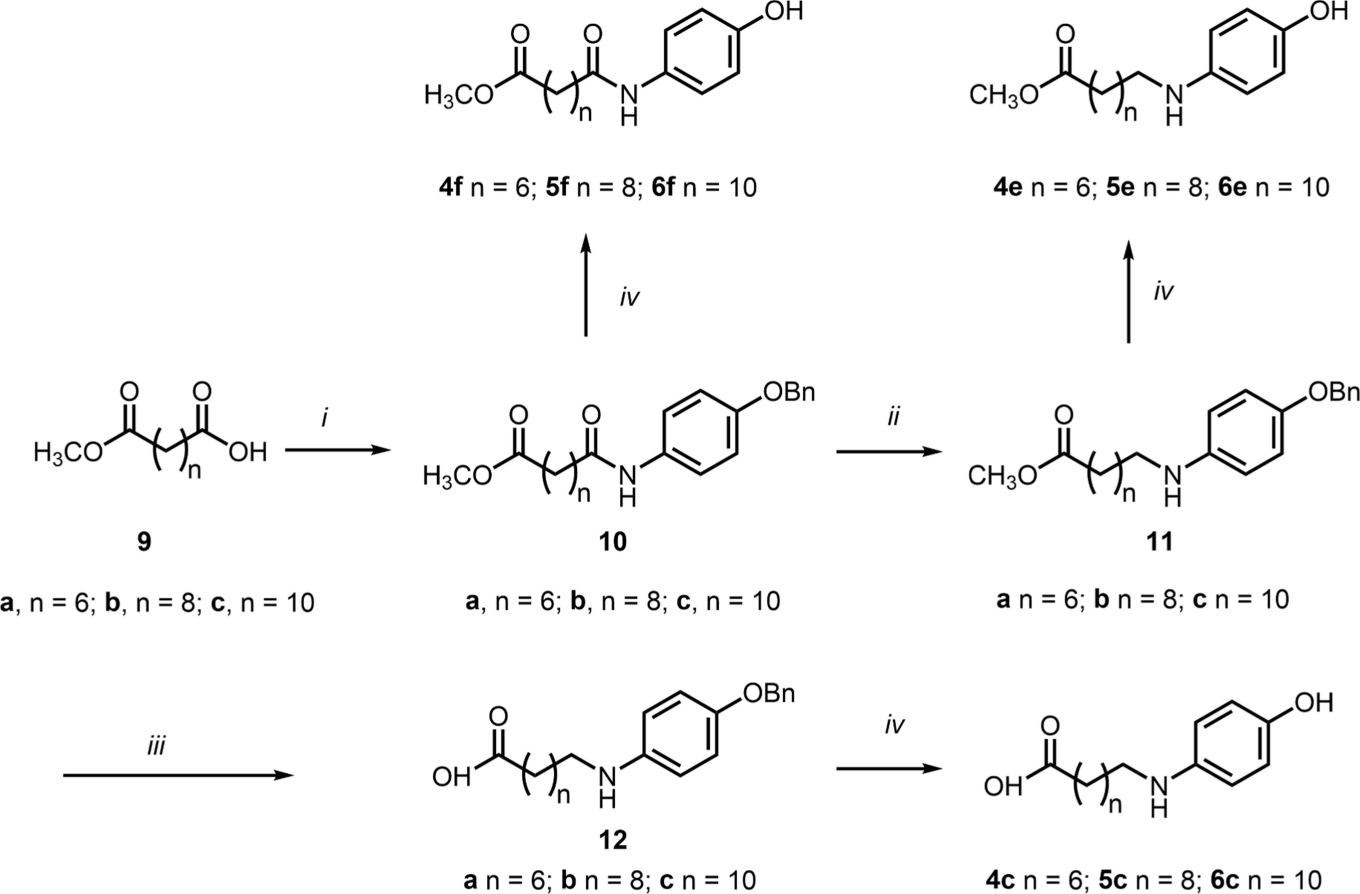
Synthesis of indicated compounds. Reagents and conditions: (*i*) DIC, DIPEA, 4-(benzyloxy)aniline hydrochloride, CH_2_Cl_2_; (*ii*) NaBH_4_, I_2_, THF; (iii) LiOH⋅H_2_O, THF:H_2_O; (*iv*) Pd•C H_2_ (1 atm), MeOH, room temperature.

To a stirred solution of 8-methoxy-8-oxooctanoic acid (**9a**) (1.916 g, 10.18 mmol) in CH_2_Cl_2_ (60 mL) was added *N,N*-diisopropylcarbodiimide (DIC) (0.793 mL, 5.09 mmol) and the reaction mixture was stirred at room temperature until a precipitate is formed (approximately 30 min). To this mixture was added 4-(benzyloxy)aniline hydrochloride (1.2 g, 5.09 mmol) dissolved in CH_2_Cl_2_ (60 mL) and DIPEA (3.56 mL, 20.36 mmol) dropwise. The reaction mixture was stirred under N_2_ at ambient temperature (overnight). The reaction was quenched with 1 N aqueous HCl and extracted with EtOAc and the organic layer was dried over Na_2_SO_4_ and concentrated under vacuum. The residue was purified by silica gel column chromatography using a mixture of 40–60% EtOAc in hexane as eluant to give methyl 8-((4-(benzyloxy)phenyl)amino)-8-oxooctanoate (**10a**) (1.223 g, 65% yield) as a white solid (Scheme 3): R_*f*_ 0.41 (1:4; hexane:EtOAc). ^1^H NMR (500 MHz, DMSO-*d*_6_) *δ* 9.71 (s, 1H), 7.61 – 7.24 (m, 6H), 7.03 – 6.80 (m, 2H), 5.05 (s, 2H), 3.57 (s, 2H), 2.35 – 2.21 (m, 4H), 1.61 – 1.44 (m, 5H), 1.34 – 1.22 (m, 4H); ^13^C NMR (125 MHz, DMSO-*d*_6_) *δ* 173.4, 170.7, 156.8, 154.0, 137.2, 128.4, 127.8, 127.7, 120.5, 114.8, 69.3, 51.2, 36.2, 33.2, 28.3, 25.0, 24.3, 23.3.

#### 2.13.2. Methyl 10-((4-(Benzyloxy)phenyl)amino)-10-oxodecanoate (10b).

Employing the protocol described for the synthesis of **10a**, 4-(benzyloxy)aniline hydrochloride (1.0 g, 4.24 mmol) dissolved in DIPEA (2.96 mL, 16.97 mmol) was reacted with the symmetric anhydride obtained from the reaction of DIC (0.661 mL, 4.24 mmol) and 10-methoxy-10-oxodecanoic acid (**9b**) (1.835 g, 8.48 mmol). The crude residue obtained was purified by silica gel column chromatography using mixture of 40–60% EtOAc in hexane as eluant to give methyl 10-((4-(benzyloxy)phenyl)amino)-10-oxodecanoate (**10b**) (1.214 g, 72% yield) as a white solid (Scheme 3): R_*f*_ 0.45 (1:4; hexane:EtOAc). ^1^H NMR (400 MHz, DMSO-*d*_*6*_) *δ* 9.69 (s, 1H), 7.54 – 7.26 (m, 5H), 6.97 – 6.88 (m, 1H), 5.05 (s, 1H), 3.57 (s, 2H), 2.31 – 2.20 (m, 3H), 1.63 – 1.46 (m, 4H), 1.31 – 1.21 (m, 8H); ^13^C NMR (125 MHz, DMSO-*d*_6_) *δ* 173.4, 170.7, 154.5, 153.6, 137.2, 132.8, 128.4, 127.8, 127.7, 120.5, 114.8, 69.3, 51.2, 36.3, 33.3, 28.62, 28.57, 28.4, 25.2, 24.2.

#### 2.13.3. Methyl 12-((4-(Benzyloxy)phenyl)amino)-12-oxododecanoate (10c).

Employing the protocol described for the synthesis of **10a**, 4-(benzyloxy)aniline hydrochloride (1.0 g, 4.24 mmol) dissolved in DIPEA (2.96 mL, 16.97 mmol) was reacted with the symmetric anhydride obtained from the reaction of DIC (0.661 mL, 4.24 mmol) and 12-methoxy-12-oxododecanoic acid (**9c**) (2.073 g, 8.48 mmol). The crude residue obtained was purified by silica gel column chromatography using a mixture of 40–60% EtOAc in hexane as eluant to give methyl 12-((4-(benzyloxy)phenyl)amino)-12-oxododecanoate (**10c**) (1.228 g, 68% yield) as a white solid (Scheme 3): R_*f*_ 0.48 (1:4; hexane:EtOAc). ^1^H NMR (500 MHz, DMSO-*d*_6_) *δ* 9.71 (s, 1H), 7.51 – 7.45 (m, 2H), 7.45 – 7.41 (m, 2H), 7.41 – 7.35 (m, 2H), 7.35 – 7.29 (m, 1H), 6.96 – 6.89 (m, 2H), 5.05 (s, 2H), 3.57 (s, 3H), 2.32 – 2.18 (m, 3H), 1.61 – 1.43 (m, 4H), 1.33 – 1.18 (m, 12H); ^13^C NMR (125 MHz, DMSO-*d*_6_) *δ* 173.4, 170.7, 156.8, 154.0, 137.2, 132.8, 128.4, 127.8, 127.7, 120.5, 114.8, 69.3, 51.2, 41.4, 36.3, 33.2, 28.9, 28.8, 28.7, 28.5, 25.2, 24.5, 23.3.

### 2.14. Reduction reaction for the synthesis of 11a – 11c

#### 2.14.1. Methyl 8-((4-(Benzyloxy)phenyl)amino)octanoate (11a).

A stirred solution of methyl 8-((4-(benzyloxy)phenyl)amino)-8-oxooctanoate (**10a**) (0.8 g, 2.165 mmol) in THF (10 mL) at 0 °C was treated with NaBH_4_ (0.098 g, 2.60 mmol) in a single portion under N_2_. To this reaction suspension was added I_2_ (0.550 g, 2.165 mmol) in THF (6 mL) dropwise. The ice bath was removed and the reaction mixture was refluxed for 5 h until reaction completion as indicated by TLC. The reaction was quenched by addition of aqueous 1 N HCl and extracted with EtOAc. The combined organic layer was dried over Na_2_SO_4_ and concentrated under vacuum. The resulting residue was purified by column chromatography using a mixture of 60–80% EtOAc in hexane as eluant to give methyl 8-((4-(benzyloxy)phenyl)amino)octanoate (**11a**) (0.292 g, 38% yield) as a white solid (Scheme 3): R_*f*_ 0.30 (1:4; hexane: EtOAc). ^1^H NMR (500 MHz, DMSO-*d*_6_) *δ* 7.44 – 7.32 (m, 4H), 7.32 – 7.26 (m, 1H), 6.80 – 6.73 (m, 2H), 6.52 – 6.45 (m, 3H), 5.11 (s, 1H), 4.94 (s, 2H), 3.57 (s, 3H), 2.90 (t, *J* = 7.0 Hz, 2H), 2.28 (t, *J* = 7.4 Hz, 2H), 1.58 – 1.44 (m, 4H), 1.39 – 1.21 (m, 6H); ^13^C NMR (125 MHz, DMSO-*d*_6_) *δ* 173.4, 149.5, 143.7, 137.8, 128.3, 127.58, 127.56, 115.8, 112.8, 69.8, 51.2, 43.6, 33.3, 28.8, 28.6, 28.5, 26.6, 24.4.

#### 2.14.2. Methyl 10-((4-(Benzyloxy)phenyl)amino)decanoate (11b).

Employing the protocol described for the synthesis of **11a**, methyl 10-((4-(benzyloxy)phenyl)amino)-10-oxodecanoate (**10b**) (0.65 g, 1.635 mmol) was treated with NaBH_4_ (0.074 g, 1.962 mmol) and I_2_ (0.415 g, 1.635 mmol). The crude residue obtained was purified by silica gel flash column chromatography using a mixture of 60–80% EtOAc in hexane as eluant to give methyl 10-((4-(benzyloxy)phenyl)amino)decanoate (**11b**) (0.219 g, 35% yield) as white solid (Scheme 3): R_*f*_ 0.32 (1:4; hexane:EtOAc). ^1^H NMR (400 MHz, DMSO-*d*_6_) *δ* 7.43 – 7.33 (m, 4H), 7.33 – 7.27 (m, 1H), 6.81 – 6.71 (m, 2H), 6.54 – 6.45 (m, 2H), 5.07 (s, 1H), 4.95 (s, 2H), 3.57 (s, 3H), 2.91 (t, *J* = 7.0 Hz, 2H), 2.28 (t, *J* = 7.4 Hz, 2H), 1.58 – 1.44 (m, 4H), 1.39 – 1.20 (m, 10*H*); ^13^C NMR (125 MHz, DMSO-*d*_6_) *δ* 173.4, 149.5, 143.6, 137.8, 128.3, 127.59, 127.56, 115.8, 112.9, 69.8, 51.2, 43.7, 33.3, 28.9, 28.88, 28.80, 28.7, 28.5, 29.7, 24.4.

#### 2.14.3. Methyl 12-((4-(Benzyloxy)phenyl)amino)dodecanoate (11c).

Employing the protocol described for the synthesis of **11a**, methyl 12-((4-(benzyloxy)phenyl)amino)-12-oxododecanoate (**10c**) (0.6 g, 1.410 mmol) was treated with NaBH_4_ (0.064 g, 1.692 mmol) and I_2_ (0.358 g, 1.410 mmol) and the crude residue obtained was purified by silica gel flash column chromatography using a mixture of 60–80% EtOAc in hexane as eluant to give methyl 12-((4-(benzyloxy)phenyl) amino)dodecanoate (**11c**) (0.186 g, 32% yield) as a white solid (Scheme 3): R_*f*_ 0.35 (1:4; hexane:EtOAc). ^1^H NMR (500 MHz, DMSO-*d*_6_) *δ* 7.43 – 7.33 (m, 4H), 7.33 – 7.26 (m, 1H), 6.80 – 6.73 (m, 2H), 6.53 – 6.41 (m, 2H), 5.08 (t, *J* = 5.7 Hz, 1H), 4.95 (s, 2H), 3.57 (s, 3H), 2.95 – 2.85 (m, 2H), 2.28 (t, *J* = 7.4 Hz, 2H), 1.55 – 1.44 (m, 4H), 1.39 – 1.19 (m, 14H); ^13^C NMR (125 MHz, DMSO-*d*_6_) *δ* 173.4, 149.4, 143.7, 137.8, 128.3, 127.58, 127.56, 115.8, 112.8, 69.8, 51.2, 43.6, 33.3, 29.1, 28.96 (2C), 28.9, 28.8, 28.7, 28.5, 26.8, 24.5.

### 2.15. Saponification reaction for the synthesis of 12a – 12c

#### 2.15.1. 8-((4-(Benzyloxy)phenyl)amino)octanoic Acid (12a).

A stirred solution of methyl 8-((4-(benzyloxy)phenyl)amino)octanoate (**11a**) (0.32 g, 0.900 mmol) in THF (3.2 mL)) at 0 °C was treated with LiOH⋅H_2_O (0.108 g, 4.5 mmol) dissolved into H_2_O (3.20 mL) in a single portion under N_2_. The reaction mixture was allowed to come to room temperature and stirred (overnight). The reaction was quenched with aqueous 1 N HCl and extracted with EtOAc and the organic layer was dried over Na_2_SO_4_ and concentrated under vacuum. The residue was purified by silica gel flash column chromatography using a mixture of EtOAc/ hexane/AcOH 50:49:1 (v/v/v) as eluant to give 8-((4-(benzyloxy)phenyl)amino)octanoic acid (**12a**) (0.086 g, 28% yield) as a white solid (Scheme 3): ^1^H NMR (500 MHz, DMSO-*d*_6_) *δ* 11.97 (s, 1H), 9.80 (s, 1H), 7.31 – 7.24 (m, 1H), 7.24 – 7.14 (m, 2H), 7.08 – 6.93 (m, 2H), 6.90 – 6.78 (m, 2H), 6.65 – 6.42 (m, 2H), 3.87 (s, 2H), 3.12 (t, *J* = 7.7 Hz, 2H), 2.18 (t, *J* = 7.4 Hz, 2H), 1.57 – 1.41 (m, 3H), 1.34 – 1.17 (m, 5H); ^13^C NMR (125 MHz, DMSO-*d*_6_) *δ* 174.5, 158.4, 141.6, 129.9, 128.7, 128.3, 125.9, 115.8, 40.1 (2C), 35.0, 33.6, 28.33.28.28, 25.7, 24.4.

#### 2.15.2. 10-((4-(Benzyloxy)phenyl)amino)decanoic Acid (12b).

Employing the protocol described for the synthesis of **12a**, methyl 10-((4-(benzyloxy)phenyl)amino)decanoate (**11**b) (0.3 g, 0.782 mmol) was treated with LiOH⋅H_2_O (0.019 g, 0.782 mmol) and the crude residue obtained was purified by column chromatography using a mixture of EtOAc/ hexane/AcOH 50:49:1 (v/v/v) as eluant to give 10-((4-(benzyloxy)phenyl)amino)decanoic acid (**12b**) (0.075 g, 26% yield) as white solid (Scheme 3): ^1^H NMR (500 MHz, DMSO-*d*_6_) *δ* 7.43 – 7.33 (m, 4H), 7.33 – 7.27 (m, 1H), 6.80 – 6.73 (m, 2H), 6.51 – 6.45 (m, 2H), 4.95 (s, 2H), 2.90 (t, *J* = 7.1 Hz, 2H), 2.18 (t, *J* = 7.4 Hz, 2H), 1.54 – 1.44 (m, 4H), 1.37 – 1.28 (m, 3H), 1.26 (s, 7H); ^13^C NMR (125 MHz, DMSO-*d*_6_) *δ*173.9, 158.0, 136.7, 128.5, 128.4, 128.0, 127.77, 127.66, 124.0, 115.8, 69.6, 33.7, 28.7, 28.6, 28.54, 28.50, 28.46, 25.8, 25.3, 24.5, 23.3.

#### 2.15.3. 12-((4-(Benzyloxy)phenyl)amino)dodecanoic Acid (12c).

Employing the protocol described for the synthesis of **12a**, methyl 12-((4-(benzyloxy)phenyl)amino)dodecanoate (**11**c) (0.18 g, 0.462 mmol) was treated with LiOH⋅H_2_O (0.011 g, 0.462 mmol) and the crude residue obtained was purified by silica gel flash column chromatography using a mixture of EtOAc/ hexane/AcOH 50:49:1 (v/v/v) as eluant to give 12-((4-(benzyloxy)phenyl)amino)dodecanoic acid (**12c**) (0.061 g, 33.4% yield) as a white solid (Scheme 3) and was taken forward for the next reaction without any further purification.

### 2.16. Hydrogenation reactions

#### 2.16.1. 8-((4-Hydroxyphenyl)amino)octanoic Acid (4c).

A solution of 8-((4-(benzyloxy)phenyl)amino)octanoic acid (**12a**) (50 mg, 0.146 mmol) in MeOH (1 mL) was treated carefully with 10 wt% Pd•C (15 mg) under a stream of argon. The resulting suspension was placed under hydrogen atmosphere (1 atm), stirred under a balloon of hydrogen at room temperature for 2–3 h, and filtered through Celite, which was washed with MeOH. The filtrate and washings were combined and taken to dryness under high vacuum to furnish crude product as an off-white solid. This was purified by HPLC to provided 8-((4-hydroxyphenyl)amino)octanoic acid (**4c)** (8.10 mg, 22% yield) as a lyophilized white powder (Scheme 3): ^1^H NMR (500 MHz, CD_3_OD) *δ* 7.32 – 7.23 (m, 2H), 6.95 – 6.87 (m, 2H), 3.29 – 3.27 (m, 2H) 2.28 (t, *J* = 7.4 Hz, 2H), 1.74 – 1.65 (m, 2H), 1.65 – 1.55 (m, 2H), 1.47 – 1.30 (m, 6H); ^13^C NMR (125 MHz, CD_3_OD) *δ* 177.6, 160.0, 127.9, 124.8, 117.7, 53.5, 34.8, 29.9, 29.8, 27.2, 26.9, 25.8. HPLC [10–95% ACN (0.1% TFA) in water (0.1% TFA) over 27 min, then at 10% ACN (0.1% TFA) in water (0.1% TFA) for 3 min] Rt = 19.26.

#### 2.16.2. 10-((4-Hydroxyphenyl)amino)decanoic Acid (5c).

Employing the protocol described for the synthesis of **4c**, 10-((4-(benzyloxy)phenyl)amino)decanoic acid (**12b**) (40 mg, 0.108 mmol) was treated carefully with 10 wt% Pd•C (12 mg) under a stream of argon and the resulting crude sample was purified by HPLC to provide 10-((4-hydroxyphenyl)amino)decanoic acid (**5c)** (5.44 mg, 18% yield) as a lyophilized white powder (Scheme 3): ^1^H NMR (400 MHz, DMSO-*d*_6_) *δ* 9.72 (s, 1H), 7.30 – 7.06 (m, 2H), 6.92 – 6.72 (m, 2H), 3.17 (t, *J* = 7.7 Hz, 2H), 2.19 (t, *J* = 7.4 Hz, 2H), 1.63 – 1.42 (m, 4H), 1.39 – 1.17 (m, 10*H*); ^13^C NMR (125 MHz, CD_3_OD) *δ* 177.7, 159.7, 128.5, 124.5, 117.7, 53.3, 34.9, 30.23, 30.21, 30.13, 30.11 27.4, 27.1, 26.0. HPLC [10–95% ACN (0.1% TFA) in water (0.1% TFA) over 27 min, then at 10% ACN (0.1% TFA) in water (0.1% TFA) for 3 min] Rt = 20.77.

#### 2.16.3. 12-((4-Hydroxyphenyl)amino)dodecanoic Acid (6c).

Employing the protocol described for the synthesis of **4c**, 12-((4-(benzyloxy)phenyl)amino)dodecanoic acid (**12**c) (20 mg, 0.059 mmol) was treated carefully with 10 wt% Pd•C (6 mg) and the resulting crude sample was purified by HPLC to provide 12-((4-hydroxyphenyl)amino) dodecanoic acid (**6c**) (9 mg, 61% yield) as a lyophilized white powder (Scheme 3): HPLC Purity 99%. ^1^H NMR (500 MHz, CD_3_OD) *δ* 7.38 (m, 2H), 6.90 (m, 2H), 2.19 (m, 2H), 1.51 (m, 5H), *δ* 1.25 (m, 15H); ^13^C NMR (500 MHz, CD_3_OD) *δ* 176.3, 158.0, 122.9, 116.3 (2C), 51.7 (2C), 33.5 (2C), 33.5 (2C), 28.8 (2C), 26.0 (2C), 24.7 (2C). HPLC [10–95% ACN (0.1% TFA) in water (0.1% TFA) over 27 min, then at 10% ACN (0.1% TFA) in water (0.1% TFA) for 3 min] Rt = 22.28.

#### 2.16.4. Methyl 8-((4-Hydroxyphenyl)amino)-8-oxooctanoate (4f).

Employing the protocol described for the synthesis of **4c**, methyl 8-((4-(benzyloxy)phenyl)amino)-8-oxooctanoate (**10a**) (0.1 g, 0.271 mmol) was treated with 10 wt% Pd•C (30 mg). The resulting crude sample was purified by HPLC to provide methyl 8-((4-hydroxyphenyl) amino)-8-oxooctanoate (**4f**) (0.015 g, 20% yield) as a lyophilized white powder (Scheme 3): ^1^H NMR (500 MHz, DMSO-*d*_6_) *δ* 9.58 (s, 1H), 9.12 (s, 1H), 7.34 (d, *J* = 8.9 Hz, 2H), 6.66 (d, *J* = 8.9 Hz, 2H), 3.57 (s, 3H), 2.29 (t, *J* = 7.4 Hz, 2H), 2.22 (s, 2H), 1.60 – 1.46 (m, 4H), 1.34 – 1.22 (m, 4H); ^13^C NMR (125 MHz, DMSO-*d*_6_) *δ* 173.4, 170.4, 153.1, 131.1, 120.8, 115.0, 51.2, 36.2, 33.2, 28.4, 28.3, 25.1, 24.3. HPLC [10–95% ACN (0.1% TFA) in water (0.1% TFA) over 27 min, then at 10% ACN (0.1% TFA) in water (0.1% TFA) for 3 min] Rt = 18.21.

#### 2.16.5. Methyl 10-((4-Hydroxyphenyl)amino)-10-oxodecanoate (5f).

Employing the protocol described for the synthesis of **4c**, methyl 10-((4-(benzyloxy)phenyl)amino)-10-oxodecanoate (**10b**) (0.1 g, 0.252 mmol)) was treated with 10 wt% Pd•C (30 mg). The resulting crude sample was purified by HPLC to provide methyl 10-((4-hydroxyphenyl) amino)-10-oxodecanoate (**5f**) (0.014 g, 18% yield) as a lyophilized white powder (Scheme 3): ^1^H NMR (500 MHz, DMSO-*d*_6_) *δ* 9.57 (s, 1H), 9.13 (s, 1H), 7.38 – 7.28 (m, 2H), 6.71 – 6.56 (m, 2H), 3.57 (s, 3H), 2.28 (t, *J* = 7.4 Hz, 2H), 2.25 – 2.09 (m, 2H), 1.62 – 1.41 (m, 4H), 1.34 – 1.15 (m, 8H); ^13^C NMR (125 MHz, DMSO- *d*_6_) *δ* 173.4, 170.5, 153.1, 131.1, 120.8, 115.0, 51.2, 36.2, 33.3, 28.67, 28.65, 28.60, 28.4, 25.2, 24.4. HPLC [10–95% ACN (0.1% TFA) in water (0.1% TFA) over 27 min, then at 10% ACN (0.1% TFA) in water (0.1% TFA) for 3 min] Rt = 20.76.

#### 2.16.6. Methyl 12-((4-Hydroxyphenyl)amino)-12-oxododecanoate (6f).

Employing the protocol described for the synthesis of **4c**, methyl 12-((4-(benzyloxy)phenyl)amino)-12-oxododecanoate (**6c**) (0.1 g, 0.235 mmol) was treated with 10 wt% Pd•C (30 mg). The resulting crude sample was purified by HPLC to methyl 12-((4-hydroxyphenyl) amino)-12-oxododecanoate (**6f**) (0.012 g, 15% yield) as a lyophilized white powder (Scheme 3): ^1^H NMR (500 MHz, DMS-*d*_6_) *δ* 9.57 (s, 1H), 9.12 (s, 1H), 7.38 – 7.29 (m, 2H), 6.70 – 6.59 (m, 2H), 3.57 (s, 3H), 2.28 (t, *J* = 7.4 Hz, 2H), 2.22 (t, *J* = 7.4 Hz, 2H), 1.61 – 1.43 (m, 4H), 1.33 – 1.18 (m, 12H); ^13^C NMR (125 MHz, DMSO-*d*_6_) *δ* 173.4, 170.5, 153.1, 131.1, 120.8, 115.0, 51.2, 36.3, 33.3, 28.88, 28.86, 28.79, 28.70, 28.67, 28.5, 25.3, 24.5. HPLC [10–95% ACN (0.1% TFA) in water (0.1% TFA) over 27 min, then at 10% ACN (0.1% TFA) in water (0.1% TFA) for 3 min] Rt = 23.18.

#### 2.16.7. Methyl 8-((4-Hydroxyphenyl)amino)octanoate (4e).

Employing the protocol described for the synthesis of **4c**, methyl 8-((4-(benzyloxy)phenyl)amino)octanoate (**11a**) (50 mg, 0.141 mmol) was treated with 10 wt% Pd•C (15 mg) The resulting crude sample was purified by HPLC to provide methyl 8-((4-hydroxyphenyl)amino)octanoate (**4e**) (4.48 mg, 12% yield) as a lyophilized white powder (Scheme 3): ^1^H NMR (400 MHz, DMSO-*d*_6_) *δ* 9.69 (s, 1H), 7.21 – 7.05 (m, 2H), 6.91 – 6.74 (m, 2H), 3.57 (s, 3H), 3.16 (t, *J* = 7.7 Hz, 2H), 2.29 (t, *J* = 7.4 Hz, 2H), 1.61 – 1.45 (m, 4H), 1.41 – 1.15 (m, 6H); ^13^C NMR (125 MHz, CD_3_OD) *δ* 175.9, 160.0, 128.0, 124.8, 117.7, 53.4, 52.0, 34.6, 29.81, 29.80, 27.2, 27.0, 25.8. HPLC [10–95% ACN (0.1% TFA) in water (0.1% TFA) over 27 min, then at 10% ACN (0.1% TFA) in water (0.1% TFA) for 3 min] Rt = 14.31.

#### 2.16.8. Methyl 10-((4-Hydroxyphenyl)amino)decanoate (5e).

Employing the protocol described for the synthesis of **4c**, methyl 10-((4-(benzyloxy)phenyl)amino)decanoate (**11b**) (50 mg, 0.130 mmol) was treated with 10 wt% Pd•C (15 mg).The resulting crude sample was purified by HPLC to provide methyl 10-((4-hydroxyphenyl)amino)decanoate (**5e**) (3.83 mg, 10% yield) as a lyophilized white powder (Scheme 3): ^1^H NMR (400 MHz, DMSO-*d*_6_) *δ* 9.72 (s, 1H), 7.16 (m, 2H), 6.87 – 6.76 (m, 2H), 3.57 (s, 3H), 3.16 (t, *J* = 7.7 Hz, 2H), 2.28 (t, *J* = 7.4 Hz, 2H), 1.61 – 1.42 (m, 4H), 1.25 (d, *J* = 6.2 Hz, 10*H*); ^13^C NMR (125 MHz, CD_3_OD) *δ* 176.0, 159.8, 128.3, 124.6, 117.7, 53.4, 52.0, 34.7, 30.21, 30.17, 30.10, 30.08, 27.4, 27.1, 26.0. HPLC [10–95% ACN (0.1% TFA) in water (0.1% TFA) over 27 min, then at 10% ACN (0.1% TFA) in water (0.1% TFA) for 3 min] Rt = 16.19.

#### 2.16.9. Methyl 12-((4-Hydroxyphenyl)amino)dodecanoate (6e).

Employing the protocol described for the synthesis of **4c**, methyl 12-((4-(benzyloxy)phenyl)amino)dodecanoate (**11c**) (50 mg, 0.121 mmol) was treated with 10 wt% Pd•C (15 mg).The resulting crude sample was purified by HPLC to provide methyl 12-((4-hydroxyphenyl)amino) dodecanoate (**6e**) (3.12 mg, 8% yield) as a lyophilized white powder (Scheme 3): ^1^H NMR (500 MHz, DMSO-*d*_6_) *δ* 9.69 (s, 1H), 7.20 – 7.07 (m, 2H), 6.88 – 6.74 (m, 2H), 3.57 (s, 3H), 3.48 – 3.38 (m, 4H), 3.15 (t, *J* = 7.6 Hz, 2H), 2.28 (t, *J* = 7.4 Hz, 2H), 1.59 – 1.45 (m, 4H), 1.35 – 1.19 (m, 10*H*); ^13^C NMR (125 MHz, CD_3_OD) *δ* 176.0, 159.8, 128.2, 124.6, 117.7, 53.4, 52.0, 49.6, 34.8, 30.5, 30.4, 30.3, 30.19, 30.16, 27.4, 27.1, 26.0. HPLC [10–95% ACN (0.1% TFA) in water (0.1% TFA) over 27 min, then at 10% ACN (0.1% TFA) in water (0.1% TFA) for 3 min] Rt = 17.82.

## 3. Results

Breast and prostate cancers are cancer types with very high prevalence in women and men. Currently, chemotherapy is often given in addition to surgical resection as treatment for both cancers. Cyclophosphamide, doxorubicin, and fluorouracil are used as therapeutic agents for these cancers, and docetaxel is used for prostate cancer. However, it is known that treatment with these anticancer agents causes side effects such as myelosuppression that increases in the risk of infectious diseases. In order to potentially address these problems, we evaluated the growth inhibitory effects of the new compound against these two types of cancer cells.

### 3.1. Effects on growth of human breast and prostate cancer cells inserting carboxylic acid functionality into p-alkylaminophenols

**Fig. 3.**
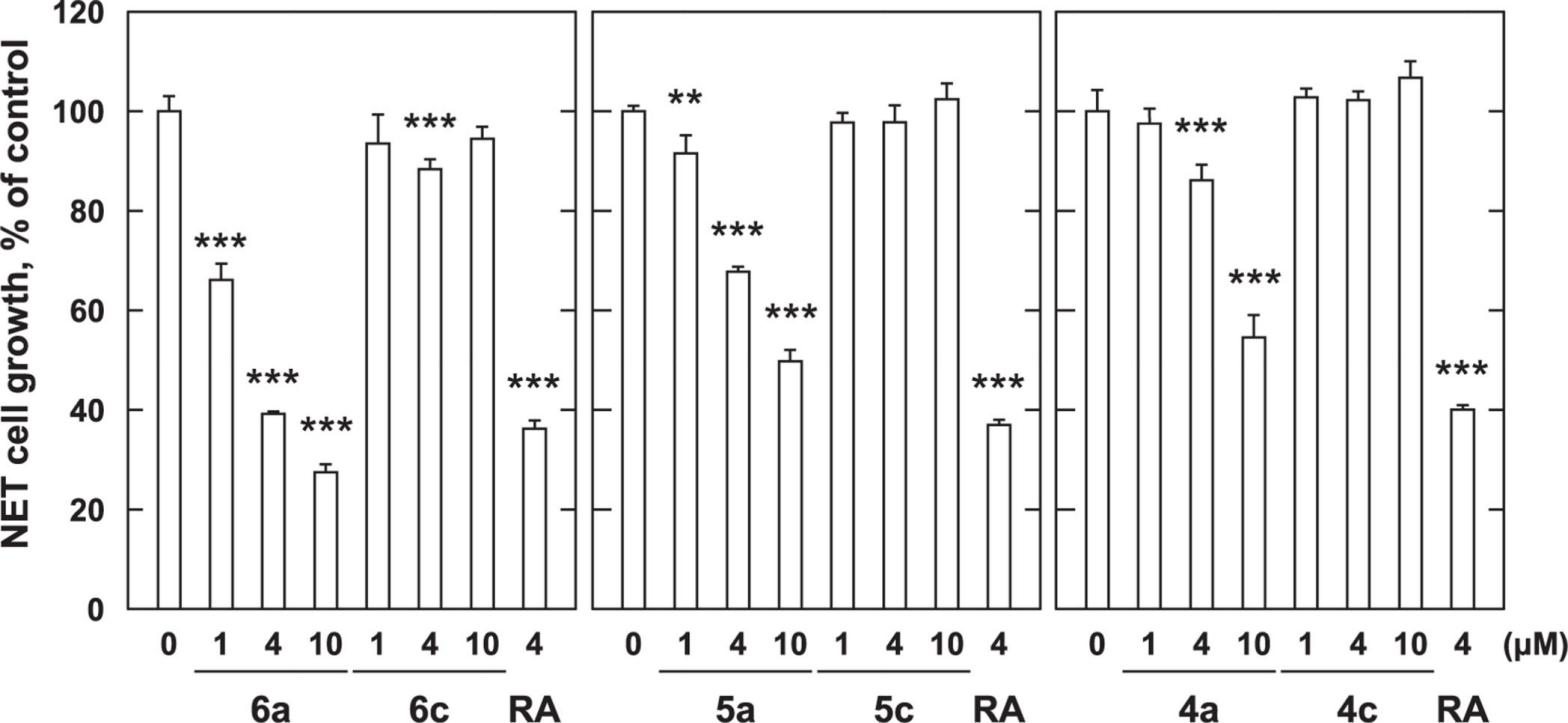
Growth inhibition with *p*-aminophenol derivatives against MCF-7 cells. MCF-7 cells (2 × 10^4^ cells/mL) were treated with various concentration of *p*-aminophenol derivatives for 72 h. Cell viability was determined by MTT method. Data shown are mean ± SD (*n* = 3). *p < 0.05, **p < 0.01, ***p < 0.001 vs Control (Dunnett’s multiple comparisons test).

First, we investigated the growth-inhibitory effects against MCF-7 cells of three *p*-alkylaminophenols (**4a, 5a**, and **6a**) with different carbon chain-lengths. All *p*-alkylaminophenols significantly suppressed the growth of MCF-7 cells in a concentration-dependent manner as compared to the control (Fig. 3). Among the three *p*-alkylaminophenols, analog **6a** having the longest carbon chain length, had the greatest growth inhibitory potency. The inhibition percentages at 4 μM and 10 μM concentrations were approximately 61% and 73% for **6a,** 31% and 51% for **5a**, and 14% and 45% for **4a**, with 4 μM RA exhibiting approximately 62%. Therefore, at 4 μM **6a** showed a cell proliferation inhibitory potency that was equal to RA. In contrast, insertion of a carboxylic acid moiety at the terminus of the polymethylene chains of **4a**, **5a**, and **6a** to give the corresponding acids **4c**, **5c**, and **6c** uniformly abrogated growth inhibitory potencies (Fig. 3).

**Fig. 4.**
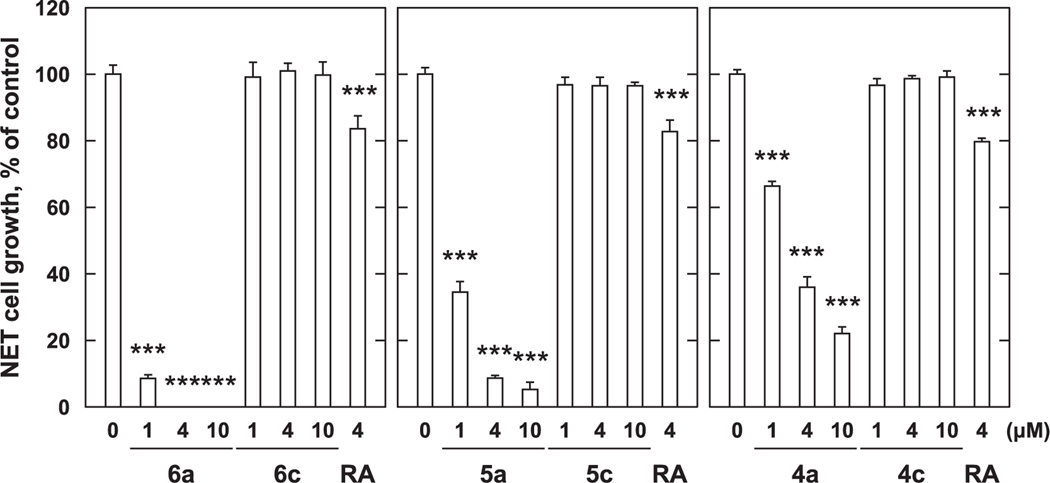
Growth inhibition with *p*-aminophenol derivatives against PC-3 cells. PC-3 cells (2 × 10^4^ cells/mL) were treated with various concentration of *p*-aminophenol derivatives for 72 h. Cell viability was determined by MTT method. Data shown are mean ± SD (*n* = 3). *p < 0.05, **p < 0.01, ***p < 0.001 vs Control (Dunnett’s multiple comparisons test).

The growth inhibitory effects of synthetic compounds against PC-3 cells were investigated. The three parent *p*-alkylaminophenols (**4a**, **5a**, and **6a**) significantly suppressed cell proliferation in concentration-dependent manner as compared to controls (Fig. 4). At a concentration of 1 μM, **6a** inhibited cell proliferation by approximately 91%, **5a** by 65%, and **4a** by 33%. Similar to the results seen with MCF-7 cells, compound **6a**, which has the longest chain length, showed the greatest potency. In contrast, the growth inhibitory potency of 4 μM RA of approximately 18%, was significantly weaker effect than the *p*-alkylaminophenols. Similar to what was observed with MCF-7 breast cancer cells, against PC-3 cells, compounds **4c**, **5c**, and **6c**, which have terminal carboxyl groups, did not show significant growth inhibitory effects. We also found that the parent *p*-alkylaminophenols (**4a** – **6a**) more potently inhibited the proliferation of PC-3 cells than was observed with MCF-7 cells. These results suggest that the *p*-alkylaminophenols exhibit cell proliferation inhibitory effects against both MCF-7 and PC-3 cells with potencies that are in the order **6a** > **5a** > **4a**. In contrast insertion of a chain-terminating carboxylic acid group abrogated the growth inhibitory effects against both cell lines.

### 3.2. Effects on growth of human breast and prostate cancer cells inserting carboxylic acid functionality into p-amidophenols

**Fig. 5.**
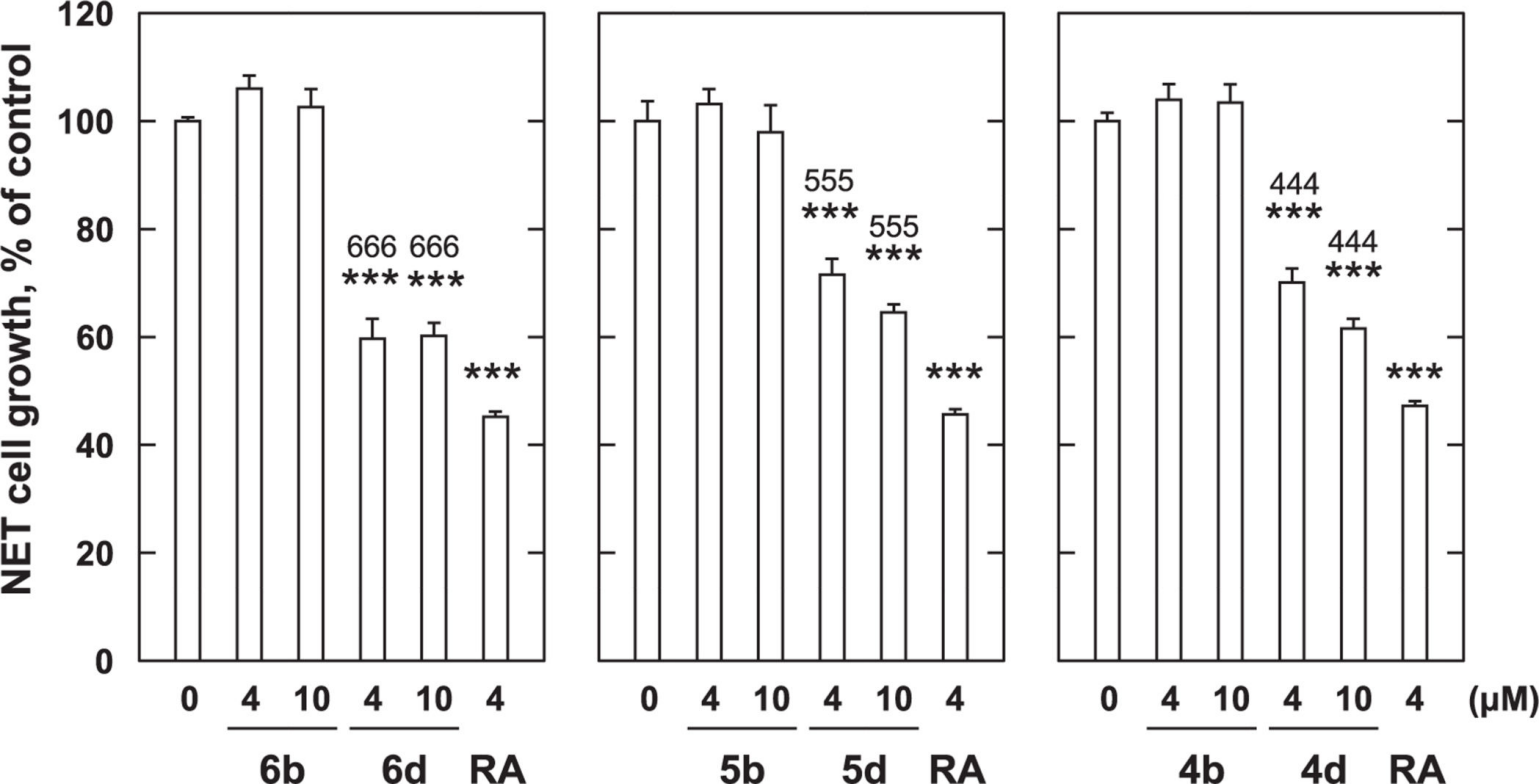
Growth inhibition with *p*-amidophenol derivatives against MCF-7 cells. MCF-7 cells (4 × 10^4^ cells/mL) were treated with various concentration of *p*-amidophenol derivatives for 72 h. Cell viability was determined by MTT method. Data shown are mean ± SD (*n* = 3). ***p < 0.001 vs Control, ^666^*p* < 0.001 vs the **6b** at the same concentration, ^555^*p* < 0.001 vs the **5b** at the same concentration, ^444^*p* < 0.001 vs the **4b** at the same concentration (Bonferroni’s multiple comparisons test).

Next, we investigated the growth inhibitory effects of *p*-amidophenols (**4b**, **5b**, and **6b**) on MCF-7 and PC-3 cells. We found that against MCF-7 cells, conversion of the alkylamine groups of **4b**, **5b**, and **6b** to the corresponding alkylamido groups, resulted in a significant loss of cell growth inhibitory potencies. In contrast, inserting chain-terminal carboxyl groups into the *p*-amidophenols to give the corresponding carboxyl-containing amidophenols (**4d**, **5d**, and **6d**) resulted in a gain of growth inhibitory potencies (Fig. 5). At a concentration of 4 μM, significant cell-growth inhibitory potencies were observed. When expressed in terms of suppressing percentages as compared to control, growth inhibitory values were approximately 40% for **6d**, 28% for **5d**, and 30% for **4d**, while 4 μM RA showed approximately 52% growth inhibition.

**Fig. 6.**
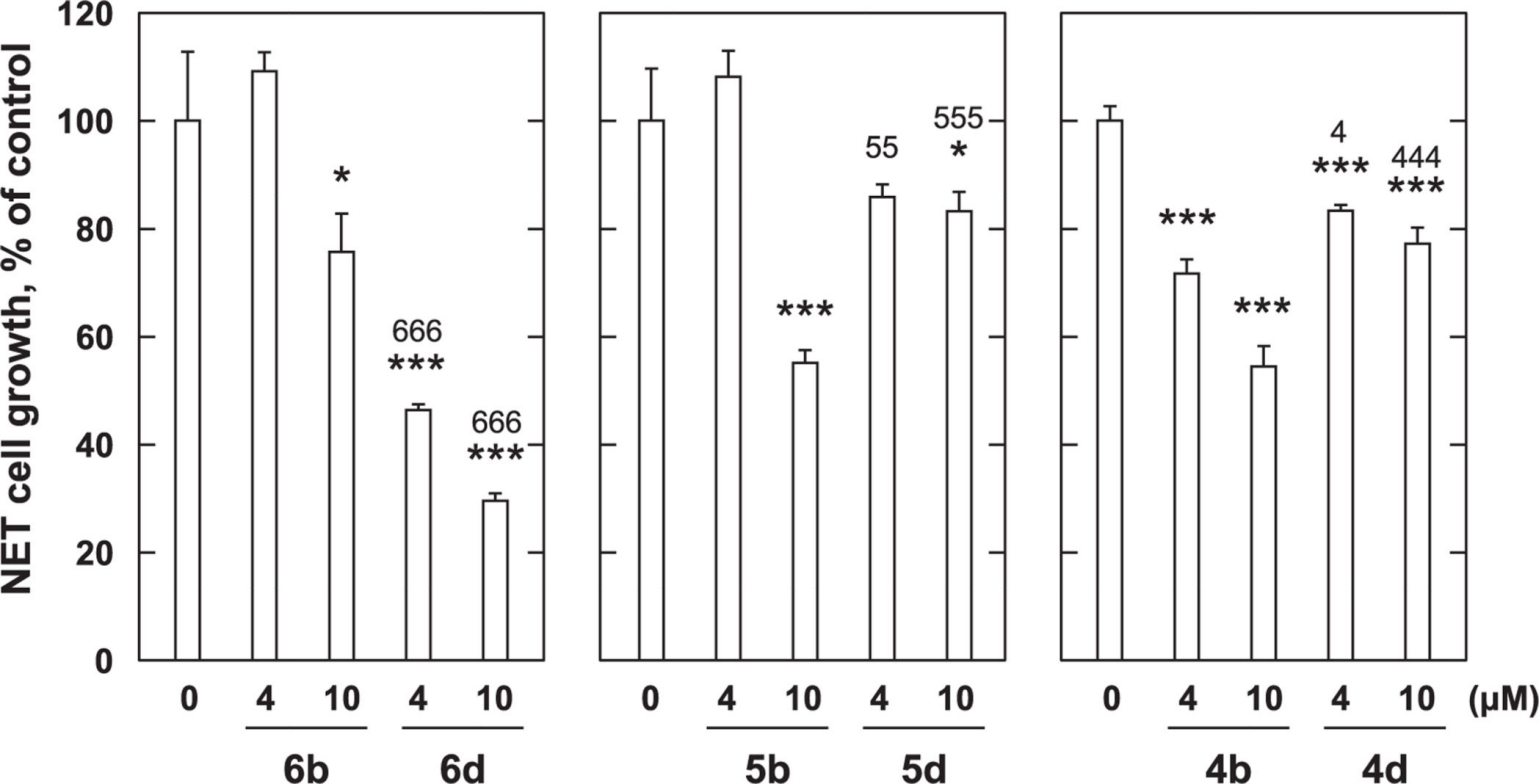
Growth inhibition with *p*-amidophenol derivatives against PC-3 cells. PC-3 cells (4 × 10^4^ cells/mL) were treated with various concentration of *p*-amidophenol derivatives for 72 h. Cell viability was determined by MTT method. Data shown are mean ± SD (*n* = 3). *p < 0.05, ***p < 0.001 vs Control, ^666^*p* < 0.001 vs the **6b** at the same concentration, ^55^*p* < 0.01, ^555^*p* < 0.001 vs the **5b** at the same concentration, ^4^*p* < 0.05, ^444^*p* < 0.001 vs the **4b** at the same concentration (Bonferroni’s multiple comparisons test).

Next, we examined the effects of synthetic compounds on PC-3 cell proliferation. While we found that *p*-amidophenols **6b**, **5b**, and **4b** did not show significant growth inhibitory effects against MCF-7 cells, they did show moderate growth inhibitory effects against PC-3 cells as compared to controls (Fig. 6). At a concentration of 10 μM, compound **6b** showed cell-suppressing effect as compared to the control at levels of 24%, **5b** 43%, and **4b** 45%, with the potencies being in the order **6b** < **5b** < **4b** (Fig. 6). Inserting a terminal carobxyl group into **6**b to yield **6**d, enhanced cell growth inhibitory potency (approximately 54% inhibition at 4 μM and 70% inhibition at 10 μM). In contrast, similar structural modifications to **5**b, and **4**b to yield **5**d and **4**d attenuated growth inhibitory potencies (Fig. 6). Under the assay conditions, treatment with 4 μM RA inhibited PC-3 cell growth by approximately 19% (data not shown).

As mentioned above, while the *p*-amidophenols **6b**, **5b**, and **4b** did not significantly inhibit the proliferation of MCF-7 cells, they did have antiproliferative effects against PC-3 cells. Proliferation of PC-3 cells were suppressed by **5b** and **4b** with short chains greater than by **6b** with a longer chain. Compounds **4d, 5d**, and **6d** having a carboxyl group appended onto the terminal chain of the *p*-amidophenols **4b**, **5b** and **6b**, showed enhanced growth inhibitory potencies against MCF-7 cells. However, against PC-3 cells, the inhibitory potencies of **4d** and **5d** were reduced, while the potency of **6d** was enhanced. These results indicate that the *p*-amidophenols suppressed the proliferation of PC-3 cells in the order **6**b < **5**b < **4**b but did not significantly affect MCF-7 cells. Furthermore, against MCF-7 cell, the variants **4d**, **5d**, and **6d** having chain-terminal carboxyl groups showed enhanced inhibitory potencies, while against PC-3 cells the compounds showed enhanced (**6d**) or attenuated potencies (**4d** and **5d**) as compared to the non-carboxyl-containing congeners.

### 3.3. Growth inhibitory effects of methyl ester-containing p-aminophenols 4e – 6e and p-amidophenols 4f – 6f.

**Fig. 7.**
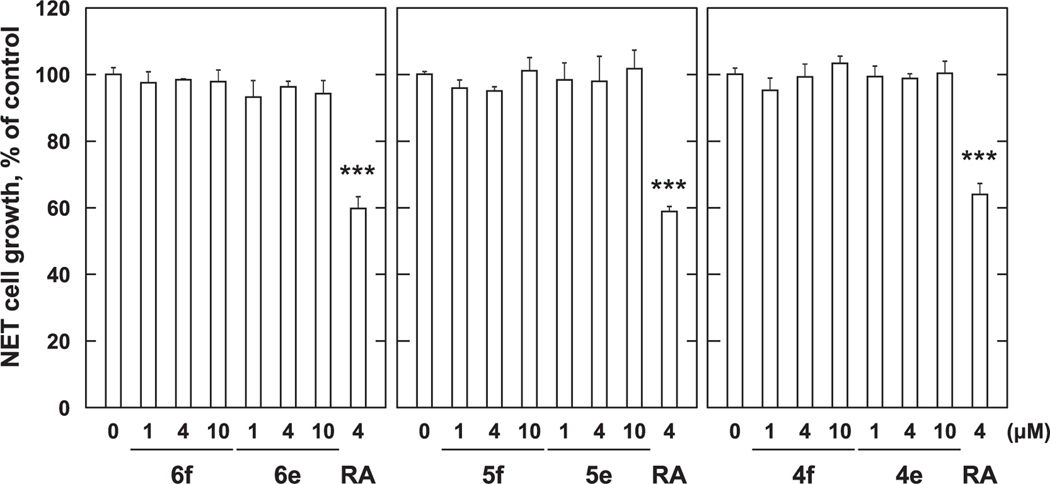
Growth inhibition with *p*-amidophenol and *p*-aminophenol derivatives against MCF-7 cells. MCF-7 cells (1 × 10^4^ cells/mL) were treated with various concentration of *p*-amidophenol and *p*-aminophenol derivatives for 72 h. Cell viability was determined by MTT method. Data shown are mean ± SD (*n* = 3). *p < 0.05, **p < 0.01, ***p < 0.001 vs Control (Dunnett’s multiple comparisons test).

**Fig. 8.**
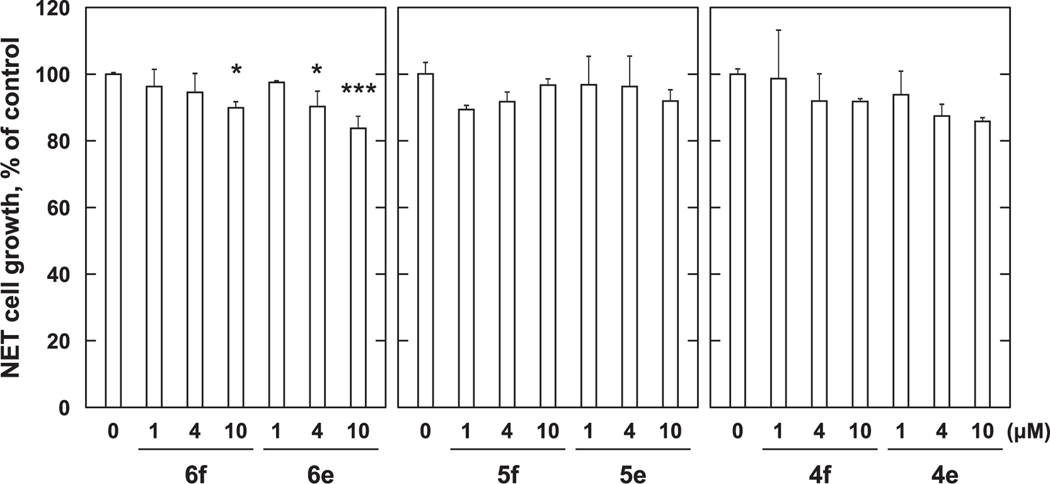
Growth inhibition with *p*-amidophenol and *p*-aminophenol derivatives against PC-3 cells. PC-3 cells (1 × 10^4^ cells/mL) were treated with various concentration of *p*-amidophenol and *p*-aminophenol derivatives for 72 h. Cell viability was determined by MTT method. Data shown are mean ± SD (*n* = 3). *p < 0.05, **p < 0.01, ***p < 0.001 vs Control (Dunnett’s multiple comparisons test).

Next, we examined the effects of converting the carboxyl-containing amidophenols **4b** – **6b** to their corresponding methyl esters (**4f** – **6f**, Figure 2). We examined compounds **4f**, **5f** and **6f** having chain-terminal carboxyl groups appended onto amidophenols **4b**, **5b** and **6b** and compounds **4e**, **5e** and **6e**, which represent variants of the *p*-alkylaminophenols **4a**, **5a** and **6a** having chain-terminal methyl esters. None of the methyl ester-containing analogs exhibited significant growth inhibitory potencies against MCF-7 cells (Fig. 7). In this assay, 4 μM RA inhibited MCF-7 cell growth by approximately 37%. The growth inhibitory effects were also minimal against PC-3 cells. While **6f** and **6e** showed minimal effects (approximately 10% inhibition), the growth inhibitory effects of **6b** and **6d** were abrogated by conversion to **6f** (Fig. 8). In this assay the growth inhibitory potency of 4 μM RA was approximately 20% (data not shown). This data indicates that the carboxyl group is important for the growth inhibitory effects of *p*-amidophenols (**4d**, **5d** and **6d**) but not for *p*-alkylaminophenols (**4c**, **5c** and **6c**). The data also suggest the methyl esters are not converted to the corresponding carboxylic acids within the cells.

### 3.4. Mechanisms of growth inhibition by 6d having a chain-terminal carboxyl group in human breast and prostate cancer cells

**Fig. 9.**
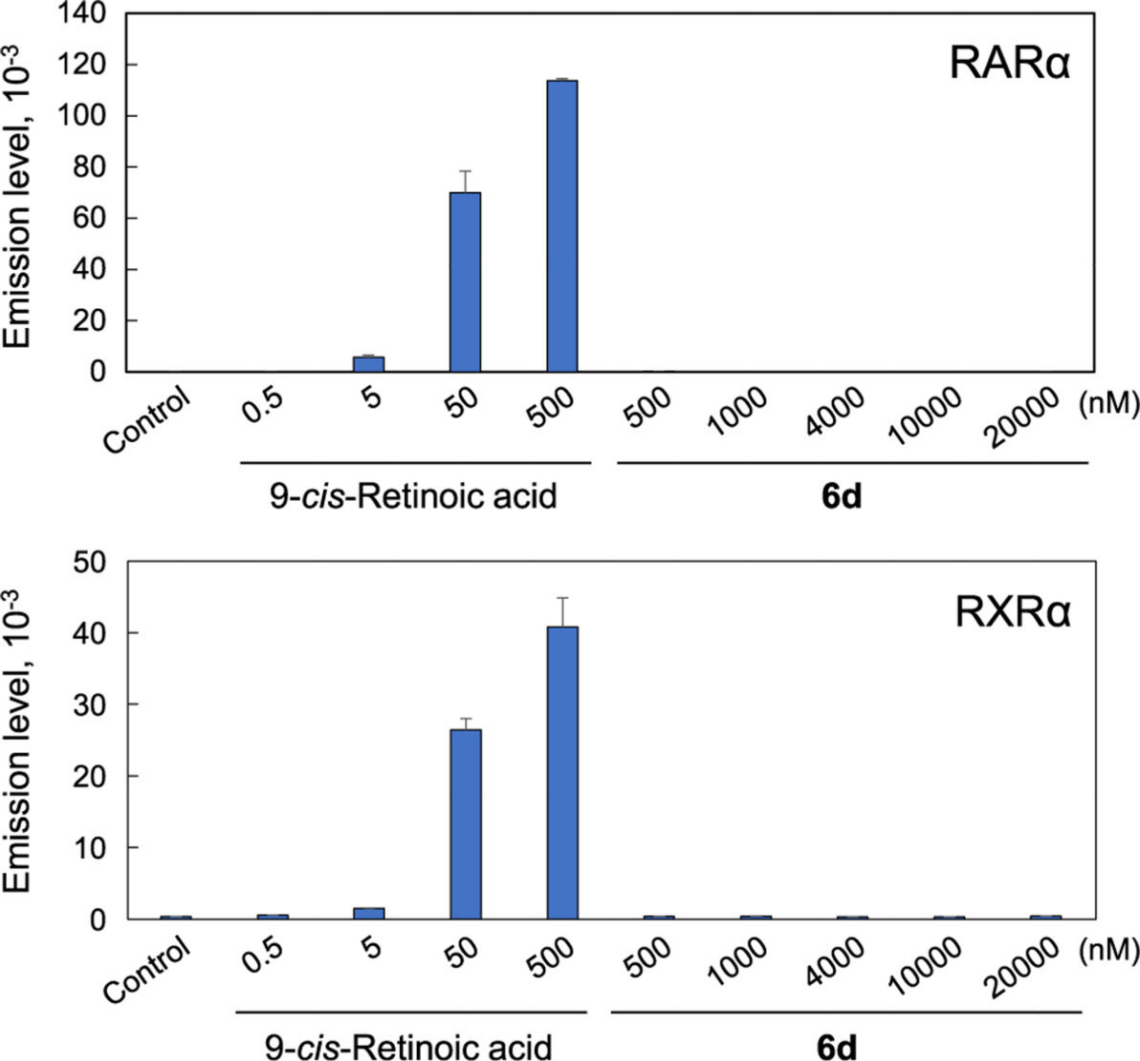
Transcriptional activity of **6d** and 9-*cis* RA. Cells were treated with 9-*cis* RA (0.5 ~ 500 nM) or **6d** (500 ~ 20000 nM), and luciferase activities were measured as described in ”Materials and Methods”. Each bar represents the mean ± SD (*n* = 3).

Compound **6d**, having a chain-terminal carboxyl group, showed enhanced growth inhibitory potencies against MCF-7 cells (Fig. 5) and PC-3 cells (Fig. 6). Previous reports have shown that RARα is involved in retinoid-mediated signaling and that it is important for the antiproliferative effects of RA in MCF-7 cells.^16^ In PC-3 cells, ligand binding to RARα and RXRα RA receptors has also been investigated.^17^ Therefore, in order to investigate whether the antiproliferative effects of **6d** against both cell lines are mediated by RAR or RXR or whether **6d** binds to RARα and RXRα and activates both receptors to exert its actions, we performed Luciferase-Reporter Assays on RARα and RXRα. In these assays, 9-*cis*-retinoic acid, an agonist of RARα and RXRα, was used as positive control. This increased luciferase activity in a concentration-dependent manner (0 ~ 500 nM), whereas **6d** had almost no effect at the control level (Fig. 9). These results suggest that **6d** did not bind to RARα and RXRα and that the cell growth inhibitory action of **6d** is not mediated by RARα and RXRα.

Since **6d** was found to exert its effects through mechanisms not mediated by the RA receptor, we investigated the effects of **6d** on other pathways (cell cycle arrest, induction of apoptosis (Sub-G1, BCL-2, BCL-xL), and phosphorylation inhibition of ERK). First, we examined the population of each cell cycle in **6d**-treated MCF-7 cells and PC-3 cells and the cell population of the Sub-G1 stage, which is an apoptosis index (data not shown). **6d** treatment on MCF-7 and PC-3 cells had no effect on the cell cycle and had no significant effect on the Sub-G1 population, an indicator of apoptosis. This data shows that **6d** does not induce cell cycle arrest. In addition, the effects of **6d** on expression of the apoptosis-inhibitory Bcl-2 and Bcl-xL proteins were investigated. The data showed that **6d** did not affect the expression of either protein. In addition, phosphorylated ERK levels involved in cell proliferation were not affected by **6d** treatment. These results suggest that **6d** does not induce apoptosis and inhibit ERK phosphorylation.

**Fig. 10.**
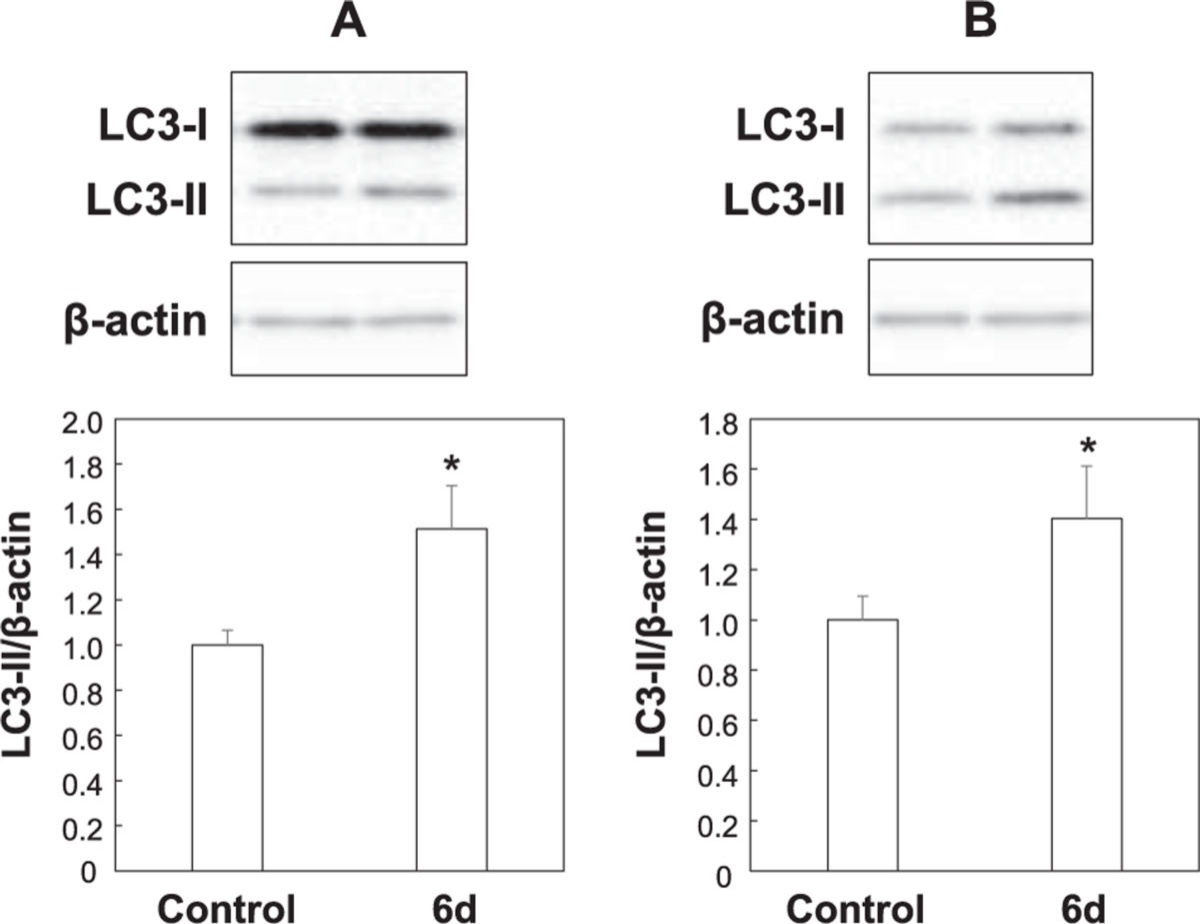
Protein levels of LC-3 in MCF-7 and PC-3 cells. MCF-7 (A) and PC-3 (B) cells (2 × 10^4^ cells/ml) were incubated at 37 °C in a humidified atmosphere of 5% CO_2_ in air. After 24 h, cells were treated with DMSO (control) or **6d** at 10 μM for 72 h. The protein levels of LC3 and β-actin were analyzed by Western blot analysis with specific antibodies against LC3 and β-actin as described in “Materials and Methods”. The value of control was defined as 1.0. Each bar represents the mean ± SD of each group (*n* = 3). *p < 0.05 compared with control.

**Fig. 11.**
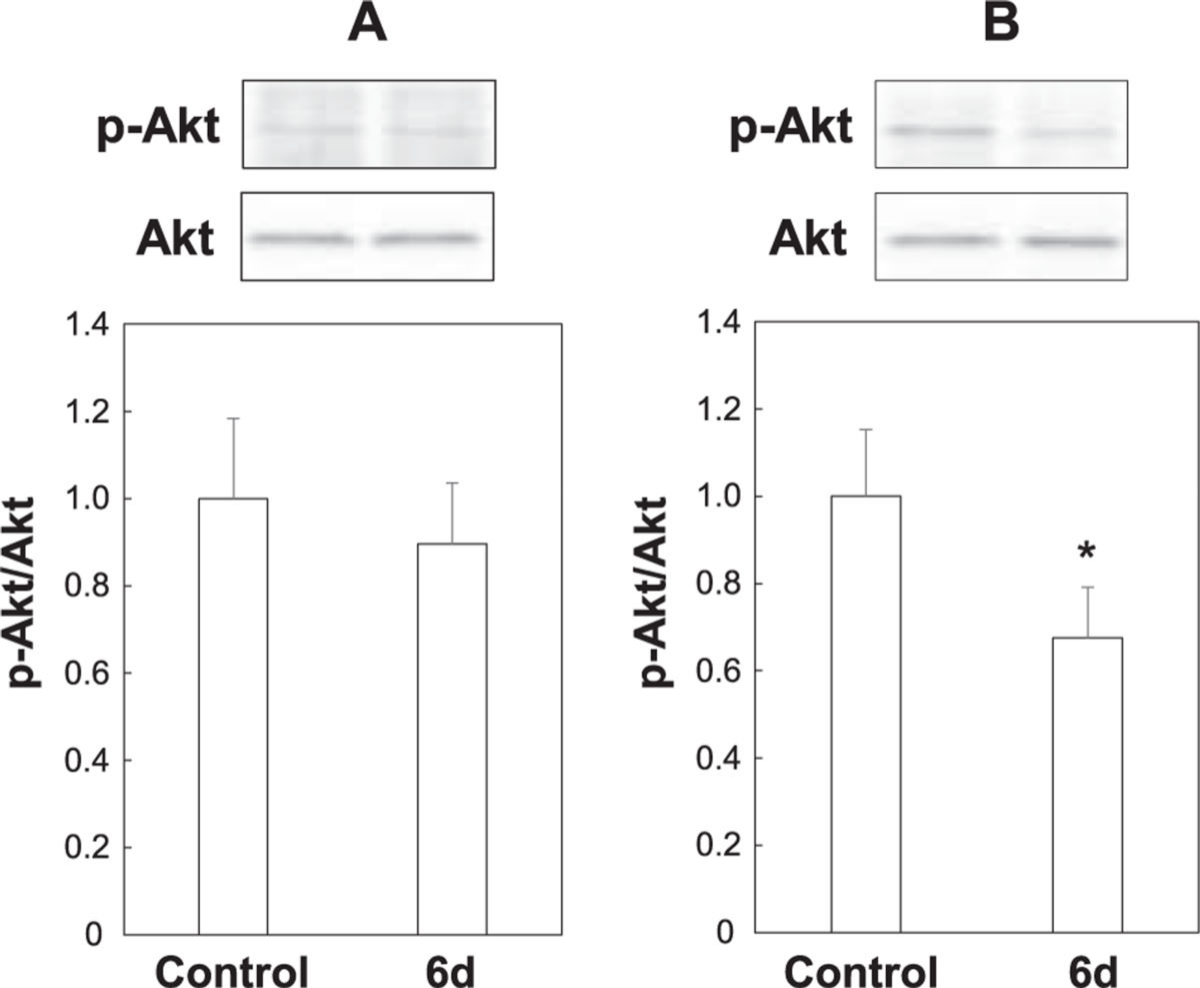
Protein levels of phosphorylated-Akt, and Akt in MCF-7 and PC-3 cells. MCF-7 (A) and PC-3 (B) cells (2 × 10^4^ cells/ml) were incubated at 37 °C in a humidified atmosphere of 5% CO_2_ in air. After 24 h, cells were treated with DMSO (control) or **6d** at 10 μM for 72 h. The protein levels of phosphorylated-Akt, and Akt were analyzed by Western blot analysis with specific antibodies against phosphorylated-Akt, and Akt as described in “Materials and Methods”. The value of control was defined as 1.0. Each bar represents the mean ± SD of each group (*n* = 3). *p < 0.05 compared with control.

Next, we examined the involvement of autophagy, one non-apoptotic cause of cell death. Treatment of MCF-7 and PC-3 cells with **6d** significantly increased by approximately 1.6-fold and 1.5-fold, respectively the expression of LC3-II (the lipid adduct of LC3-I), which is indicator of autophagy (Fig. 10). This suggests that **6d** induces autophagy in both cells. Finally, we investigated the effect of **6d** on the PI3K/Akt pathway, which is a survival signal. We found that phosphorylated Akt levels (p-Akt/Akt) decreased significantly by approximately 35% in PC-3 cells, while phosphorylated Akt levels tended to decrease in MCF-7 cells (Fig. 11). Collectively, this data suggests that **6d** suppresses the survival signal by Akt in PC-3 cells. Since no Sub-G1 population was observed and apoptosis was not induced, this suggests that **6d** suppresses the proliferation of MCF-7 and PC-3 cells by inducing autophagic cell death, which is non-apoptotic cell death.

## 4. Discussion

RA (**1**) is an active form of vitamin A that exhibits a variety of actions *in vivo*. It is used in the treatment of APL (acute promyelocytic leukemia) due to its effectiveness in inducing differentiation of leukemia cells. RA is believed to exert many of its actions by binding to RA receptors (RAR and RXR) and regulating gene expression. In addition, the terminal carboxylic acid group of RA has been reported to be important when RA binds to the RA receptors. To alleviate the side effects of retinoids and rexinoids, 4-HPR (**2**) was developed as an atypical retinoid. While 4-HPR has potent antiproliferative properties, it exhibits side effects, such as night blindness and ocular toxicity. 4-HPR represents a variant of RA in which the carboxylic acid group has been converted to an *p*-amidophenol group (Fig. 1). 4-HPR does not bind to RAR receptors, and it does not induce cell differentiation. The anti-proliferative activity of 4-HPR results from induction of apoptosis through the generation of ROS rather than through binding to RARs.^3^

In order to overcome the side effects of 4-HPR, a structure–activity relationship study was conducted that examined long-chain *N*-alkyl *p*-aminophenols.^8,13^ This resulted in the identification of *p*-DDAP (**3**) as having potent antiproliferative effects.^8–10^ In contrast, related long-chain *N*-alkyl *p*-amidophenols either lacked antiproliferative effects or showed extremely attenuated antiproliferative potentials.^8–10^ By analogy to RA, the focus of our current study was to examine the effects of introducing terminal carboxylic acid groups into long-chain *N*-alkyl *p*-alkylaminophenols. We found that modifying the parent *p*-alkylaminophenols (**4a** – **6a**) in this fashion (**4c** – **6c**) resulted in a loss of growth inhibitory potency against both MCF-7 cells (Fig. 3) and PC-3 cells (Fig. 4). In contrast, while against MCF-7 cells *p*-amidophenols (**4b** – **6b**) did not suppress proliferation (Fig. 5), the introduction of chain-terminal carboxyl groups onto the side chains of these *p*-amidophenols to yield **4d** – **6d**, enhanced antiproliferative potencies to values similar to what was found with RA. Against PC-3 cells *p*-amidophenols with shorter carbon chains inhibited cell growth more strongly (**4b** and **5b**, Fig. 6). The introduction of terminal carboxyl groups into the side chains of *p*-amidophenols enhanced the inhibitory effects on cell growth analogs having a longer chain (C12, **6d**), but attenuated the potencies of analogs having the shorter chains (C10 and C8, **5d** and **4d**). Against MCF-7 cells (Figure 7) and PC-3 cells (Fig. 8), the introduction of terminal carboxyl moieties into the side chains of *p*-alkylaminophenols resulted in a loss of effects on cell growth (**4e** – **6e**), relative to the corresponding analogs having free carboxylic acids (**4c** – **6c**). In contrast, conversion of the carboxylic acids in the amidophenol series (**4d** – **6d**) to their methyl esters (**4f** – **6f**) abolished the antiproliferative enhancement incurred by the original introduction of carboxylic acid functionality. The proliferation of MCF-7 cells was inhibited by approximately 60% by 4 μM RA, but that of PC-3 cells was inhibited by only approximately 20%. The *p*-alkylaminophenols and *p*-amidophenols were more effective than RA against PC-3 cells than to MCF-7. Introduction of carboxylic acid functionality enhanced antiproliferative potency against both cell lines with the exception of **5d** and **6d** against PC-3 cells.

While the introduction of carboxylic acid and methyl ester moieties into *p*-alkylaminophenols, which had originally been shown to have strong antiproliferative activities, significantly attenuated growth inhibitory effects, the introduction of carboxylic acid groups into the side chains of *p*-amidophenols, which had only a weak antiproliferative potencies, enhanced their potencies. However, the conversion of these acids to their corresponding methyl esters completely abolished the effects. While a carboxylic acid group is important for the binding of RA to the RA receptor, introduction of this functionality into *p*-alkylaminophenols abolishes antiproliferative potency, while enhancing the potency of *p*-amidophenols. Conversion of the carboxylic acids to their methyl esters completely abolished antiproliferative potency, in spite of the fact that this should have increased lipophilicity and cellular uptake. This suggests that the methyl esters are not converted to their active free acids by esterases in the cytoplasm.

Compound **6a** is a highly promising compound that is non-toxic and non-inflammatory when applied to the skin of hairless mice *in vivo*.^18^ In contrast, RA induces inflammation when applied to hairless mice. In addition, **6a** is non-toxic to the non-malignant human epidermal keratinocyte cell line (HaCaT cells) *in vitro*. Compound **6a**, which has a phenolic OH residue, undergoes metabolism by mechanisms that include glucuronidation and sulfate conjugation and it is excreted in the urine. Compound **6a** is also considered to be non-carcinogenic because it does not decompose into primary amines. When administered *in vivo*, 4-HPR (**2**) an aminophenol derivative of RA, whose clinical trial was discontinued due to side effects (impaired dark adaptation), decreases blood retinol levels, whereas **6a** does not reduced retinol levels. Compound **6a** eliminates the side effect of impaired dark adaptation, because it has no effect on blood retinol levels.^9^ No cytotoxic effects of **6a** were observed in *in vivo* studies using prostate cancer cells and neuroblastoma.^9,11^

Compound **6a** was developed based on the speculation that the cyclohexene ring is the cause of the side effects of 4-HPR. The structure of 4-HPR was divided into four parts and structure–activity relationship studies were conducted. These studies determined that the methylaminophenol moiety played a central role and that extending the length of the polymethylene side chain was beneficial.^13,7^ The phenolic OH moiety is important for the cancer cell growth inhibitory action of compound **6a**, and the action is enhanced by elongating the side chain of methylaminophenol.^9^ It has been reported that **6a** does not bind to RARs and RXRs,^18^ and that growth inhibitory effects on prostate cancer PC-3 cells are due to induction of apoptosis and cell cycle arrest.^9^ In our current study, we investigated the mechanism of action of the newly developed **6d** and found that **6d** does not bind to RARα and RXRα (Fig. 9), arrest cell cycle, induce apoptosis or inhibit the ERK pathway. In contrast we found that **6d** induces autophagy and inhibits the Akt pathway, leading to non-apoptotic cell death. These results suggest that the growth suppression of MCF-7 cells and PC-3 cells by **6a** and **6d** are not mediated by RA receptors and that they have different mechanisms of action.

Our previous studies have examined the inhibitory effects of cinnamic acid derivatives, including caffeic acid phenethyl ester, on the growth of MCF-7 and PC-3 cells.^19^ The inhibitory effects on cancer cell growth are stronger depending on the number of OH groups on the cinnamic acid aryl ring and that tri-hydroxycinnamic acid is more potent than the mono- and di-hydroxycinnamic acids. These results indicate that the growth inhibitory effects on cancer cells becomes stronger with increasing number of hydroxyl groups on the cinnamic acid aryl ring. Our current study shows that the anti-cancer effects of **6a** and **6d** have anti-proliferative potencies against MCF-7 and PC-3 cells. These results suggests the potential value of investigating the effects on anti-proliferative potencies of increasing the number of OH groups on the aromatic ring.

In conclusion, we have shown that introduction of chain-terminal carboxylic acid moieties into a series of *p*-alkylamidophenols enhances growth inhibitory potencies against both MCF-7 and PC-3 cells. The phenolic 4-carboxyamido group was seen as being important for this potency enhancement, since the effect was not observed when similar carboxylic acid functionality was introduced into a corresponding series of *p*-alkylaminophenols. This may reflect a difference between MCF-7 cells and PC-3 cells in the molecular targets being affected by RA, *p*-alkylaminophenols and *p*-alkylamidophenols.

## Supplementary Material

MMC1
