# Supplementary material for: Examination of aminophenol-containing compounds designed as antiproliferative agents and potential atypical retinoids
Source: Bioorg Med Chem. Author manuscript; Available in PMC 2023 Apr 17. (PMC10107075; doi:10.1016/j.bmc.2023.117214)
Supplement: MMC1 [file NIHMS1882658-supplement-MMC1.pdf]

Supporting Information

Examination of Aminophenol-Containing Compounds Designed as Antiproliferative Agents and Potential Atypical Retinoids

Ramesh M. Chingle,[1,†] Masahiko Imai,[2,†] Sarah Altman,[1] Daisuke Saito,[2] Noriko Takahashi[2,*] and Terrence R. Burke, Jr.,[1,*]

[1]Chemical Biology Laboratory, Center for Cancer Research, National Cancer Institute, National Institutes of Health, Frederick, MD 21702, USA

[2]Laboratory of Physiological Chemistry, Institute of Medicinal Chemistry, Hoshi University, 2-4-41 Ebara, Shinagawa, Tokyo 142-8501, Japan

[†]Equal contributions

*Correspondence

Noriko Takahashi; E-mail: t-noriko@hoshi.ac.jp

Terrence Burke; E-mail: burkete@nih.gov

NMR Spectra

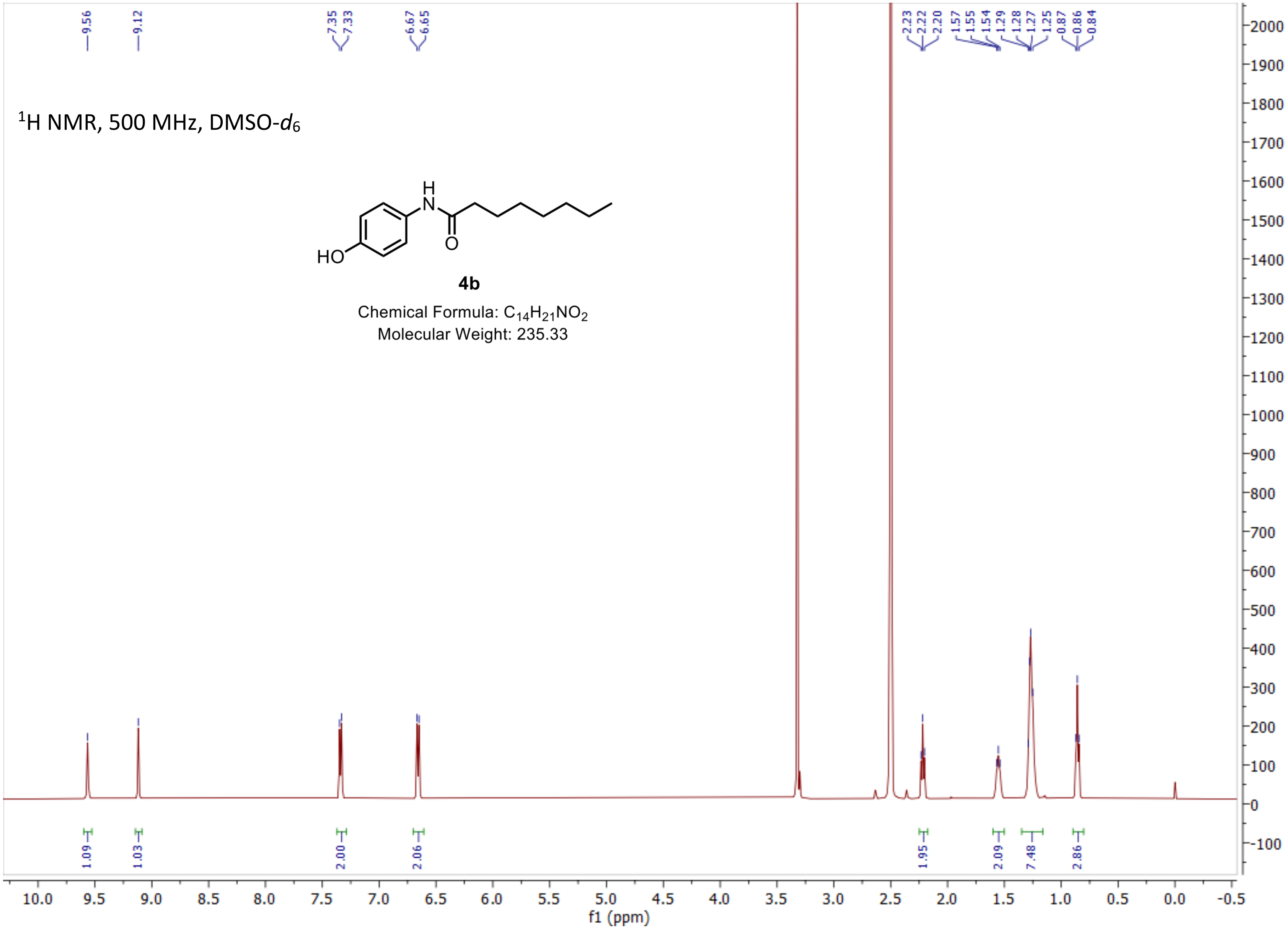

^{1}H NMR, 500 MHz, DMSO-d_6
HO
H
N
O
4b
Chemical Formula: $C_{14}H_{21}NO_2$
Molecular Weight: 235.33
9.56
9.12
7.35
7.33
6.67
6.65
2.23
2.22
2.20
1.57
1.55
1.54
1.29
1.28
1.27
1.25
0.87
0.86
0.84
1.09
1.03
2.00
2.06
1.95
2.09
7.48
2.86
f1 (ppm)

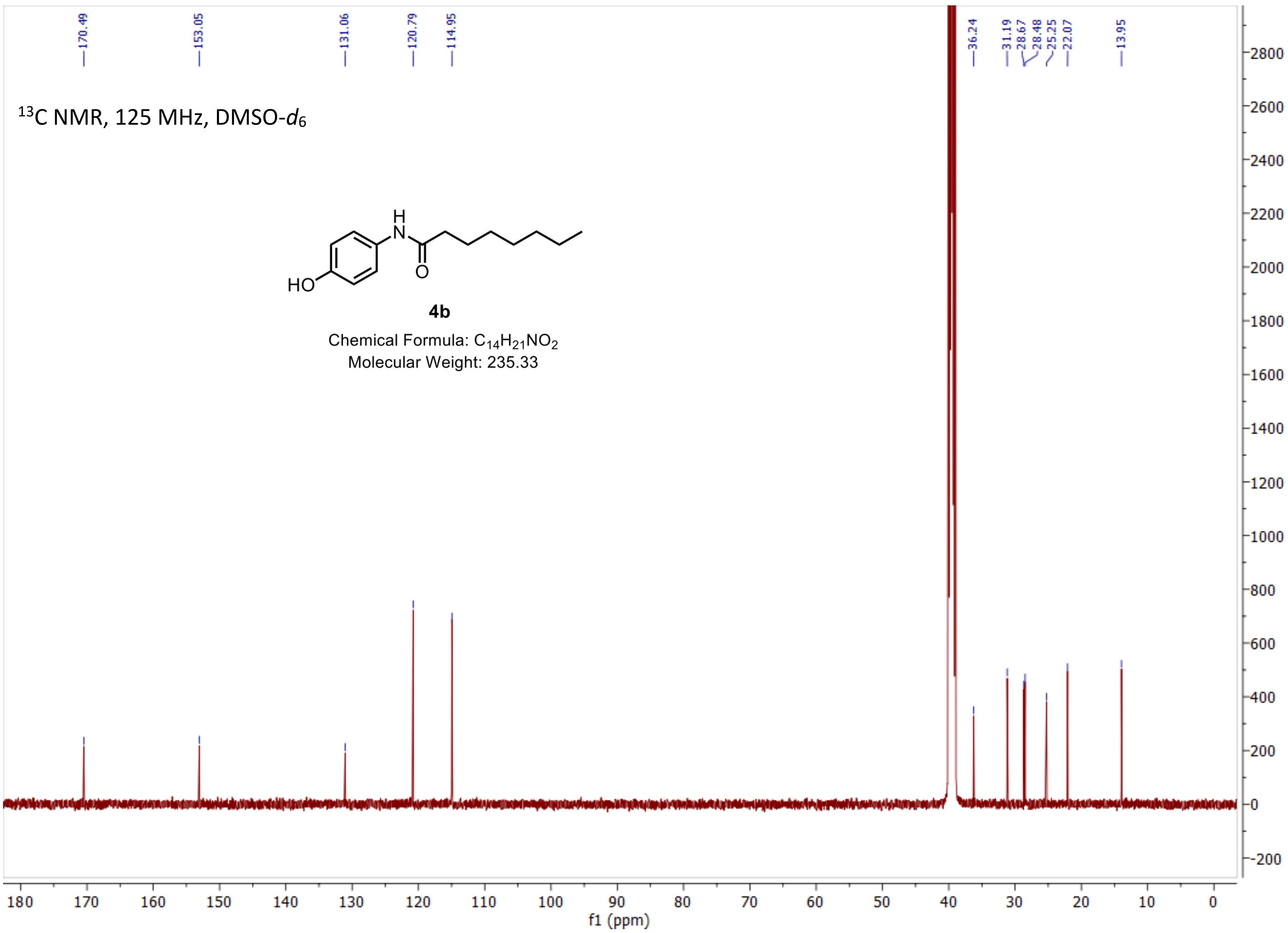

170.49
153.05
131.06
120.79
114.95
36.24
31.19
28.67
28.48
25.25
22.07
13.95
^{13}C NMR, 125 MHz, DMSO-d_6
H
N
O
HO
4b
Chemical Formula: $C_{14}H_{21}NO_2$
Molecular Weight: 235.33
f1 (ppm)

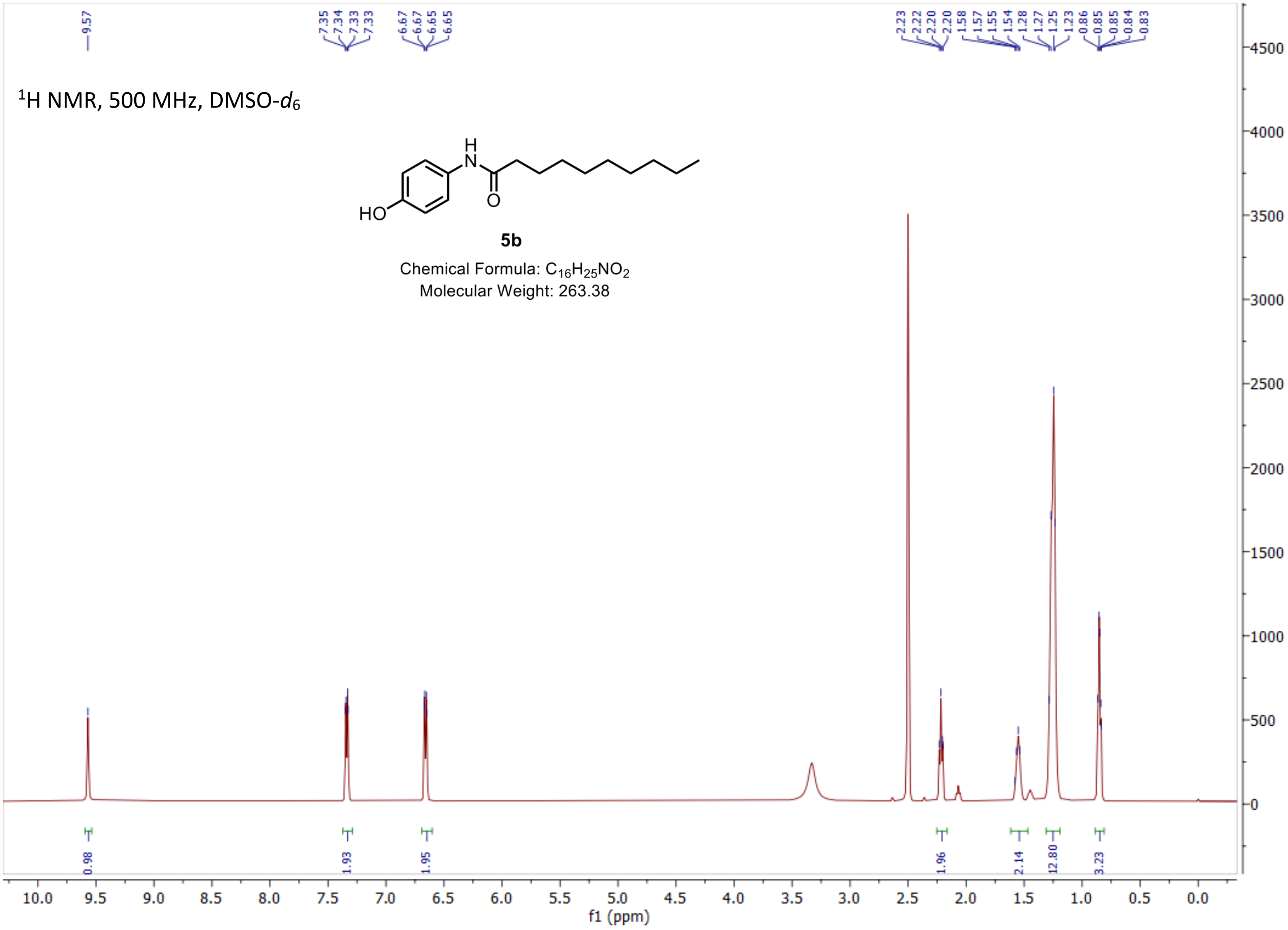

¹H NMR, 500 MHz, DMSO-d6
HO
H
N
O
5b
Chemical Formula: C16H25NO2
Molecular Weight: 263.38
9.57
7.35 7.34 7.33 7.33
6.67 6.67 6.65 6.65
2.23 2.22 2.20 2.20 1.58 1.57 1.55 1.54 1.28 1.27 1.25 1.23 0.86 0.85 0.85 0.84 0.83
0.98 1.93 1.95 1.96 2.14 12.80 3.23
4500 4000 3500 3000 2500 2000 1500 1000 500 0
10.0 9.5 9.0 8.5 8.0 7.5 7.0 6.5 6.0 5.5 5.0 4.5 4.0 3.5 3.0 2.5 2.0 1.5 1.0 0.5 0.0
f1 (ppm)

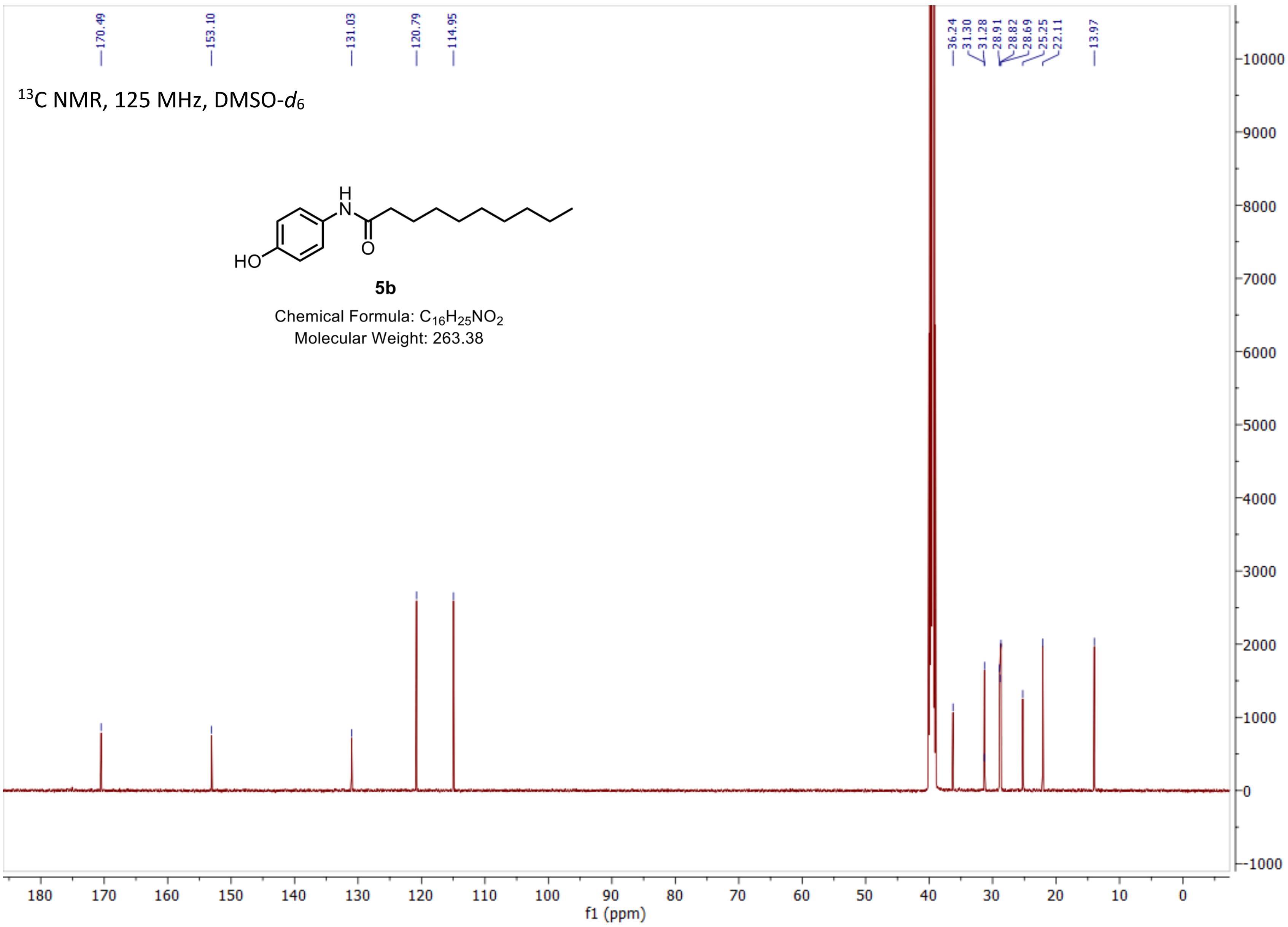

170.49
153.10
131.03
120.79
114.95
36.24
31.30
31.28
28.91
28.82
28.69
25.25
22.11
13.97
^{13}C NMR, 125 MHz, DMSO-d_6
H
N
O
HO
5b
Chemical Formula: $C_{16}H_{25}NO_2$
Molecular Weight: 263.38
f1 (ppm)

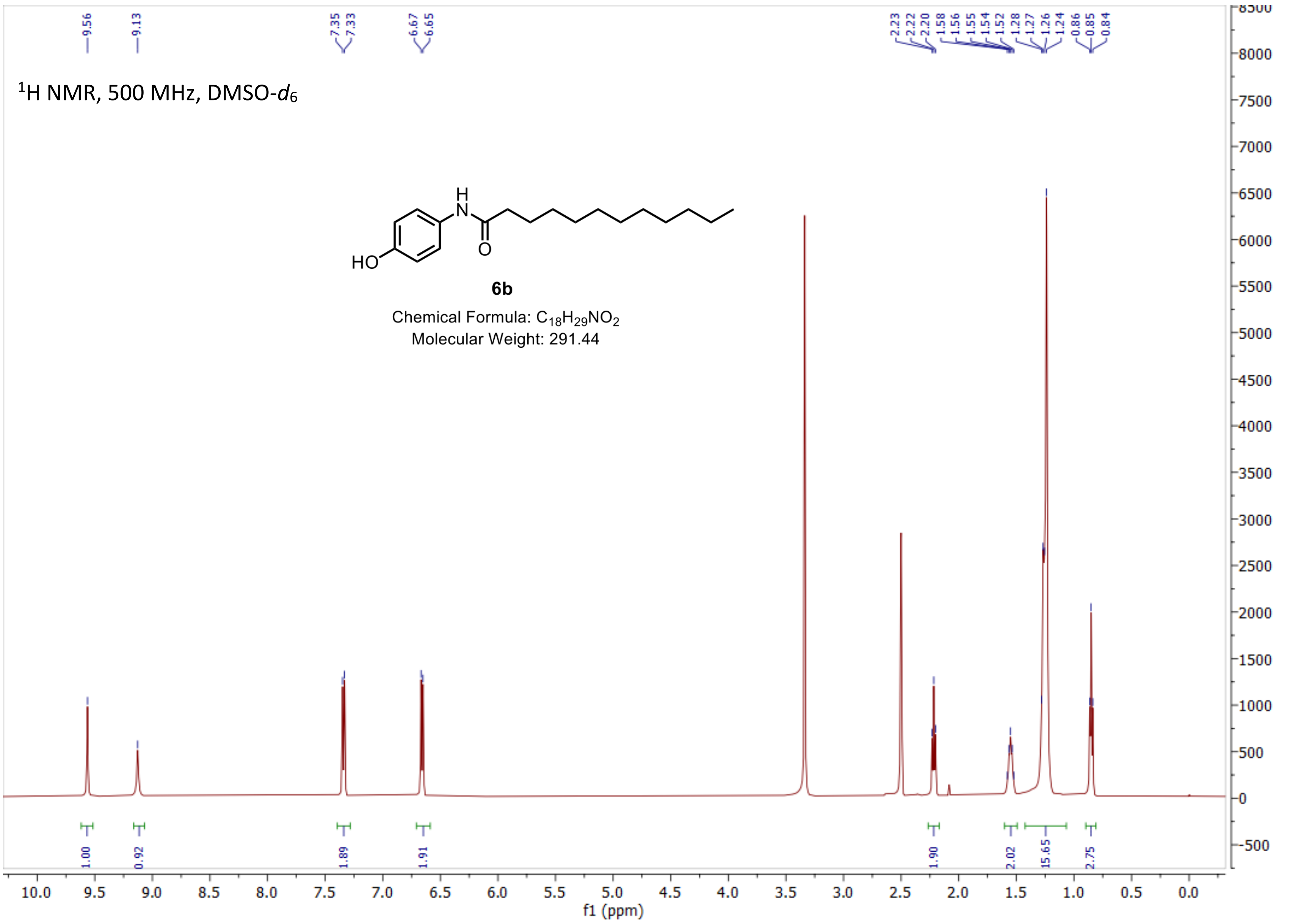

9.56
9.13
7.35
7.33
6.67
6.65
2.23
2.22
2.20
1.58
1.56
1.55
1.54
1.52
1.28
1.27
1.26
1.24
0.86
0.85
0.84
¹H NMR, 500 MHz, DMSO-d_6
HO
H
N
O
6b
Chemical Formula: $C_{18}H_{29}NO_2$
Molecular Weight: 291.44
1.00
0.92
1.89
1.91
1.90
2.02
15.65
2.75
10.0 9.5 9.0 8.5 8.0 7.5 7.0 6.5 6.0 5.5 5.0 4.5 4.0 3.5 3.0 2.5 2.0 1.5 1.0 0.5 0.0
f1 (ppm)
8500 8000 7500 7000 6500 6000 5500 5000 4500 4000 3500 3000 2500 2000 1500 1000 500 0 -500

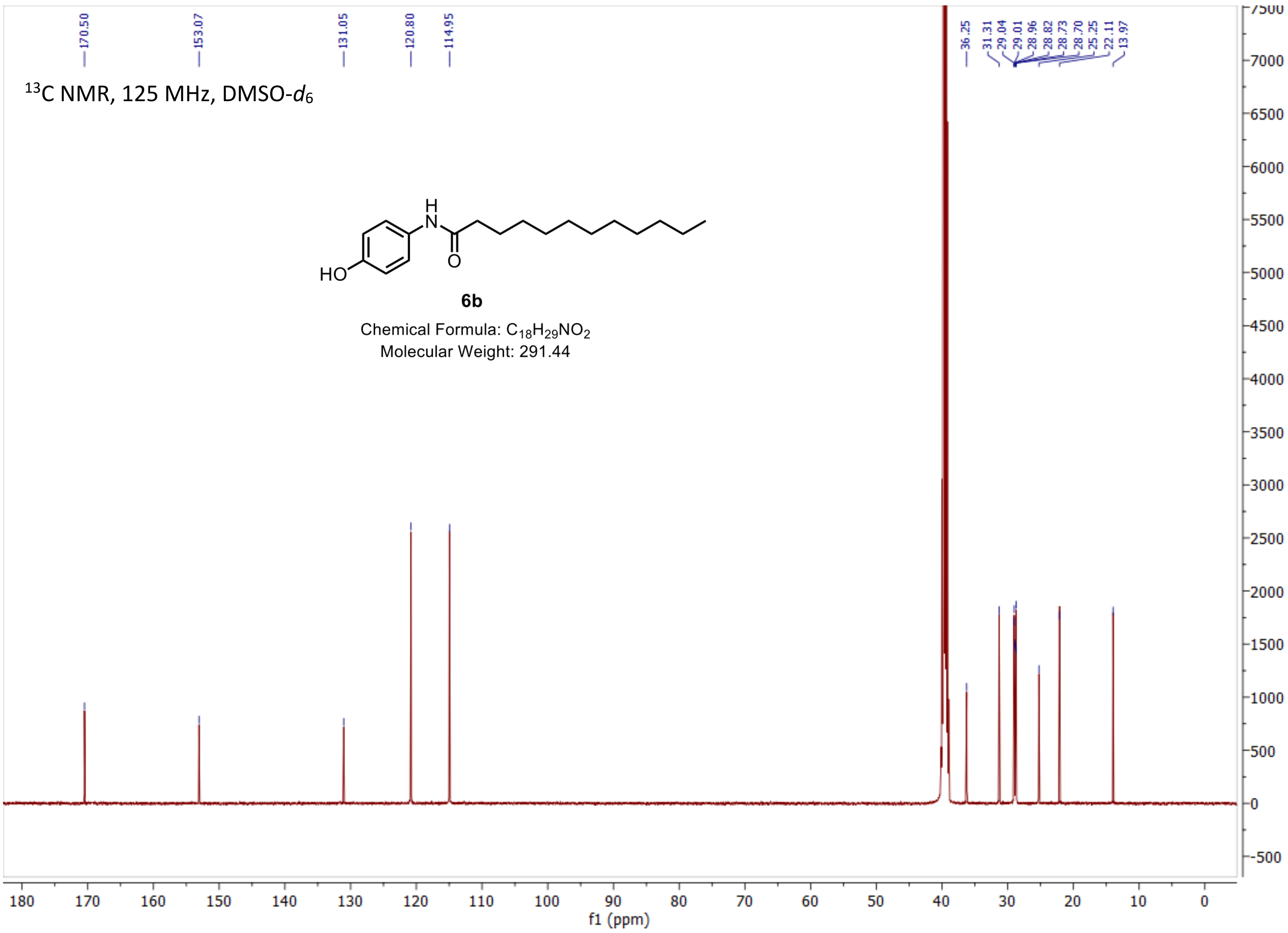

13C NMR, 125 MHz, DMSO-d6
170.50
153.07
131.05
120.80
114.95
36.25
31.31
29.04
29.01
28.96
28.82
28.73
28.70
25.25
22.11
13.97
HO
H
N
O
6b
Chemical Formula: C18H29NO2
Molecular Weight: 291.44
f1 (ppm)

^{1}H NMR, 500 MHz, DMSO-d_6

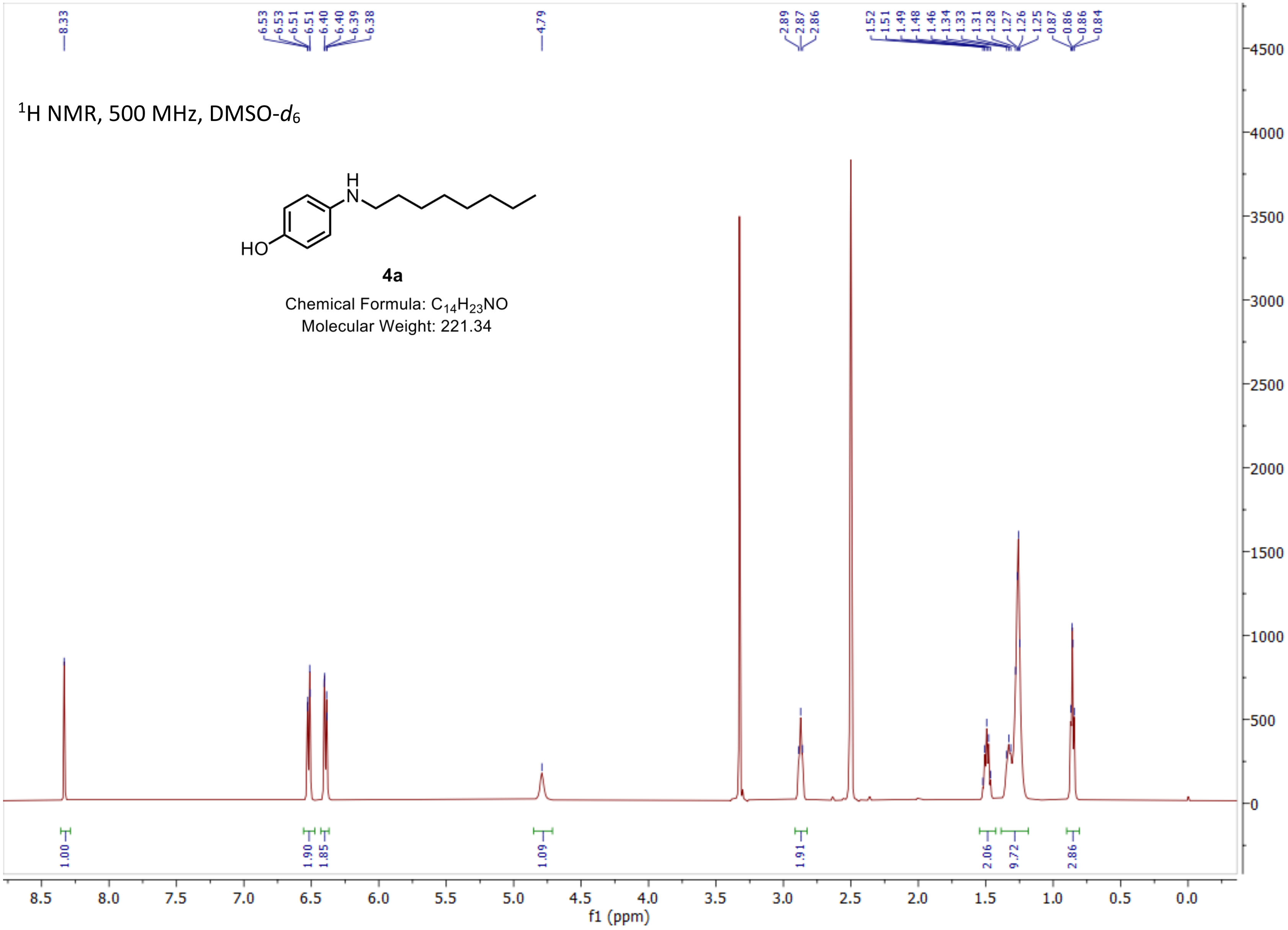

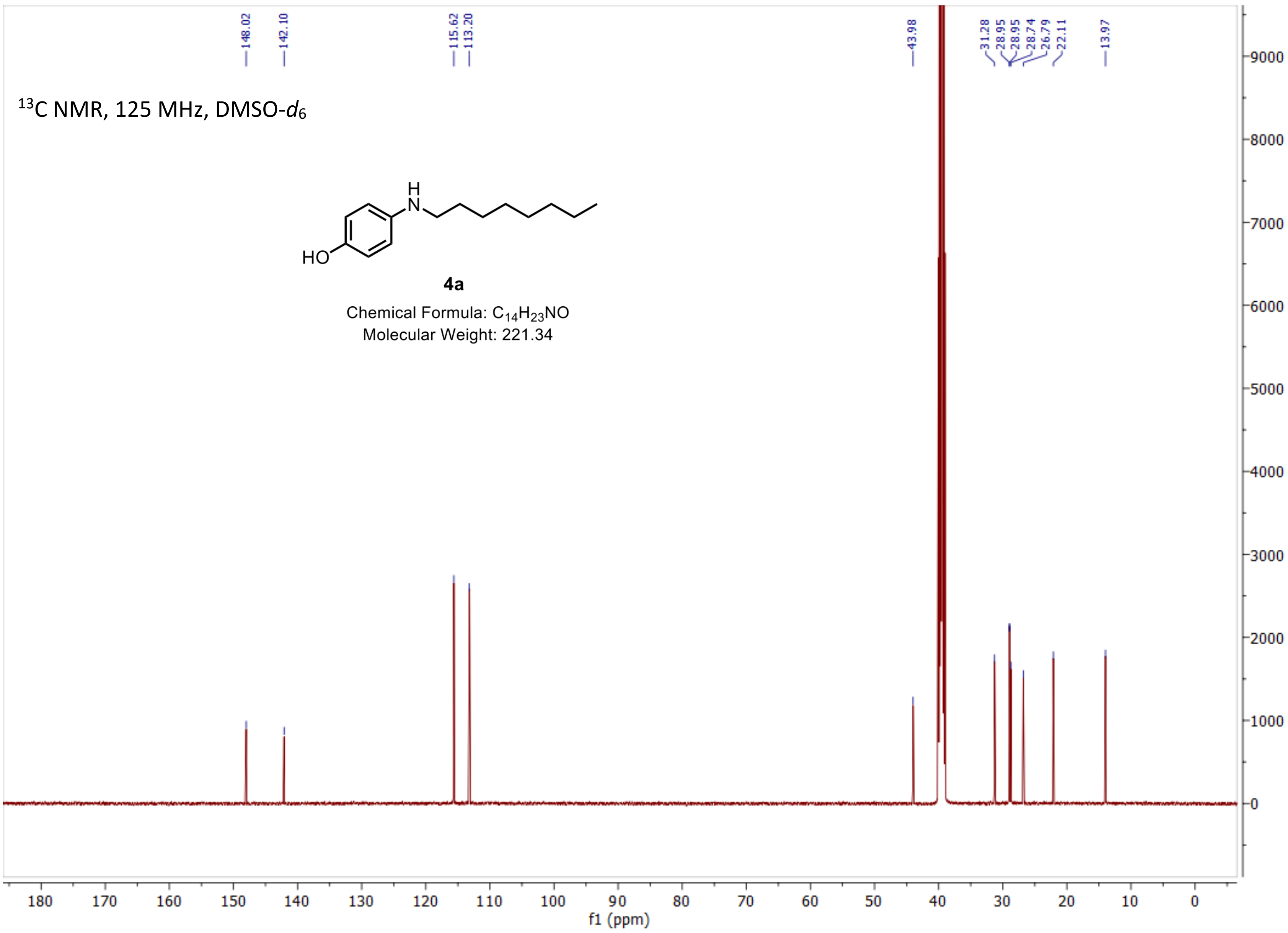

13C NMR, 125 MHz, DMSO-d6
HO
H
N
4a
Chemical Formula: C14H23NO
Molecular Weight: 221.34
148.02
142.10
115.62
113.20
43.98
31.28
28.95
28.95
28.74
26.79
22.11
13.97
9000
8000
7000
6000
5000
4000
3000
2000
1000
0
180 170 160 150 140 130 120 110 100 90 80 70 60 50 40 30 20 10 0
f1 (ppm)

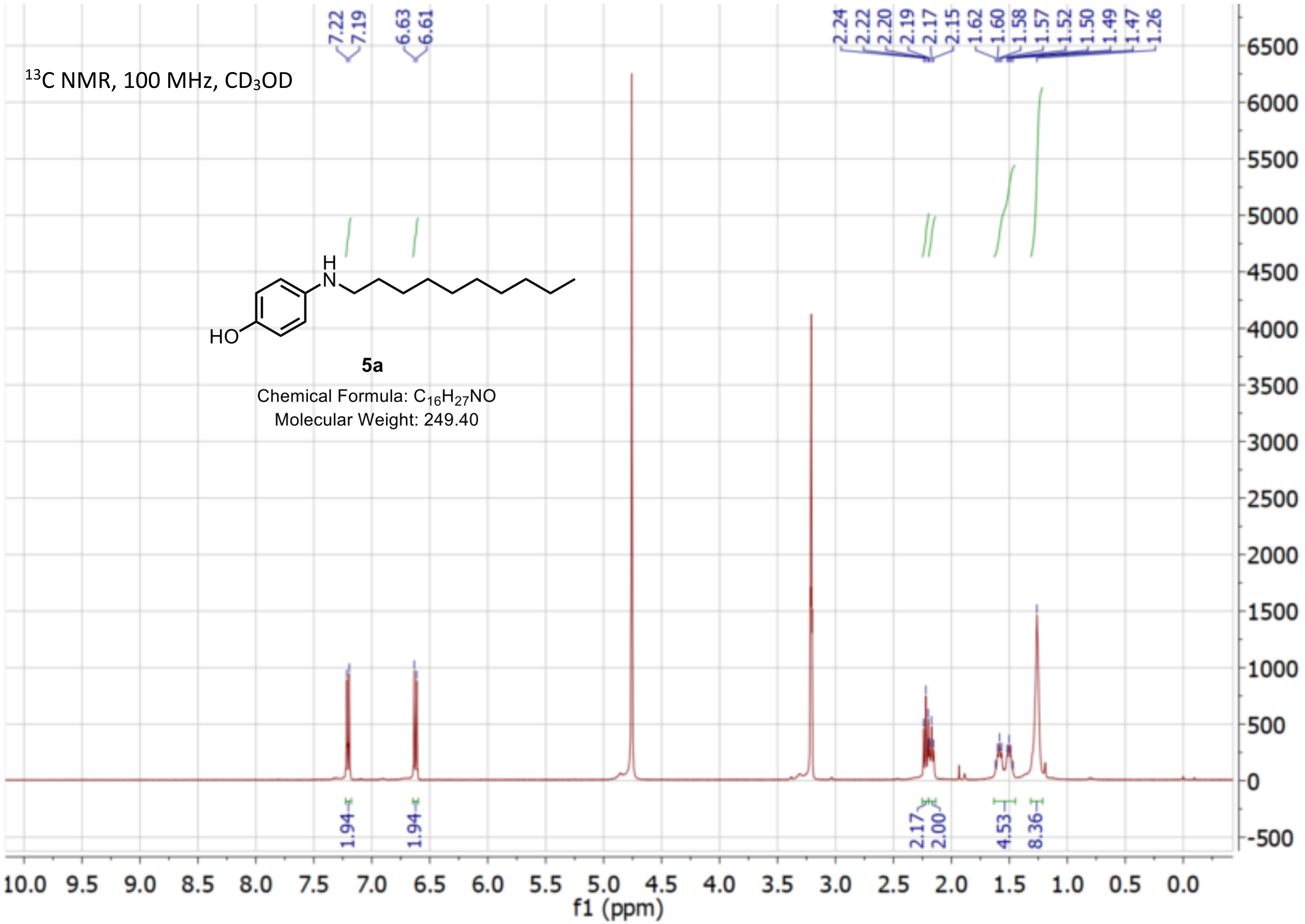

13C NMR, 100 MHz, CD3OD
7.22
7.19
6.63
6.61
2.24
2.22
2.20
2.19
2.17
2.15
1.62
1.60
1.58
1.57
1.52
1.50
1.49
1.47
1.26
H
N
HO
5a
Chemical Formula: C16H27NO
Molecular Weight: 249.40
1.94
1.94
2.17
2.00
4.53
8.36
f1 (ppm)

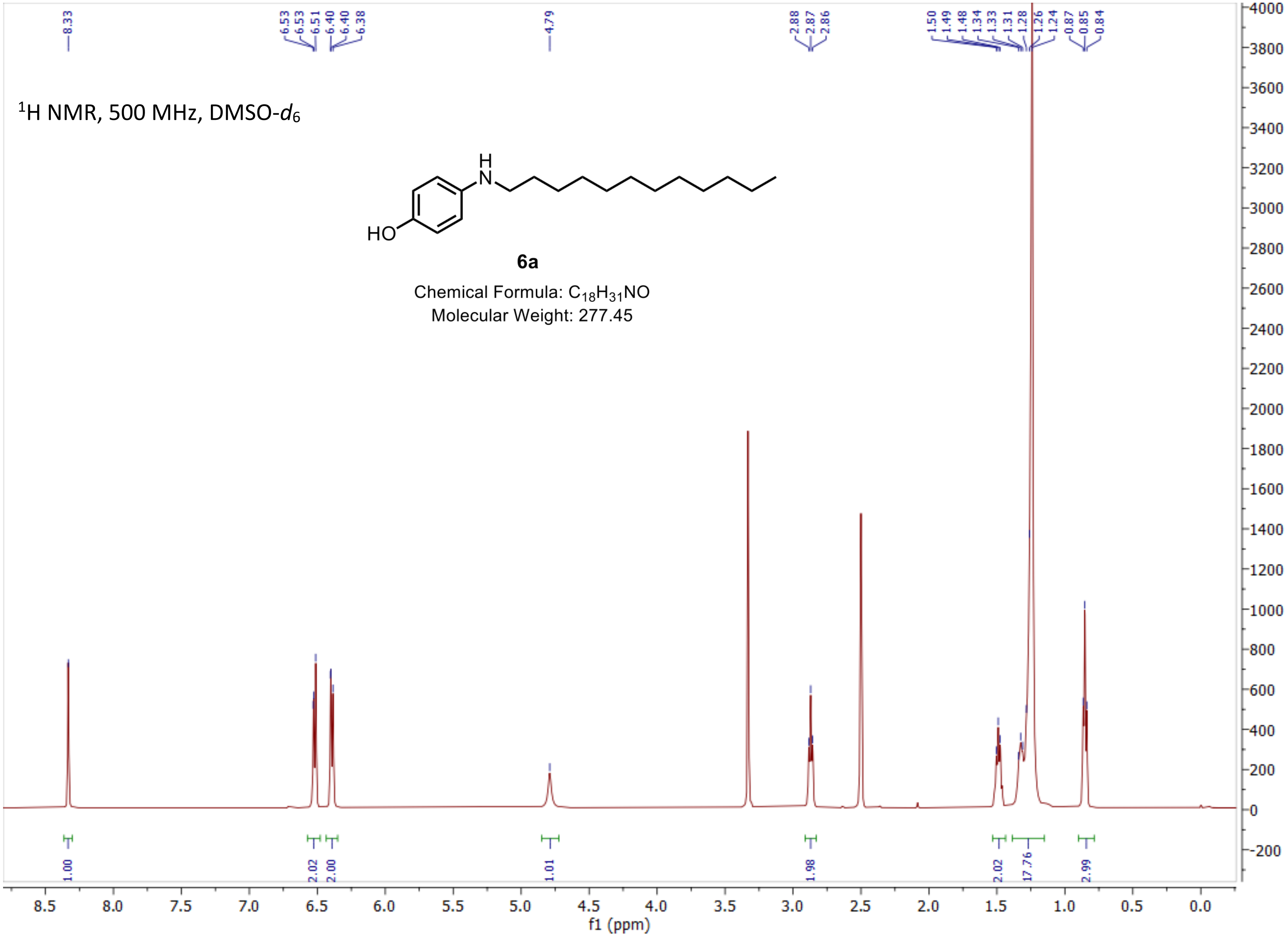

8.33
6.53
6.53
6.51
6.40
6.40
6.38
4.79
2.88
2.87
2.86
1.50
1.49
1.48
1.34
1.33
1.31
1.28
1.26
1.24
0.87
0.85
0.84
1H NMR, 500 MHz, DMSO-d6
H
N
HO
6a
Chemical Formula: C18H31NO
Molecular Weight: 277.45
1.00
2.02
2.00
1.01
1.98
2.02
17.76
2.99
f1 (ppm)

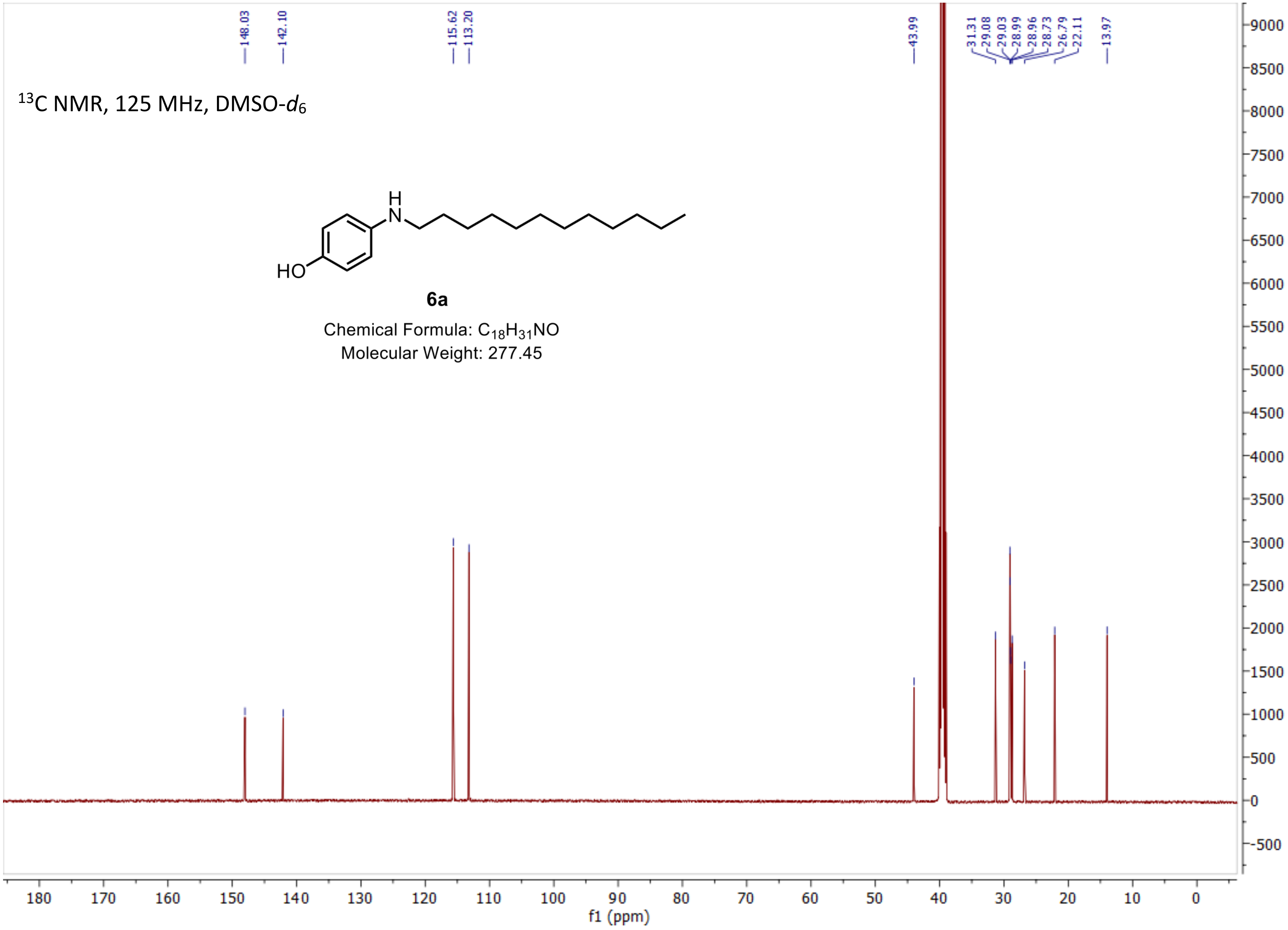
13C NMR, 125 MHz, DMSO-d6
148.03
142.10
115.62
113.20
43.99
31.31
29.08
29.03
28.99
28.96
28.73
26.79
22.11
13.97
H
N
HO
6a
Chemical Formula: C18H31NO
Molecular Weight: 277.45
f1 (ppm)

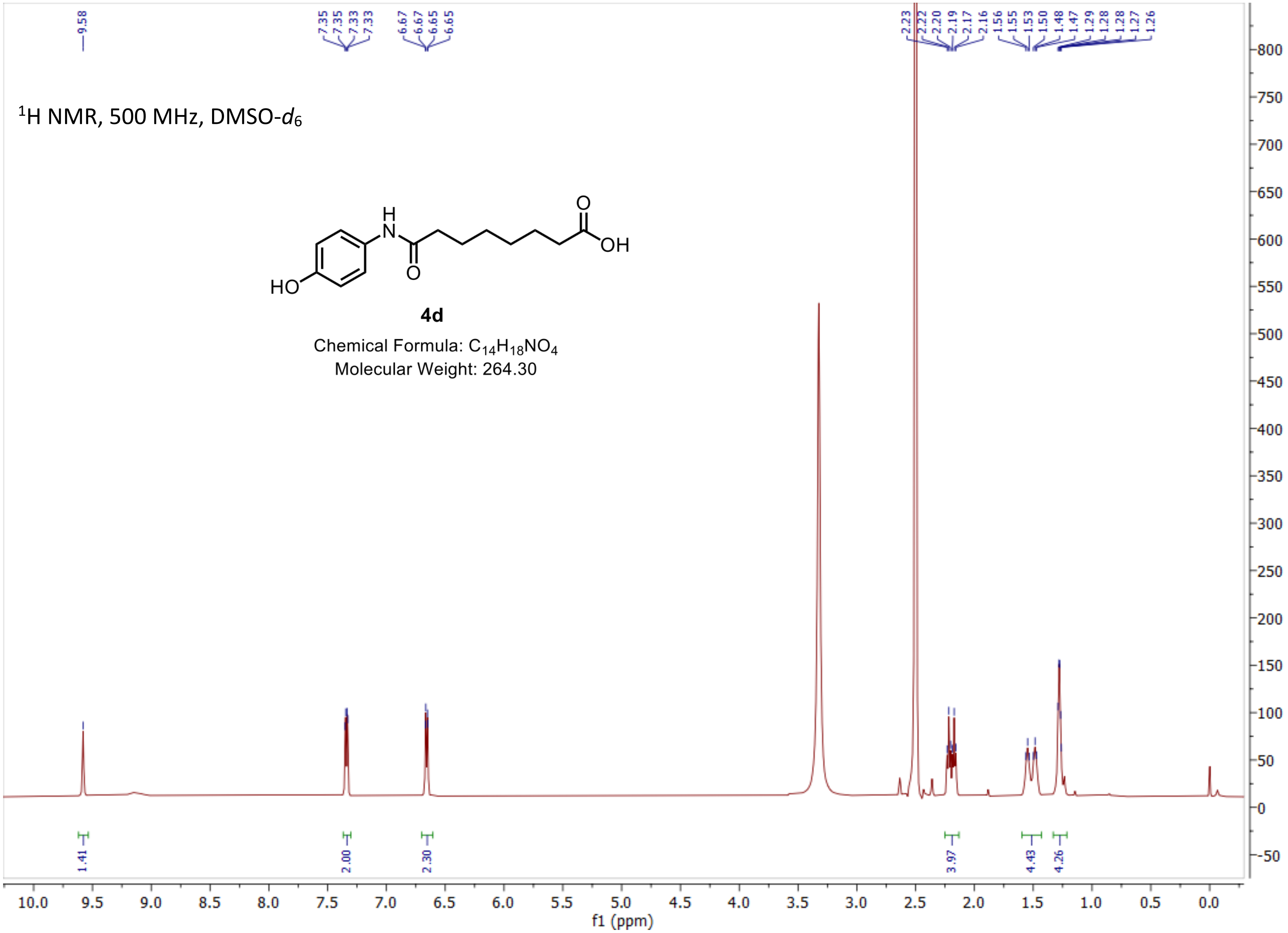

1H NMR, 500 MHz, DMSO-d6
HO
H
N
O
O
OH
4d
Chemical Formula: C14H18NO4
Molecular Weight: 264.30
f1 (ppm)

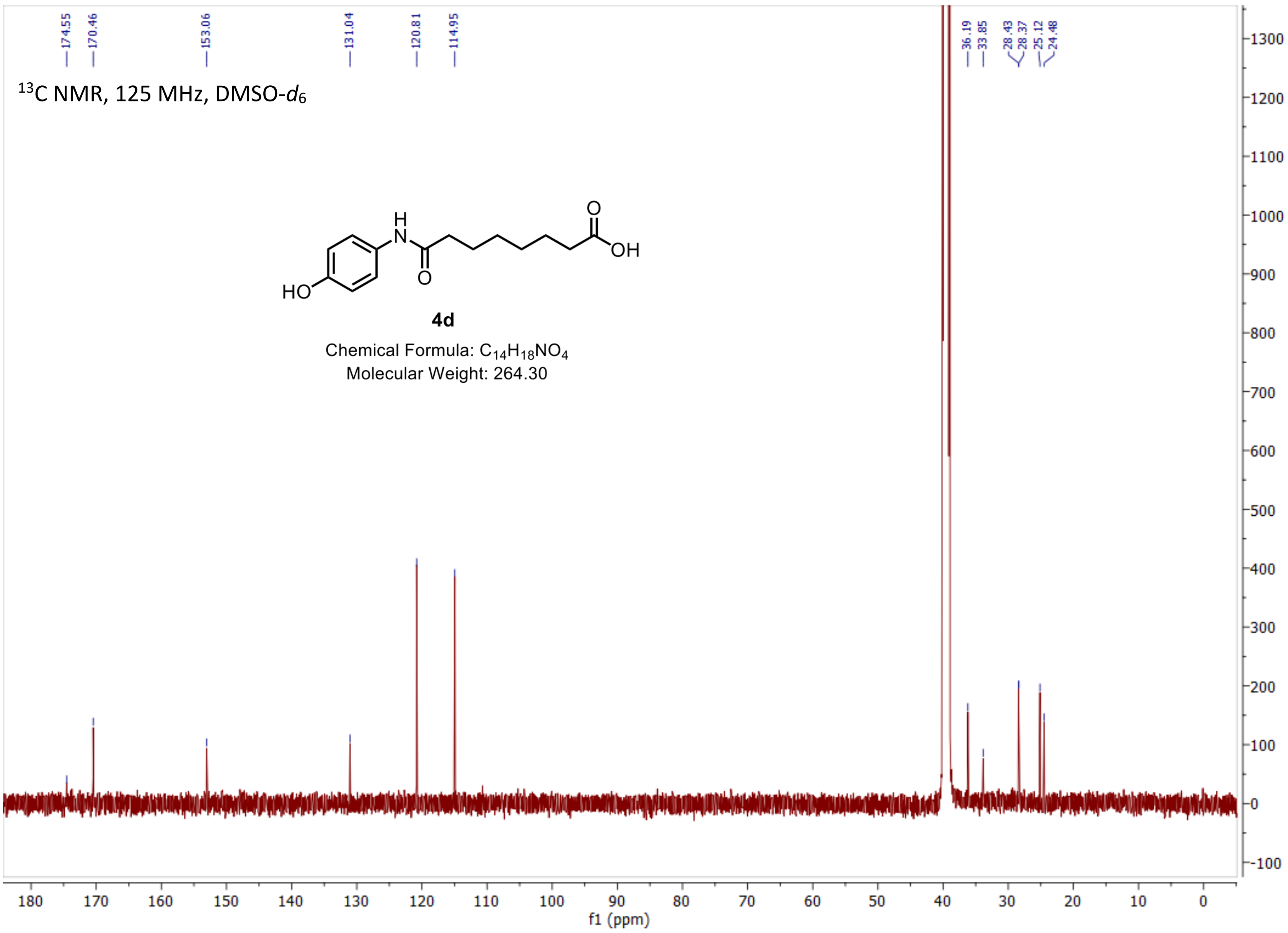
174.55
170.46
153.06
131.04
120.81
114.95
36.19
33.85
28.43
28.37
25.12
24.48
13C NMR, 125 MHz, DMSO-d6
HO
H
N
O
O
OH
4d
Chemical Formula: C14H18NO4
Molecular Weight: 264.30
f1 (ppm)

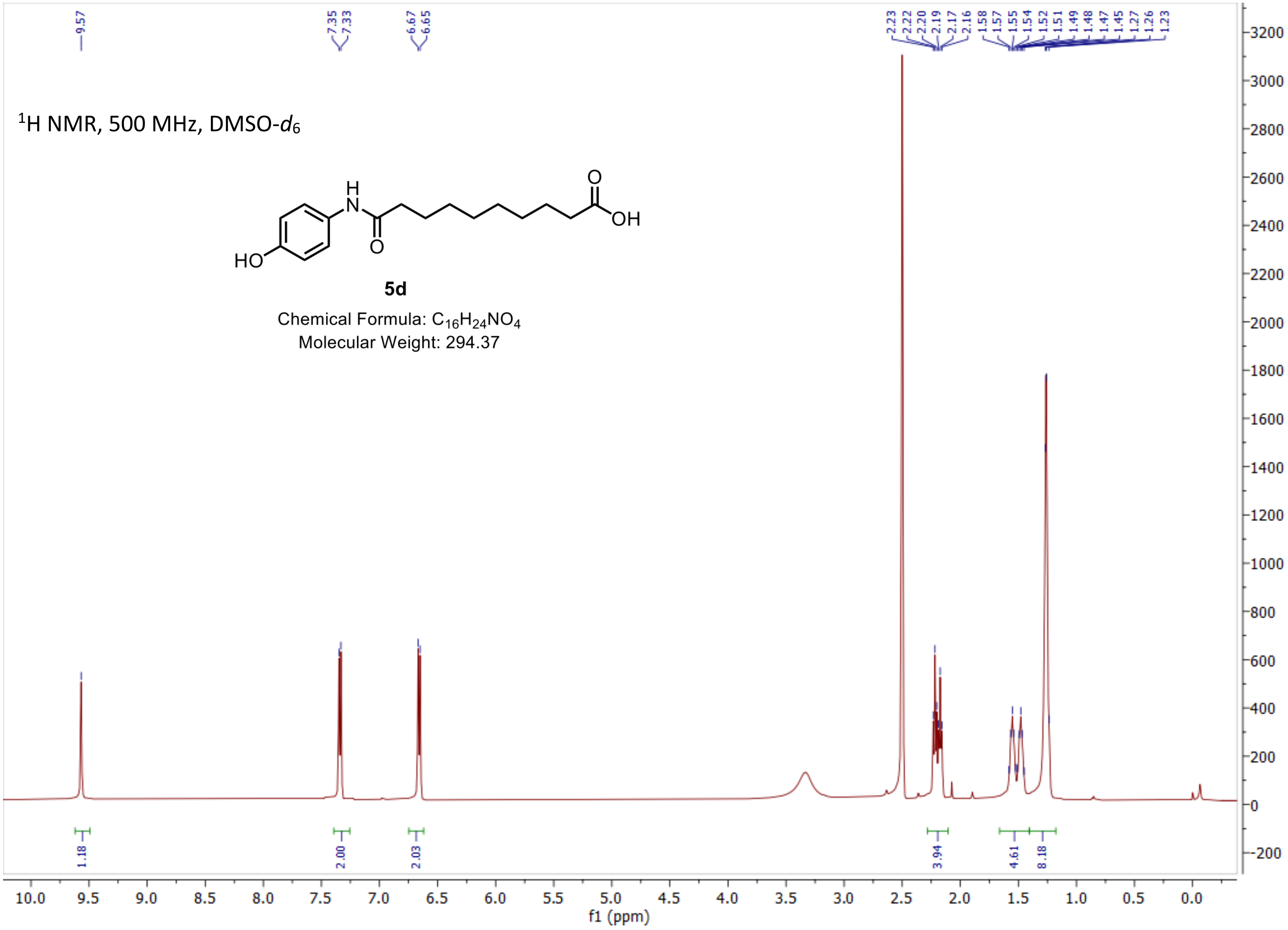

1H NMR, 500 MHz, DMSO-d6
HO
H
N
O
O
OH
5d
Chemical Formula: C16H24NO4
Molecular Weight: 294.37
9.57
7.35
7.33
6.67
6.65
2.23
2.22
2.20
2.19
2.17
2.16
1.58
1.57
1.55
1.54
1.52
1.51
1.49
1.48
1.47
1.45
1.27
1.26
1.23
1.18
2.00
2.03
3.94
4.61
8.18
f1 (ppm)

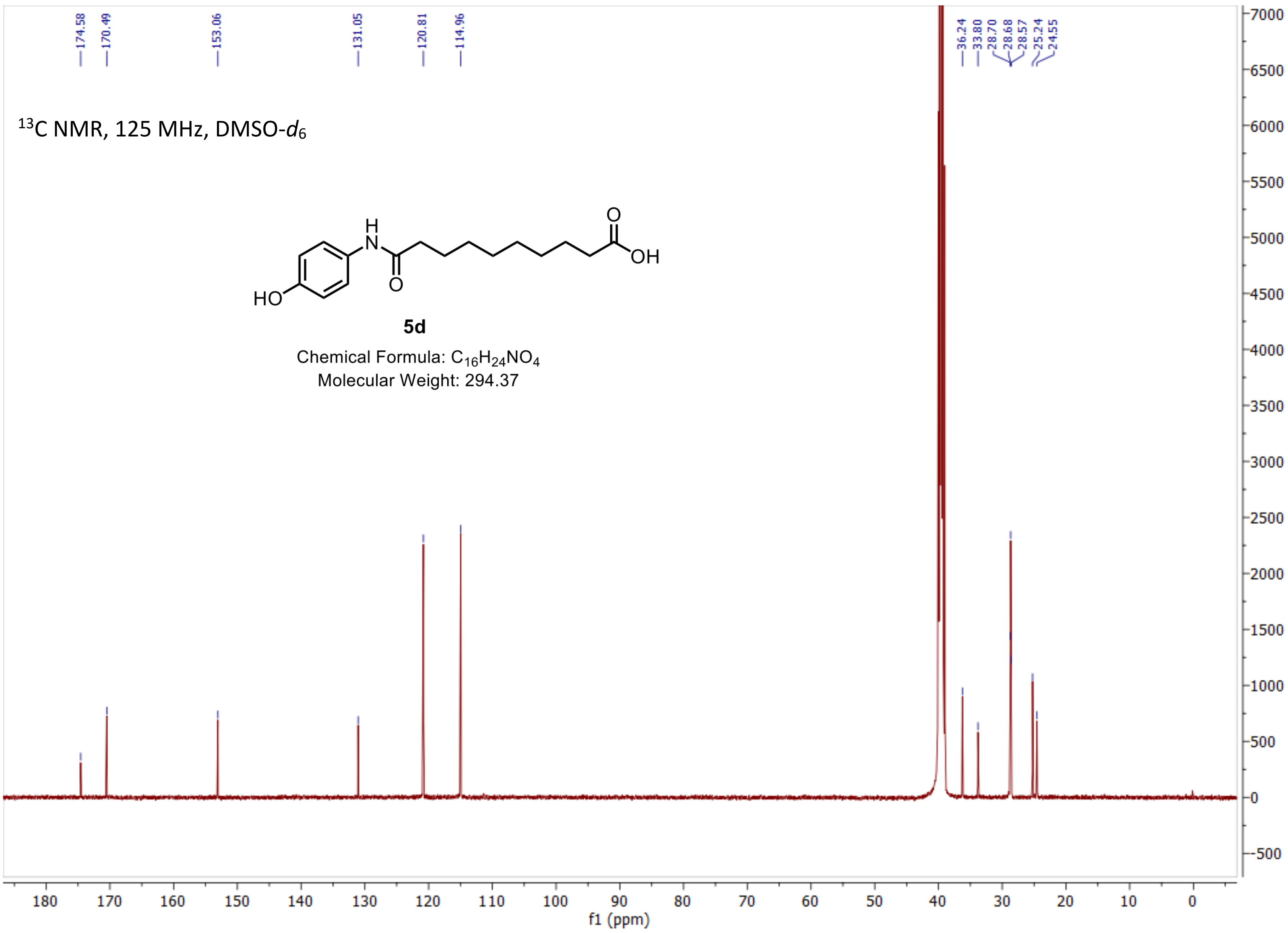

174.58
170.49
153.06
131.05
120.81
114.96
36.24
33.80
28.70
28.68
28.57
25.24
24.55
^{13}C NMR, 125 MHz, DMSO-d_6
HO
H
N
O
O
OH
5d
Chemical Formula: $C_{16}H_{24}NO_4$
Molecular Weight: 294.37
f1 (ppm)

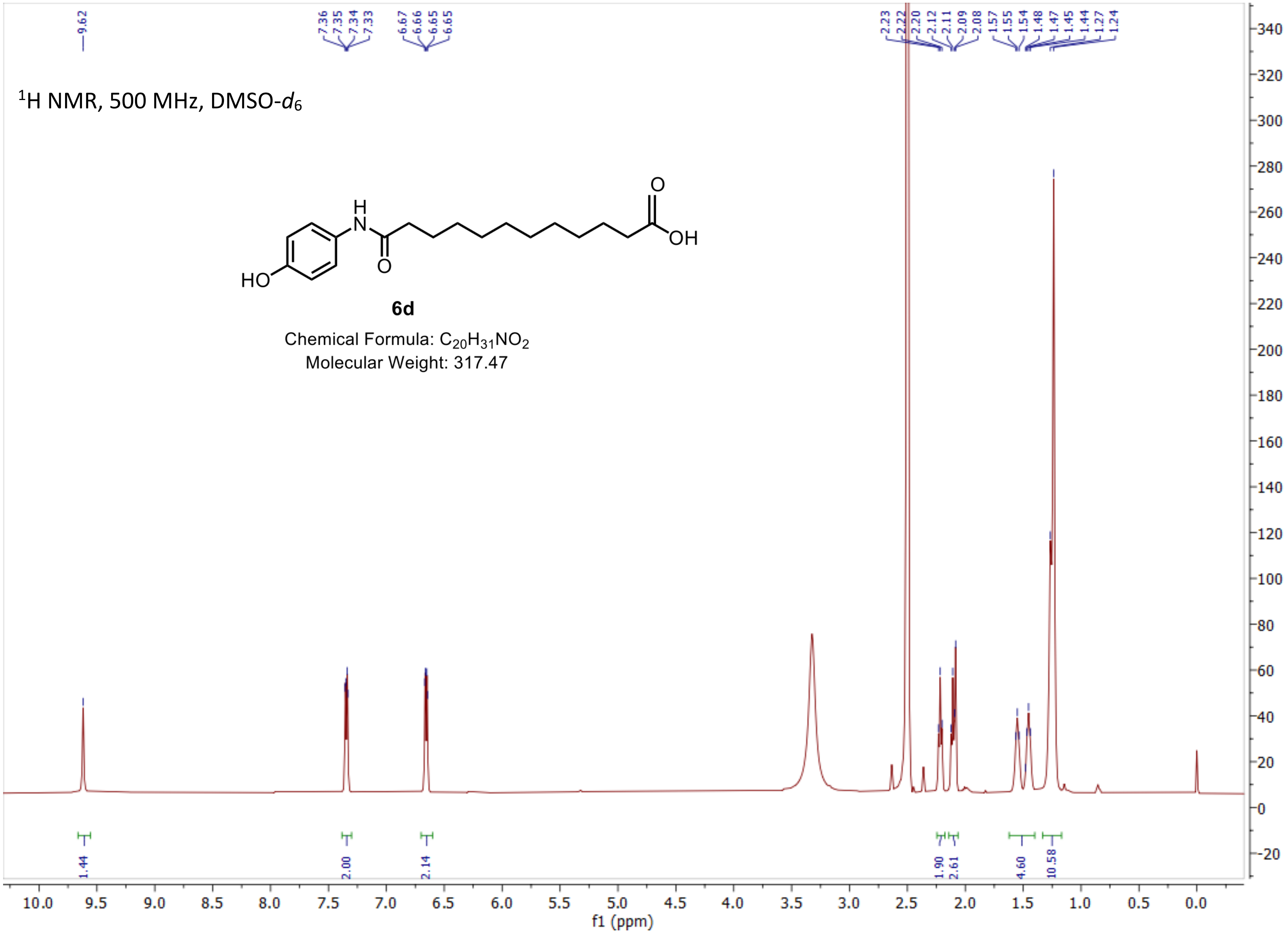

^{1}H NMR, 500 MHz, DMSO-d_6
6d
Chemical Formula: $C_{20}H_{31}NO_2$
Molecular Weight: 317.47
9.62
7.36 7.35 7.34 7.33
6.67 6.66 6.65 6.65
2.23 2.22 2.20 2.12 2.11 2.09 2.08 1.57 1.55 1.54 1.48 1.47 1.45 1.44 1.27 1.24
1.44 2.00 2.14 1.90 2.61 4.60 10.58
f1 (ppm)

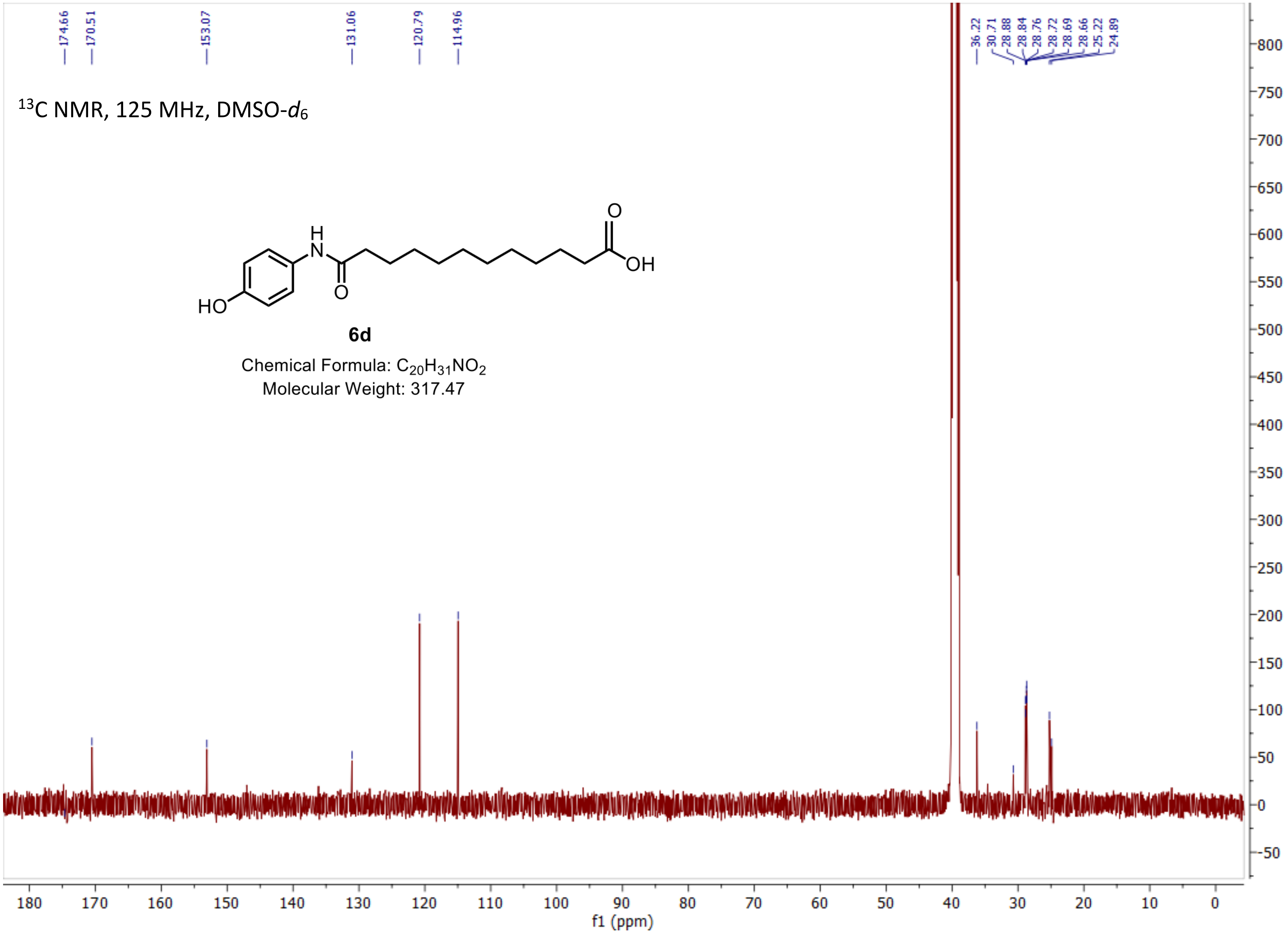

174.66
170.51
153.07
131.06
120.79
114.96
36.22
30.71
28.88
28.84
28.76
28.72
28.69
28.66
25.22
24.89
13C NMR, 125 MHz, DMSO-d6
H
N
O
O
OH
HO
6d
Chemical Formula: C20H31NO2
Molecular Weight: 317.47
f1 (ppm)

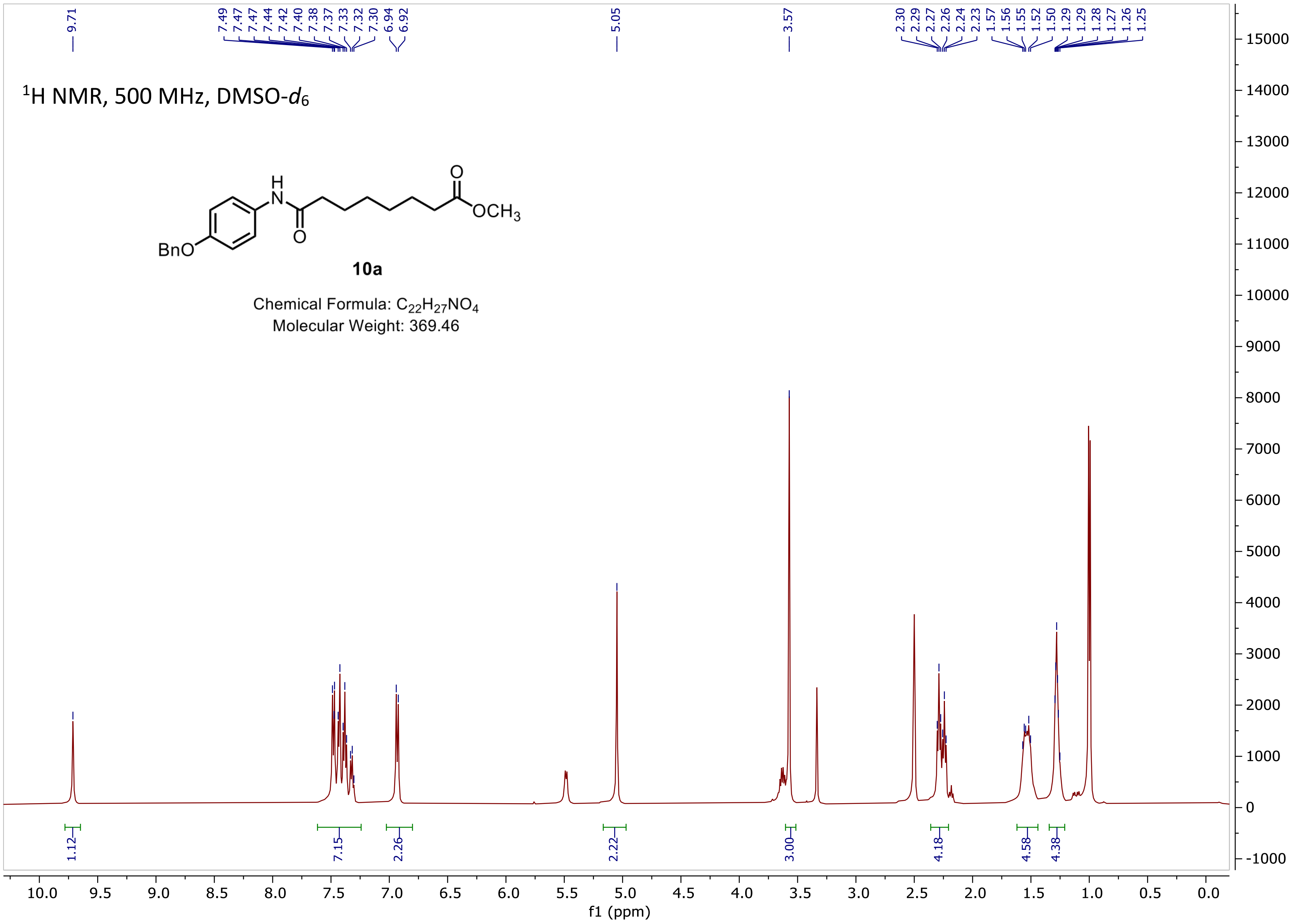

1H NMR, 500 MHz, DMSO-d6
BnO
H
N
O
O
OCH3
10a
Chemical Formula: C22H27NO4
Molecular Weight: 369.46
9.71
7.49 7.47 7.47 7.44 7.42 7.40 7.38 7.37 7.33 7.32 7.30 6.94 6.92
5.05
3.57
2.30 2.29 2.27 2.26 2.24 2.23 1.57 1.56 1.55 1.52 1.50 1.29 1.29 1.28 1.27 1.26 1.25
1.12 7.15 2.26 2.22 3.00 4.18 4.58 4.38
f1 (ppm)

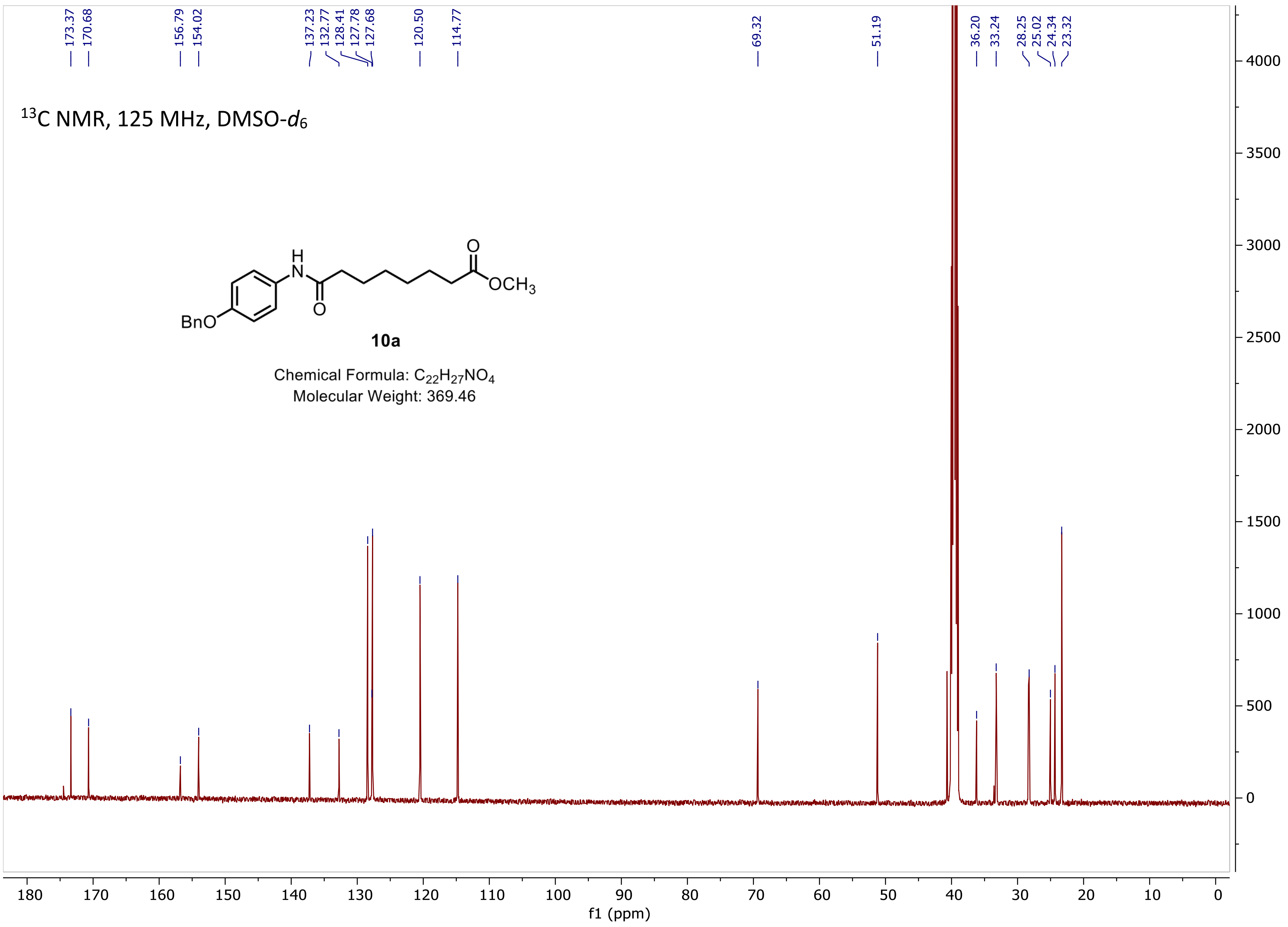
173.37
170.68
156.79
154.02
137.23
132.77
128.41
127.78
127.68
120.50
114.77
69.32
51.19
36.20
33.24
28.25
25.02
24.34
23.32
13C NMR, 125 MHz, DMSO-d6
H
N
O
OCH3
BnO
O
10a
Chemical Formula: C22H27NO4
Molecular Weight: 369.46
4000
3500
3000
2500
2000
1500
1000
500
0
180
170
160
150
140
130
120
110
100
90
80
70
60
50
40
30
20
10
0
f1 (ppm)

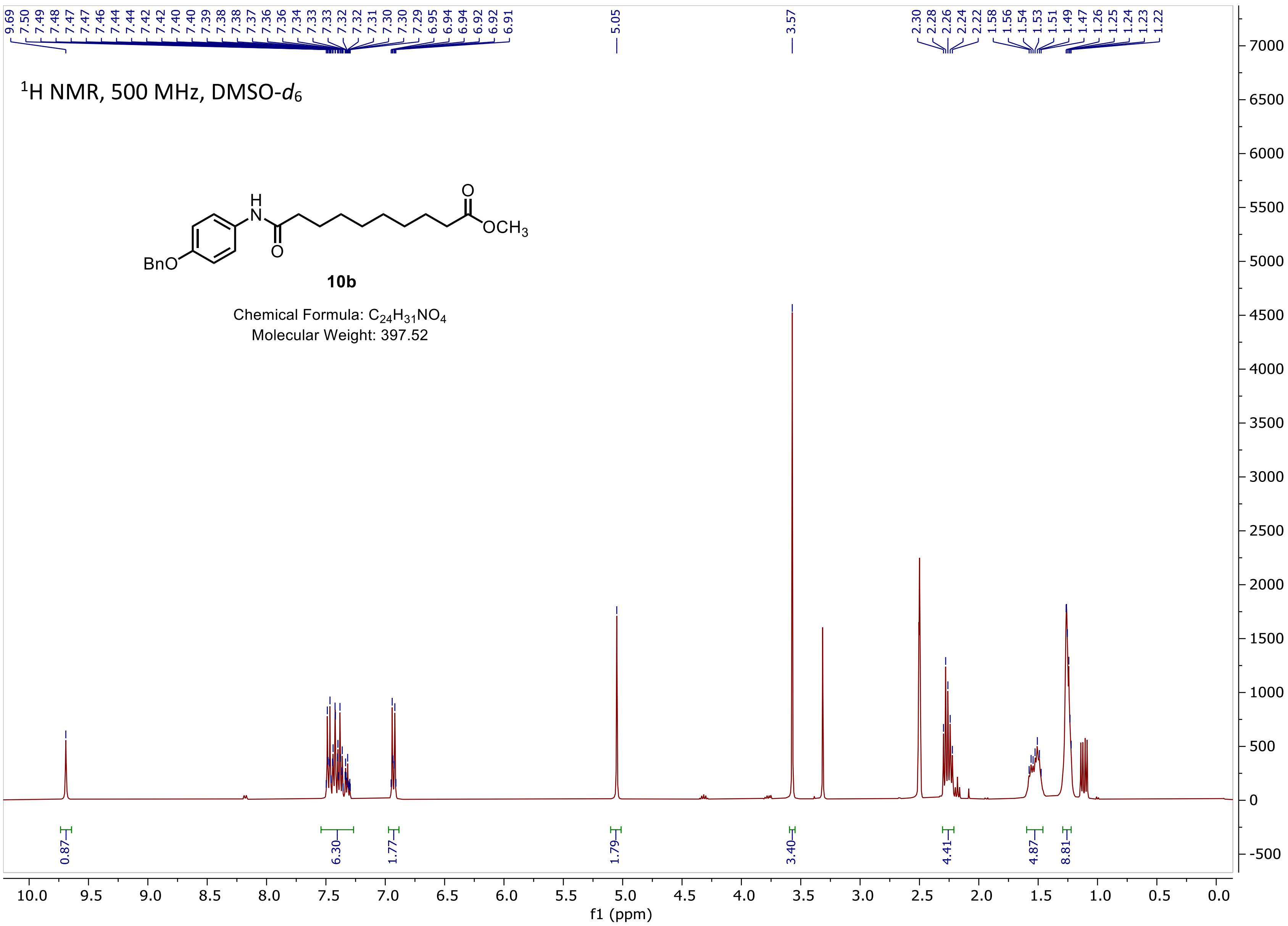

^{1}H NMR, 500 MHz, DMSO-d_6
10b
Chemical Formula: $C_{24}H_{31}NO_4$
Molecular Weight: 397.52
BnO
OCH3
f1 (ppm)

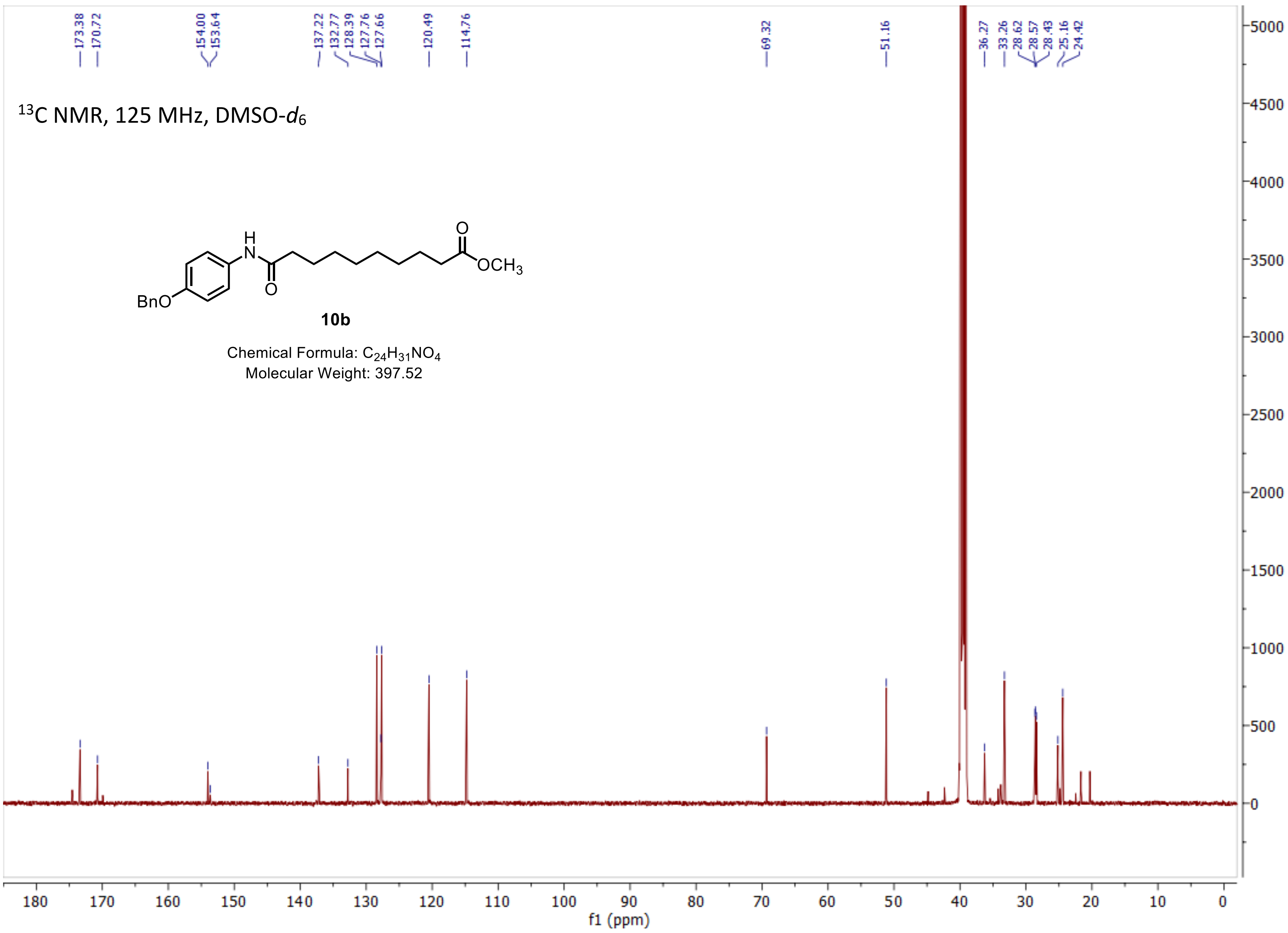

173.38
170.72
154.00
153.64
137.22
132.77
128.39
127.76
127.66
120.49
114.76
69.32
51.16
36.27
33.26
28.62
28.57
28.43
25.16
24.42
13C NMR, 125 MHz, DMSO-d6
BnO
H
N
O
O
OCH3
10b
Chemical Formula: C24H31NO4
Molecular Weight: 397.52
f1 (ppm)

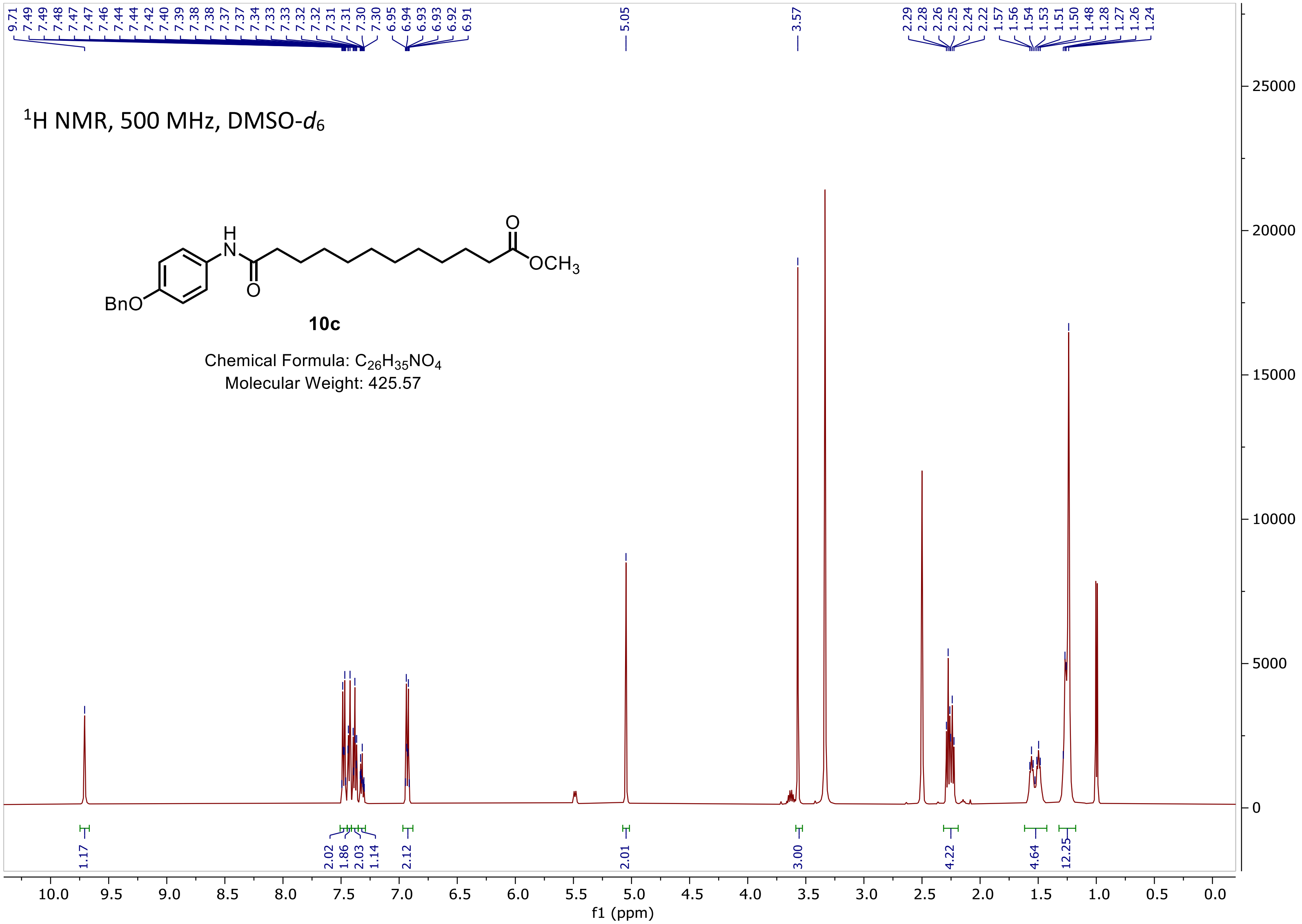

9.71
7.49
7.49
7.48
7.47
7.47
7.46
7.44
7.44
7.42
7.40
7.39
7.38
7.38
7.37
7.37
7.34
7.33
7.33
7.32
7.32
7.31
7.31
7.30
7.30
6.95
6.94
6.93
6.93
6.92
6.91
5.05
3.57
2.29
2.28
2.26
2.25
2.24
2.22
1.57
1.56
1.54
1.53
1.51
1.50
1.48
1.28
1.27
1.26
1.24
1H NMR, 500 MHz, DMSO-d6
H
N
O
OCH3
BnO
O
10c
Chemical Formula: C26H35NO4
Molecular Weight: 425.57
1.17
2.02
1.86
2.03
1.14
2.12
2.01
3.00
4.22
4.64
12.25
f1 (ppm)

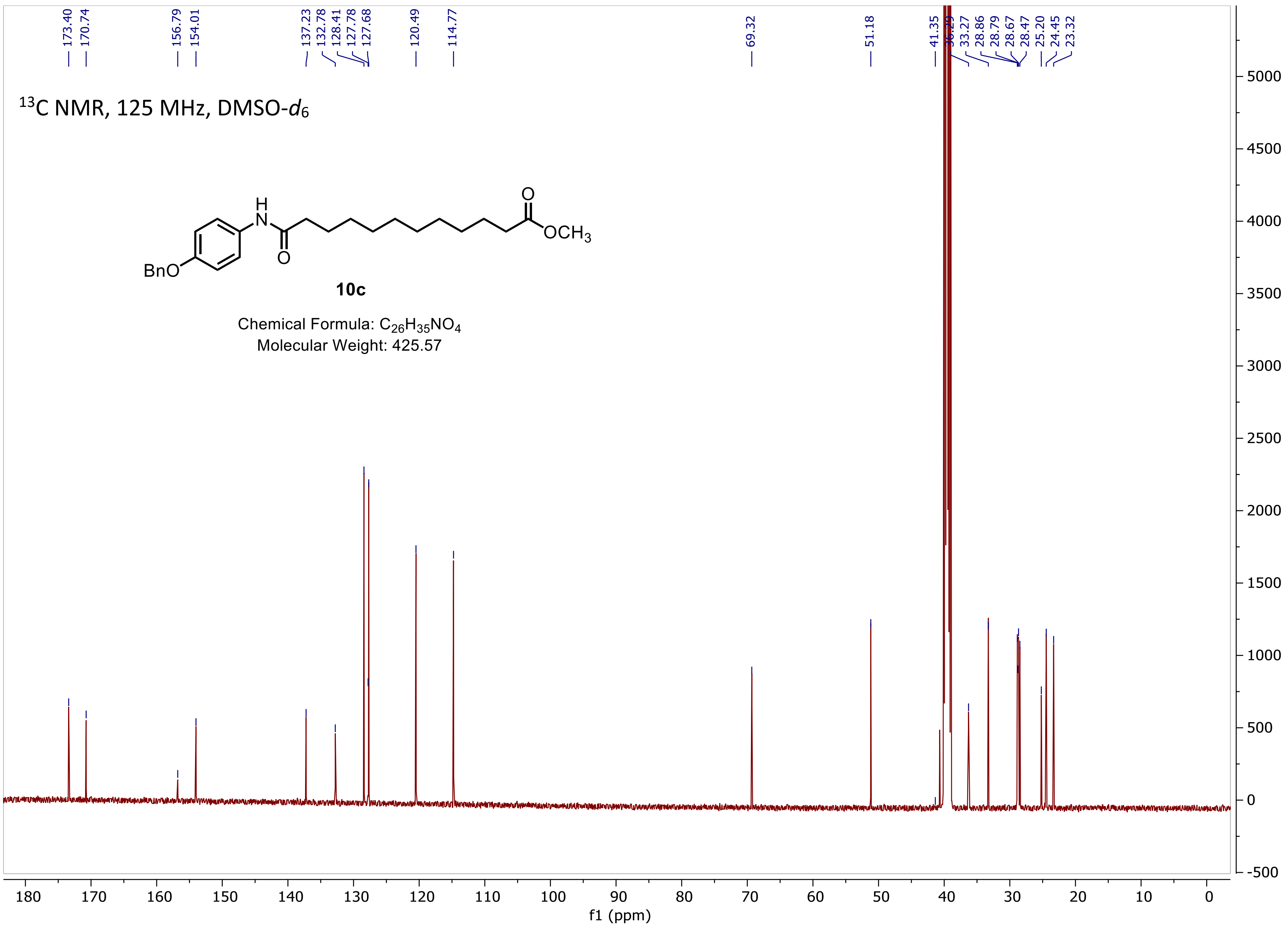
173.40
170.74
156.79
154.01
137.23
132.78
128.41
127.78
127.68
120.49
114.77
69.32
51.18
41.35
36.29
33.27
28.86
28.79
28.67
28.47
25.20
24.45
23.32
13C NMR, 125 MHz, DMSO-d6
H
N
O
O
OCH3
BnO
10c
Chemical Formula: C26H35NO4
Molecular Weight: 425.57
f1 (ppm)

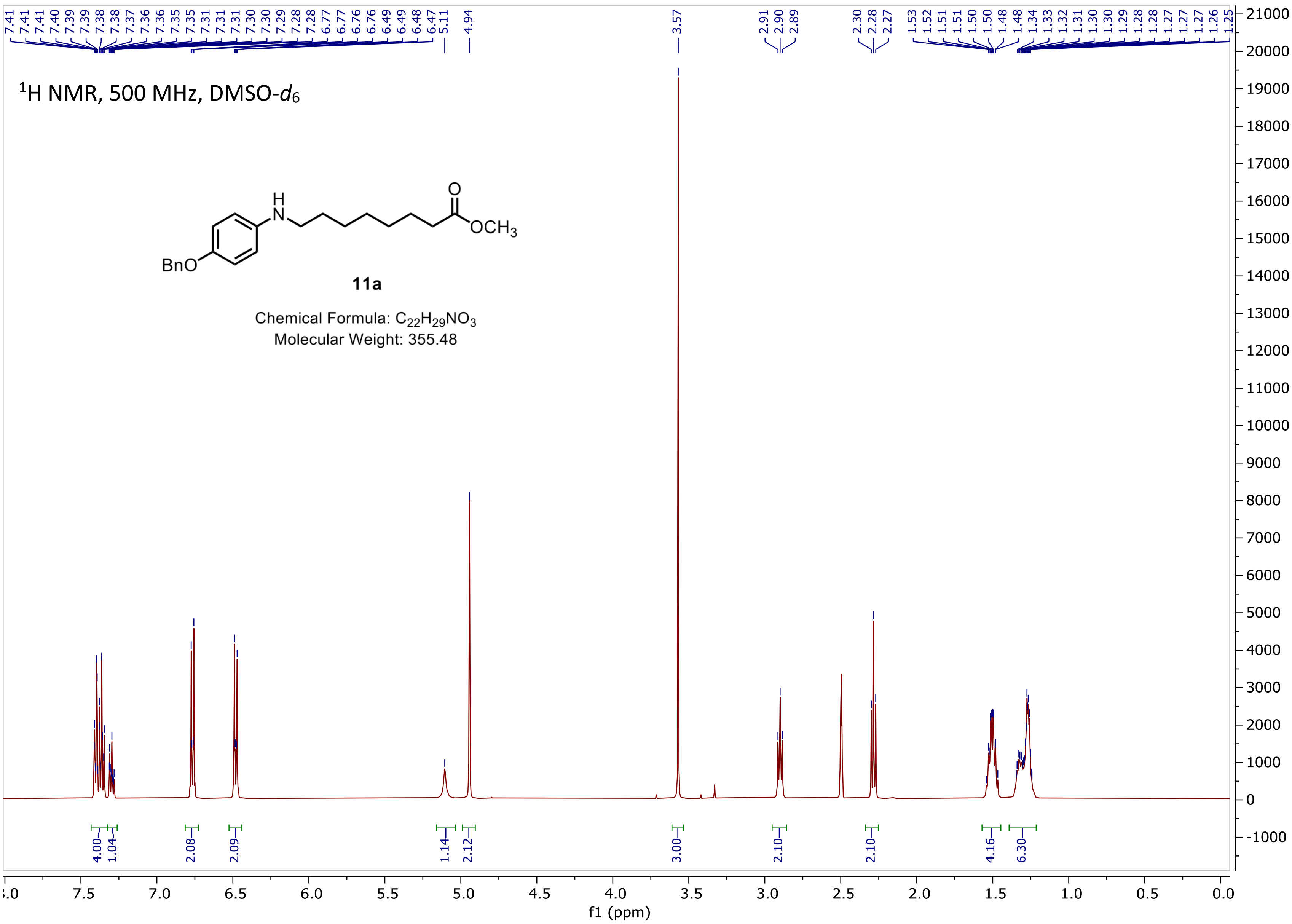

¹H NMR, 500 MHz, DMSO-d6
11a
Chemical Formula: C22H29NO3
Molecular Weight: 355.48
f1 (ppm)

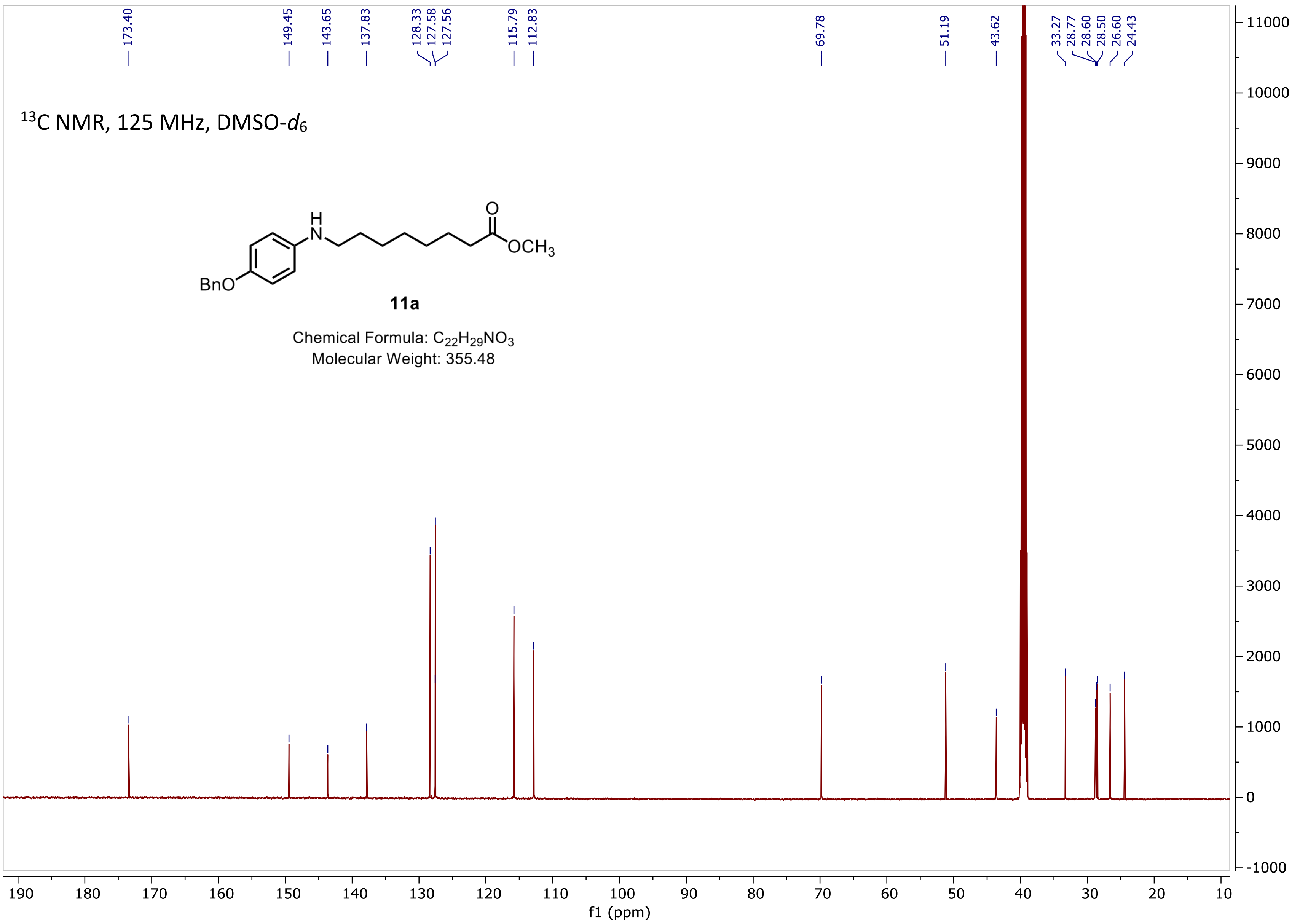

173.40
149.45
143.65
137.83
128.33
127.58
127.56
115.79
112.83
69.78
51.19
43.62
33.27
28.77
28.60
28.50
26.60
24.43
13C NMR, 125 MHz, DMSO-d6
BnO
H
N
O
OCH3
11a
Chemical Formula: C22H29NO3
Molecular Weight: 355.48
f1 (ppm)

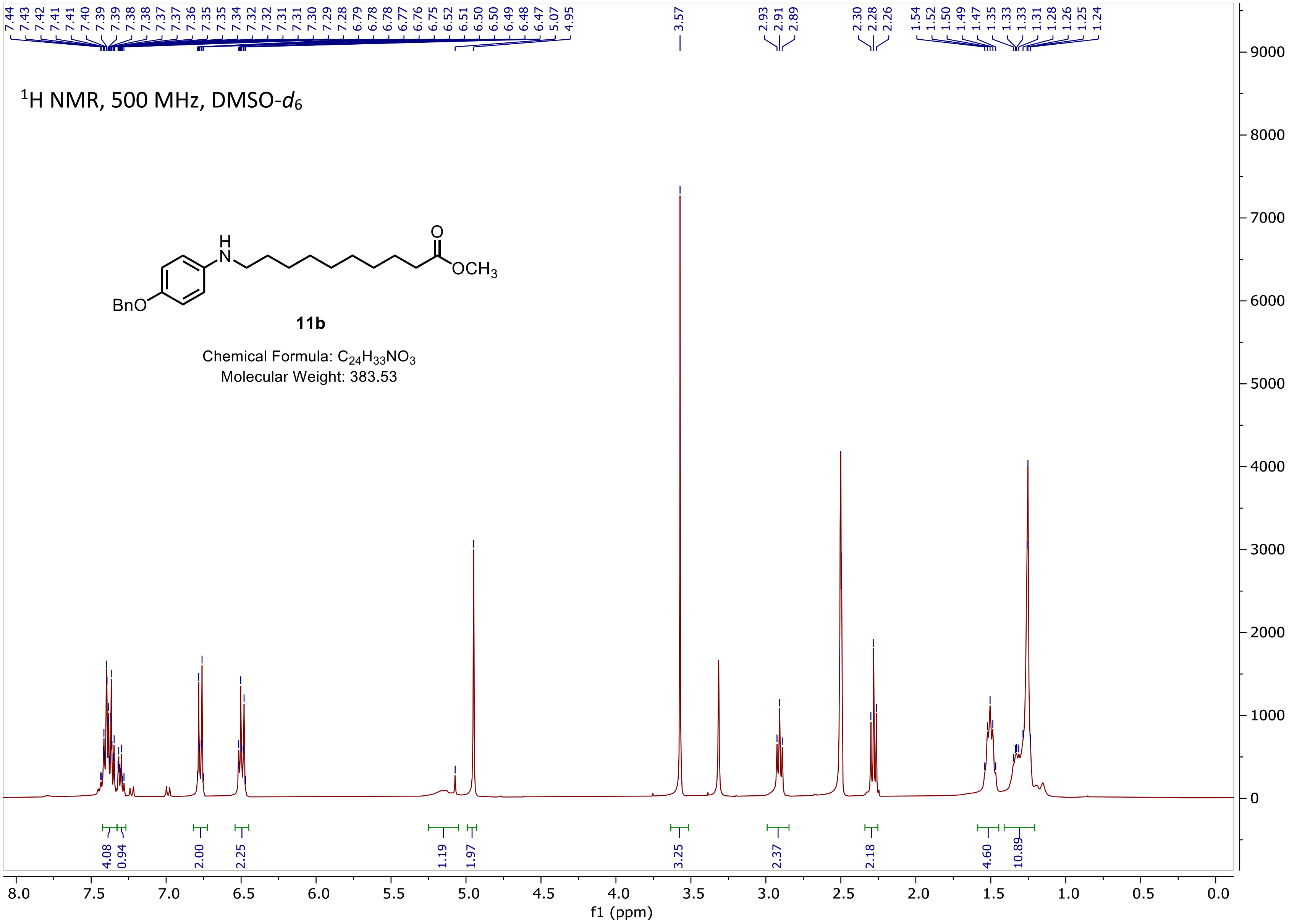

7.44 7.43 7.42 7.41 7.41 7.40 7.39 7.39 7.38 7.38 7.37 7.37 7.36 7.35 7.35 7.34 7.32 7.32 7.31 7.31 7.30 7.29 7.28 6.79 6.78 6.78 6.77 6.76 6.75 6.52 6.51 6.50 6.50 6.49 6.48 6.47 5.07 4.95
3.57
2.93 2.91 2.89
2.30 2.28 2.26
1.54 1.52 1.50 1.49 1.47 1.35 1.33 1.33 1.31 1.28 1.26 1.25 1.24
1H NMR, 500 MHz, DMSO-d6
H
N
O
OCH3
BnO
11b
Chemical Formula: C24H33NO3
Molecular Weight: 383.53
4.08 0.94 2.00 2.25 1.19 1.97 3.25 2.37 2.18 4.60 10.89
8.0 7.5 7.0 6.5 6.0 5.5 5.0 4.5 4.0 3.5 3.0 2.5 2.0 1.5 1.0 0.5 0.0
f1 (ppm)
9000 8000 7000 6000 5000 4000 3000 2000 1000 0

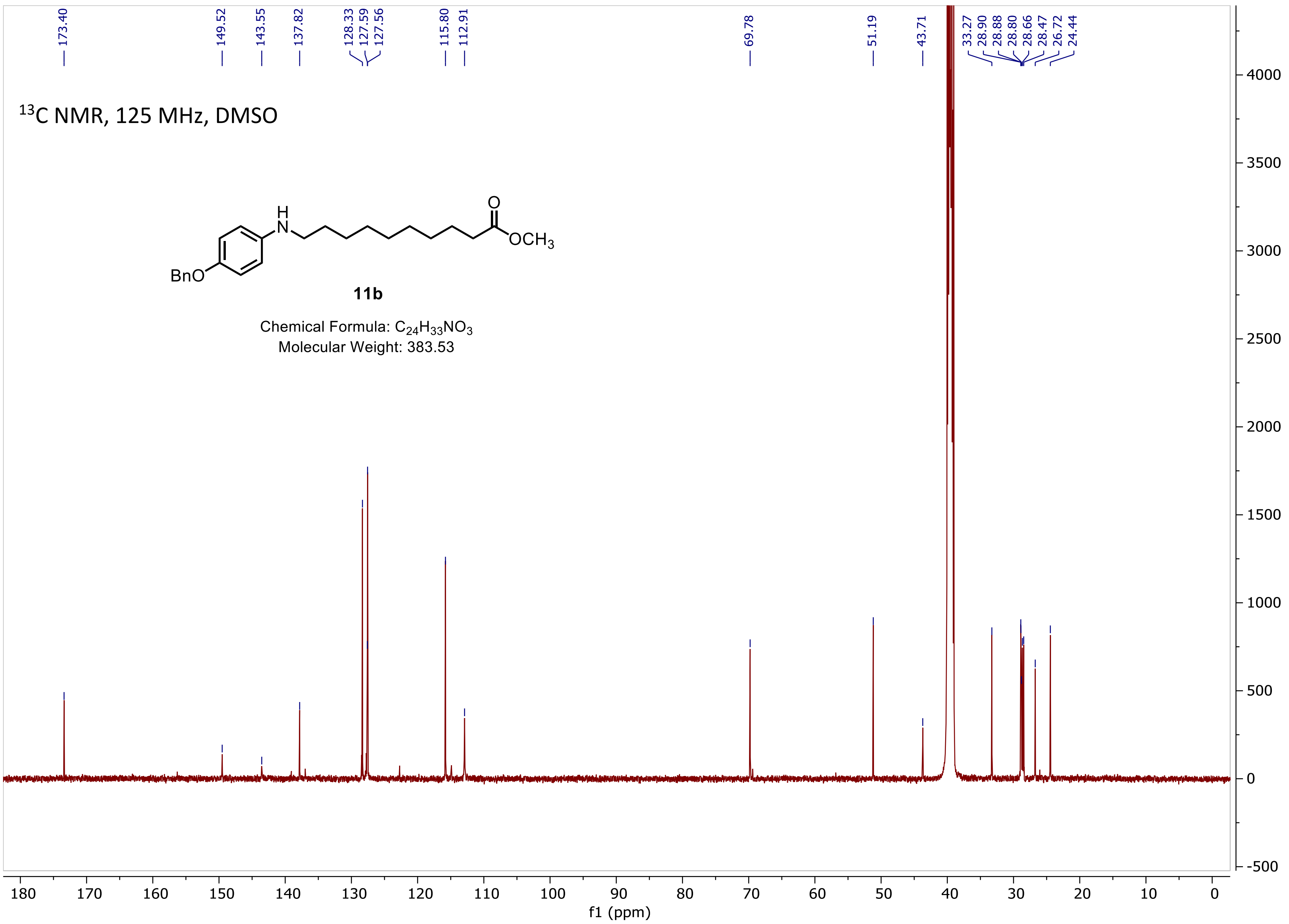

173.40
149.52
143.55
137.82
128.33
127.59
127.56
115.80
112.91
69.78
51.19
43.71
33.27
28.90
28.88
28.80
28.66
28.47
26.72
24.44
13C NMR, 125 MHz, DMSO
BnO
H
N
O
OCH3
11b
Chemical Formula: C24H33NO3
Molecular Weight: 383.53
f1 (ppm)

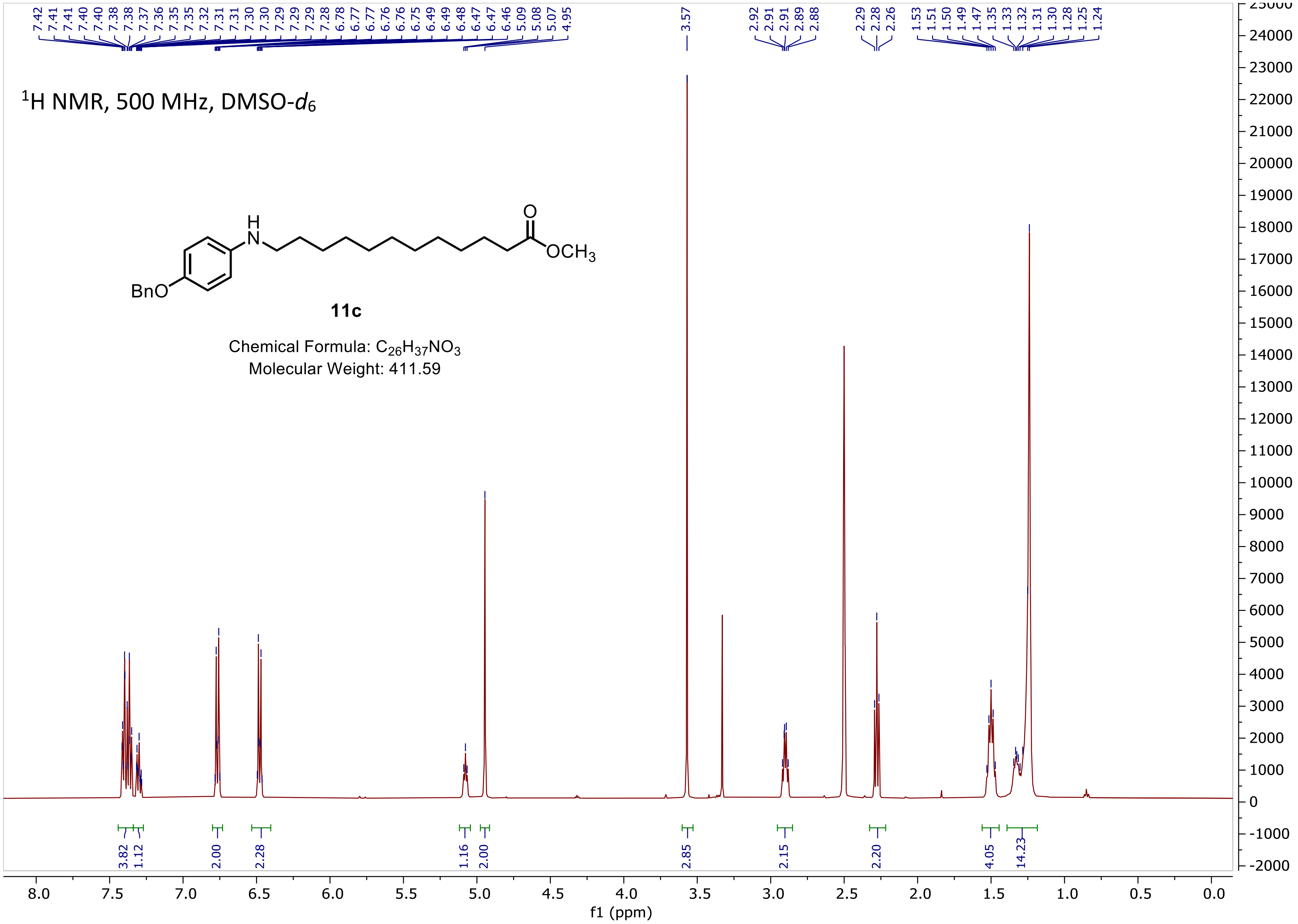

7.42
7.41
7.41
7.40
7.40
7.38
7.38
7.37
7.36
7.35
7.35
7.32
7.31
7.31
7.30
7.30
7.29
7.29
7.29
7.28
6.78
6.77
6.77
6.76
6.76
6.75
6.49
6.49
6.48
6.47
6.47
6.46
5.09
5.08
5.07
4.95
3.57
2.92
2.91
2.91
2.89
2.88
2.29
2.28
2.26
1.53
1.51
1.50
1.49
1.47
1.35
1.33
1.32
1.31
1.30
1.28
1.25
1.24
¹H NMR, 500 MHz, DMSO-d6
H
N
O
OCH3
BnO
11c
Chemical Formula: C26H37NO3
Molecular Weight: 411.59
3.82
1.12
2.00
2.28
1.16
2.00
2.85
2.15
2.20
4.05
14.23
f1 (ppm)

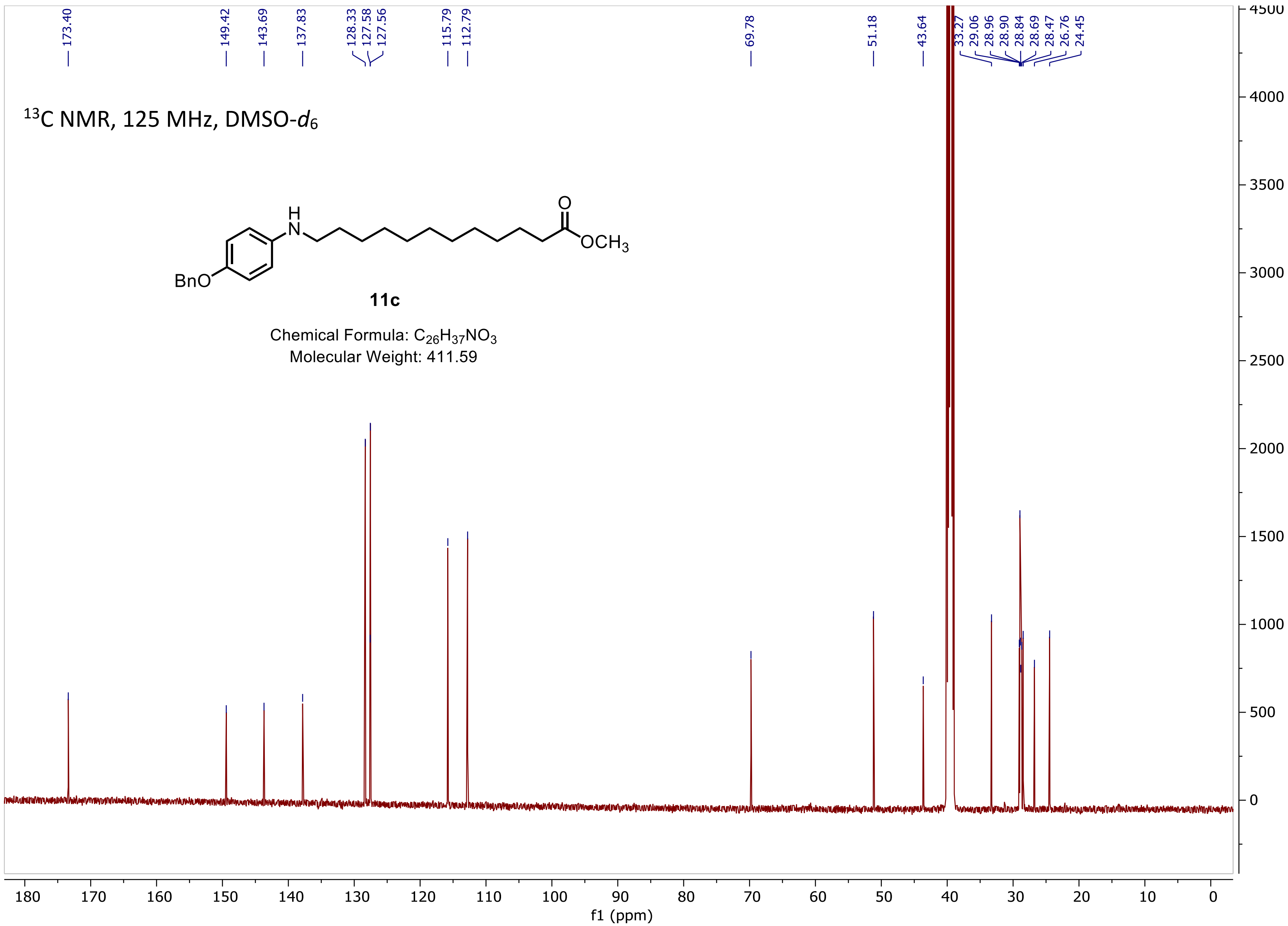
173.40
149.42
143.69
137.83
128.33
127.58
127.56
115.79
112.79
69.78
51.18
43.64
33.27
29.06
28.96
28.90
28.84
28.69
28.47
26.76
24.45
13C NMR, 125 MHz, DMSO-d6
H
N
O
OCH3
BnO
11c
Chemical Formula: C26H37NO3
Molecular Weight: 411.59
f1 (ppm)

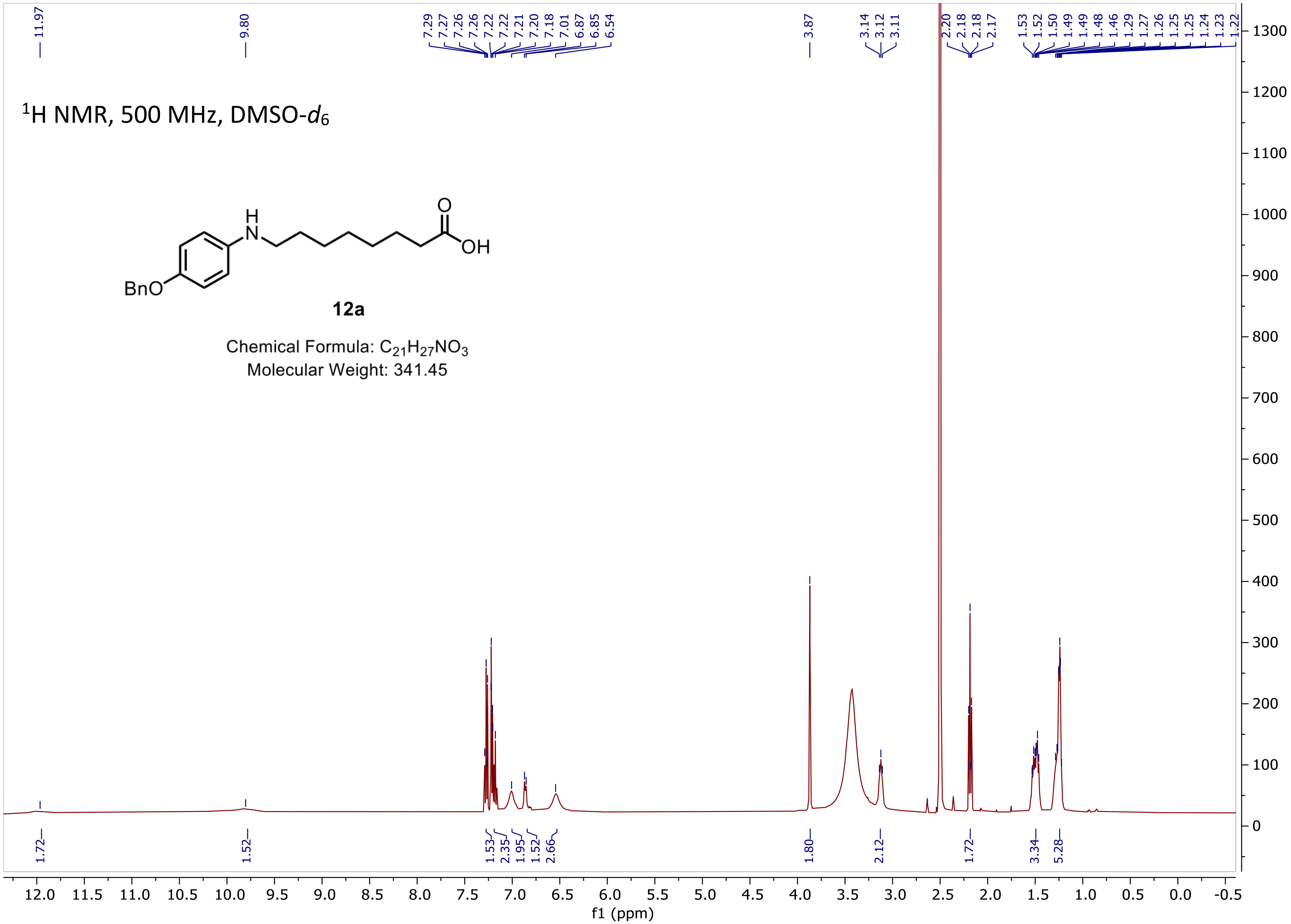

^{1}H NMR, 500 MHz, DMSO-d_6
BnO
H
N
O
OH
12a
Chemical Formula: $C_{21}H_{27}NO_3$
Molecular Weight: 341.45
11.97
9.80
7.29 7.27 7.26 7.26 7.22 7.22 7.21 7.20 7.18 7.01 6.87 6.85 6.54
3.87
3.14 3.12 3.11
2.20 2.18 2.18 2.17
1.53 1.52 1.50 1.49 1.48 1.46 1.29 1.27 1.26 1.25 1.25 1.24 1.23 1.22
1.72 1.52 1.53 2.35 1.95 1.52 2.66 1.80 2.12 1.72 3.34 5.28
f1 (ppm)

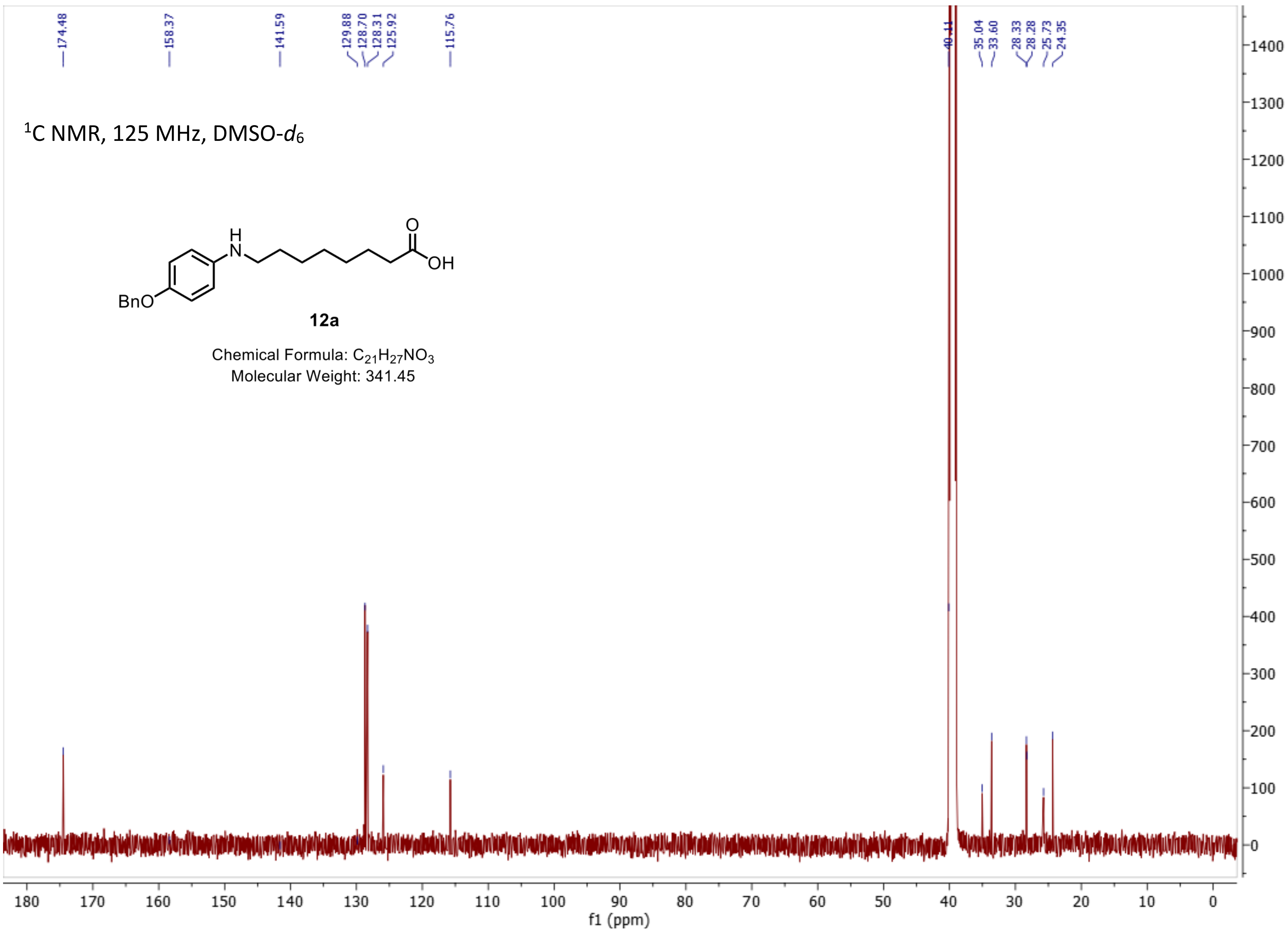
174.48
158.37
141.59
129.88
128.70
128.31
125.92
115.76
40.11
35.04
33.60
28.33
28.28
25.73
24.35
1C NMR, 125 MHz, DMSO-d6
H
N
O
OH
BnO
12a
Chemical Formula: C21H27NO3
Molecular Weight: 341.45
f1 (ppm)

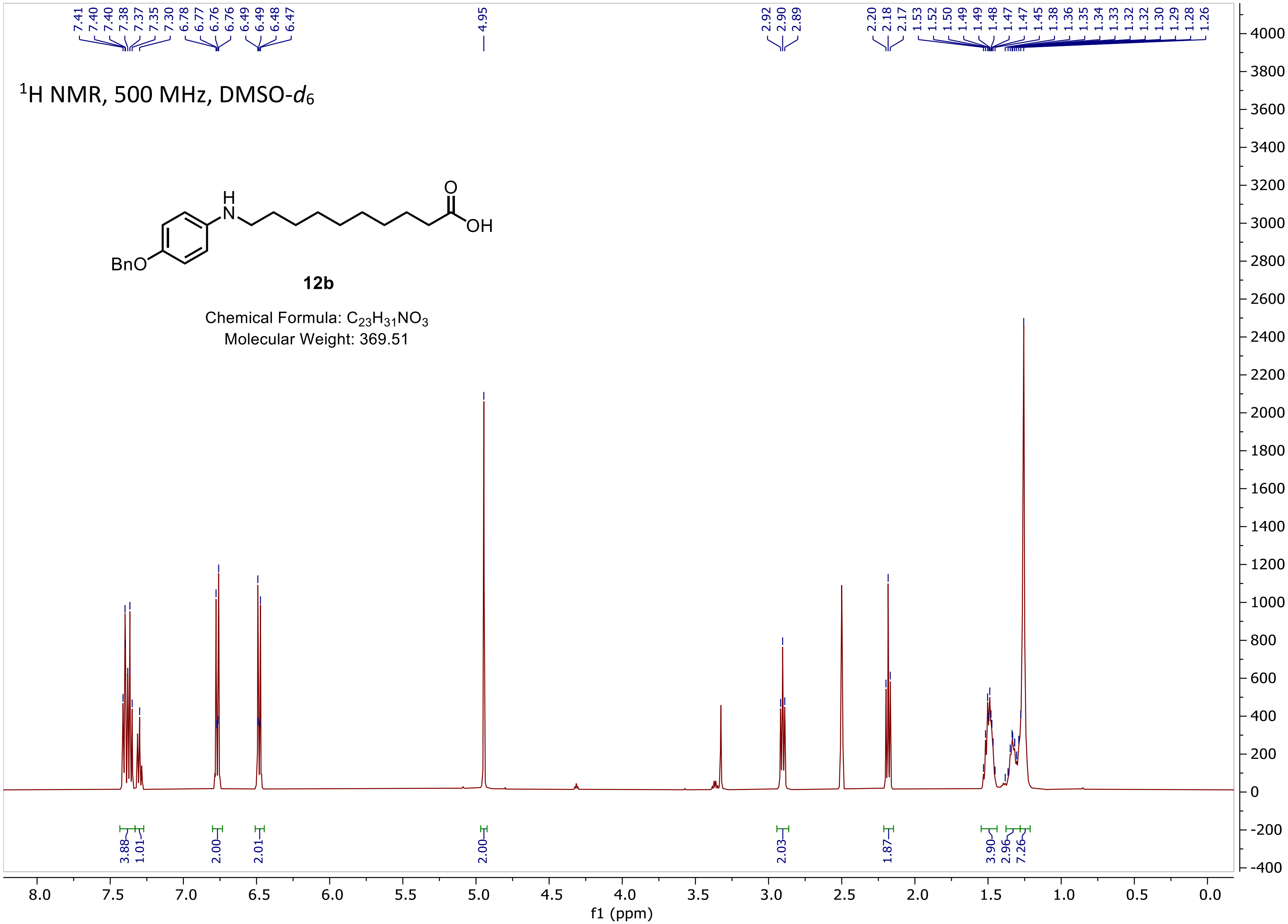

¹H NMR, 500 MHz, DMSO-d6
BnO
H
N
O
OH
12b
Chemical Formula: C23H31NO3
Molecular Weight: 369.51
7.41 7.40 7.40 7.38 7.37 7.35 7.30 6.78 6.77 6.76 6.76 6.49 6.49 6.48 6.47
4.95
2.92 2.90 2.89
2.20 2.18 2.17 1.53 1.52 1.50 1.49 1.49 1.48 1.47 1.47 1.45 1.38 1.36 1.35 1.34 1.33 1.32 1.32 1.30 1.29 1.28 1.26
3.88 1.01 2.00 2.01 2.00 2.03 1.87 3.90 2.96 7.26
f1 (ppm)

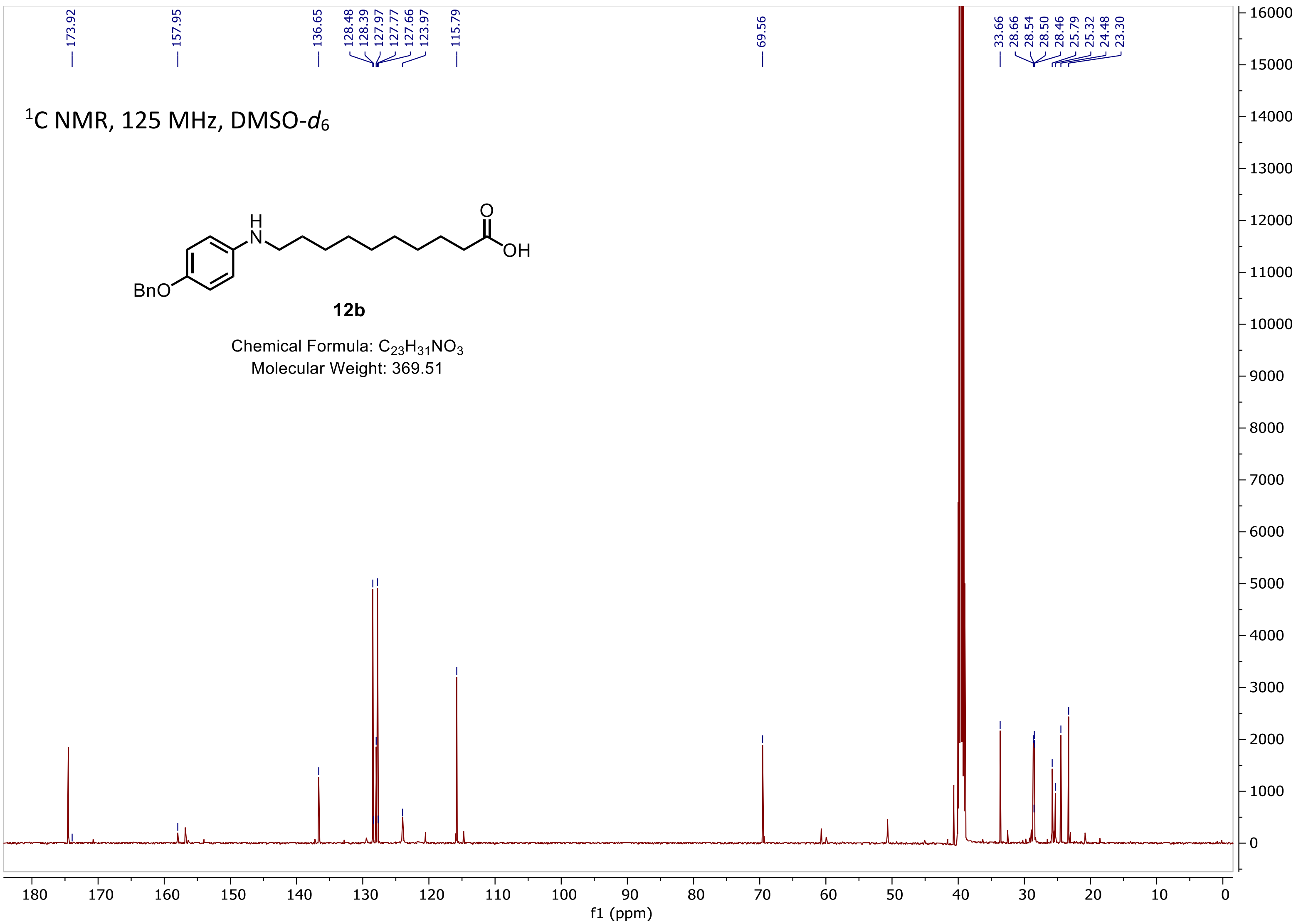

173.92
157.95
136.65
128.48
128.39
127.97
127.77
127.66
123.97
115.79
69.56
33.66
28.66
28.54
28.50
28.46
25.79
25.32
24.48
23.30
1C NMR, 125 MHz, DMSO-d6
H
N
O
OH
BnO
12b
Chemical Formula: C23H31NO3
Molecular Weight: 369.51
f1 (ppm)

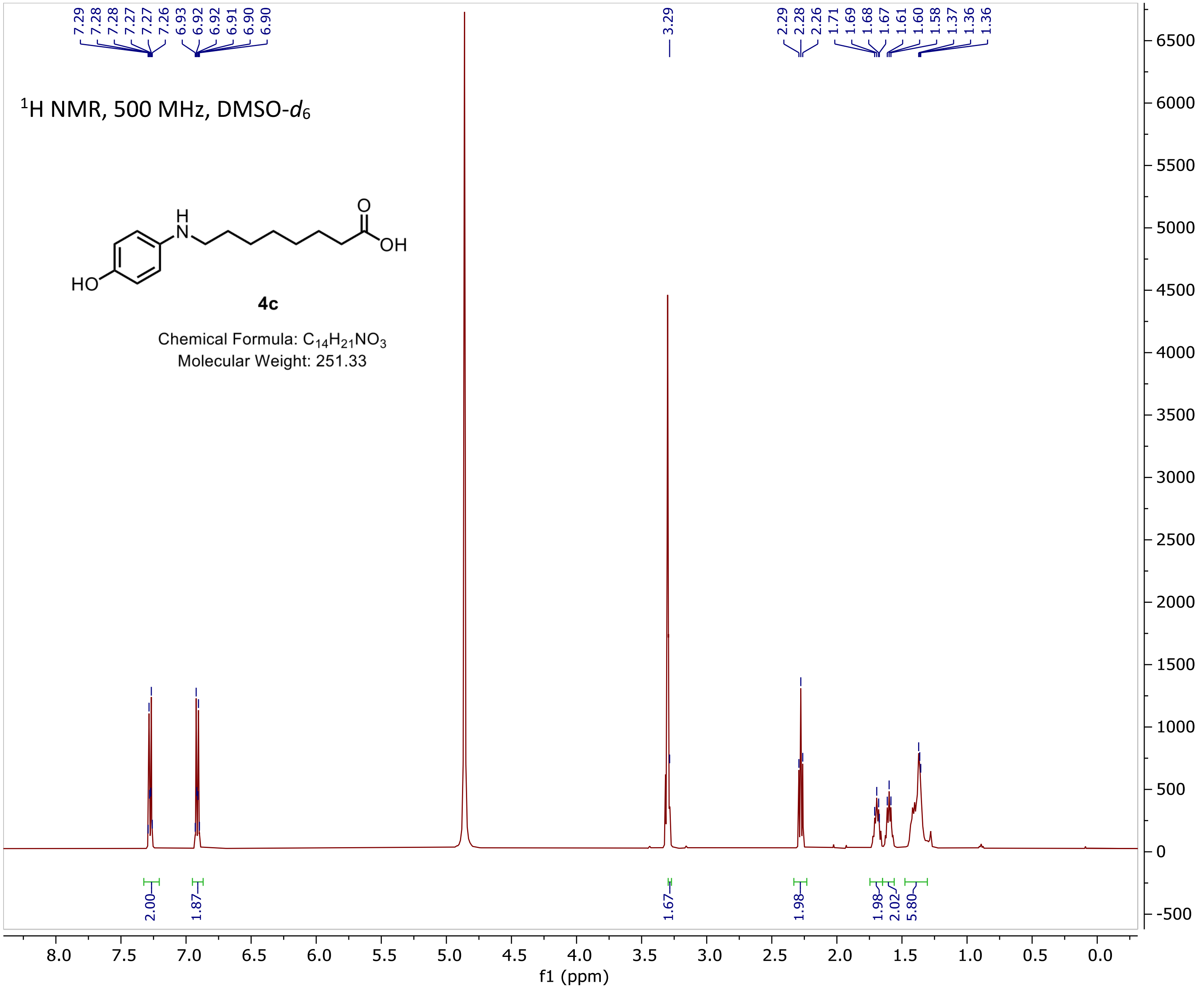
7.29
7.28
7.28
7.27
7.27
7.26
6.93
6.92
6.92
6.91
6.90
6.90
3.29
2.29
2.28
2.26
1.71
1.69
1.68
1.67
1.61
1.60
1.58
1.37
1.36
1.36
^{1}H NMR, 500 MHz, DMSO-d_6
H
N
O
OH
HO
4c
Chemical Formula: $C_{14}H_{21}NO_3$
Molecular Weight: 251.33
2.00
1.87
1.67
1.98
1.98
2.02
5.80
f1 (ppm)

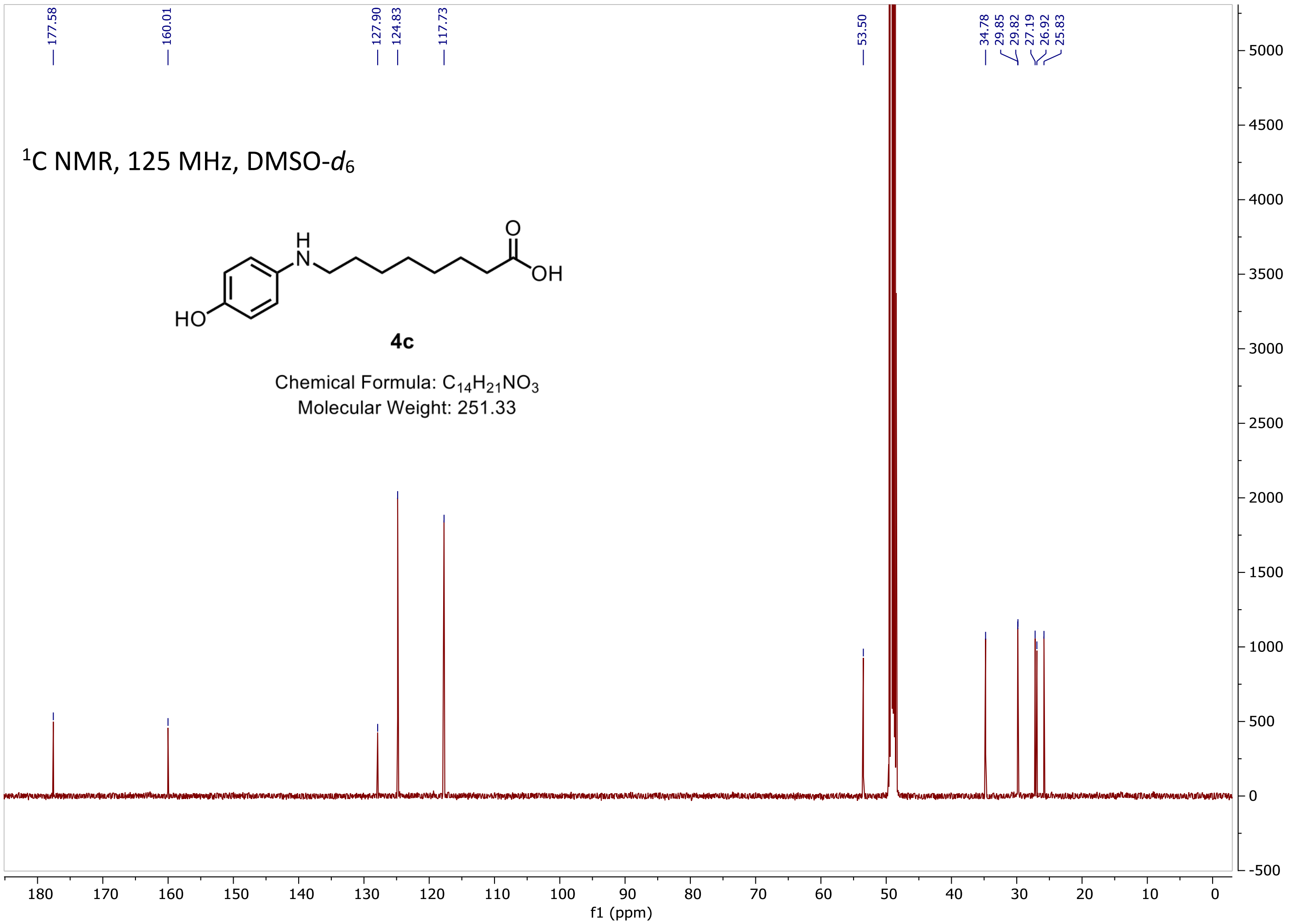
177.58
160.01
127.90
124.83
117.73
53.50
34.78
29.85
29.82
27.19
26.92
25.83
^{1}C NMR, 125 MHz, DMSO-d_6
H
N
O
OH
HO
4c
Chemical Formula: $C_{14}H_{21}NO_3$
Molecular Weight: 251.33
5000
4500
4000
3500
3000
2500
2000
1500
1000
500
0
-500
180 170 160 150 140 130 120 110 100 90 80 70 60 50 40 30 20 10 0
f1 (ppm)

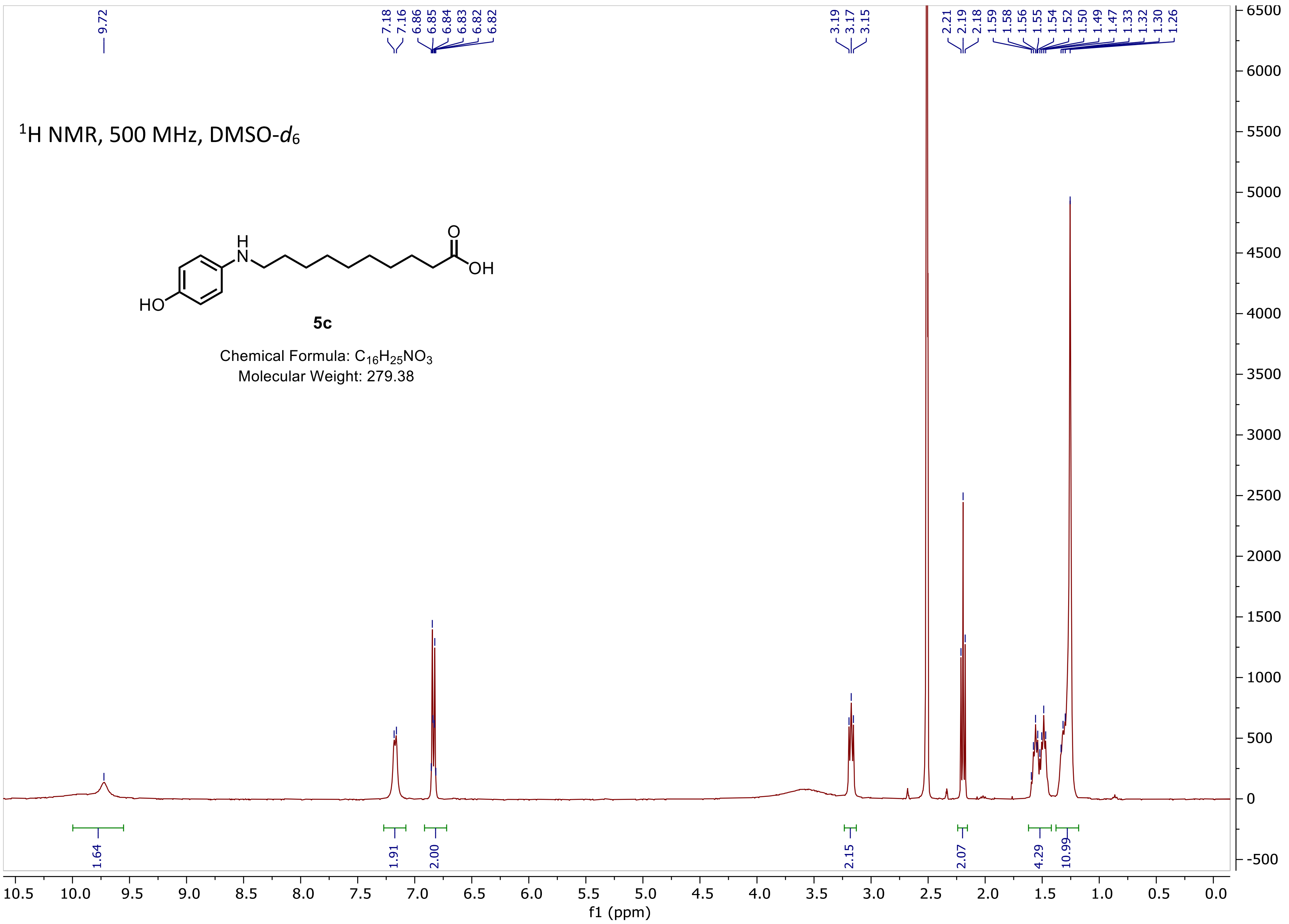

^{1}H NMR, 500 MHz, DMSO-d_6
5c
Chemical Formula: $C_{16}H_{25}NO_3$
Molecular Weight: 279.38
9.72
7.18 7.16 6.86 6.85 6.84 6.83 6.82 6.82
3.19 3.17 3.15
2.21 2.19 2.18 1.59 1.58 1.56 1.55 1.54 1.52 1.50 1.49 1.47 1.33 1.32 1.30 1.26
1.64 1.91 2.00 2.15 2.07 4.29 10.99
f1 (ppm)

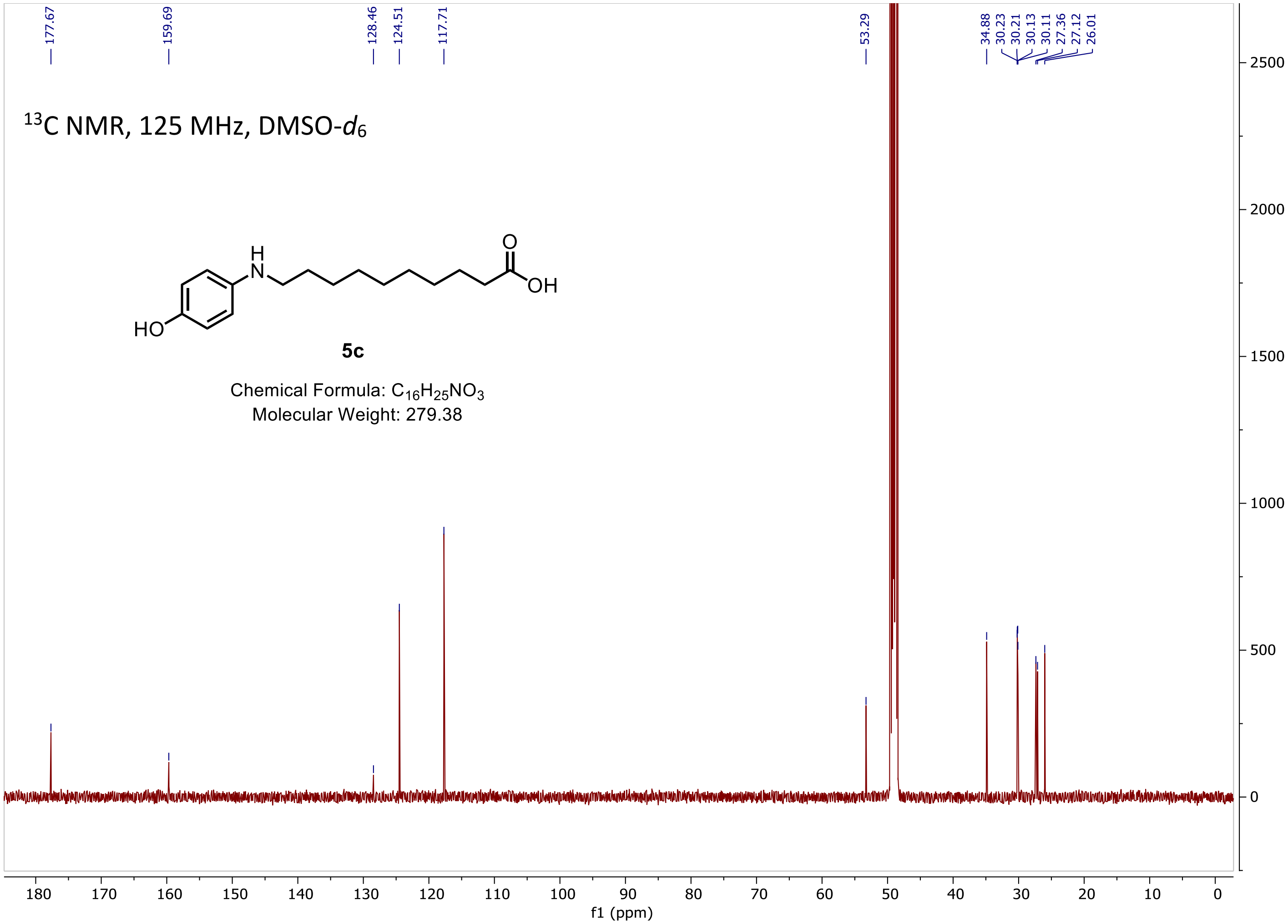

177.67
159.69
128.46
124.51
117.71
53.29
34.88
30.23
30.21
30.13
30.11
27.36
27.12
26.01
13C NMR, 125 MHz, DMSO-d6
H
N
O
OH
HO
5c
Chemical Formula: C16H25NO3
Molecular Weight: 279.38
2500
2000
1500
1000
500
0
180
170
160
150
140
130
120
110
100
90
80
70
60
50
40
30
20
10
0
f1 (ppm)

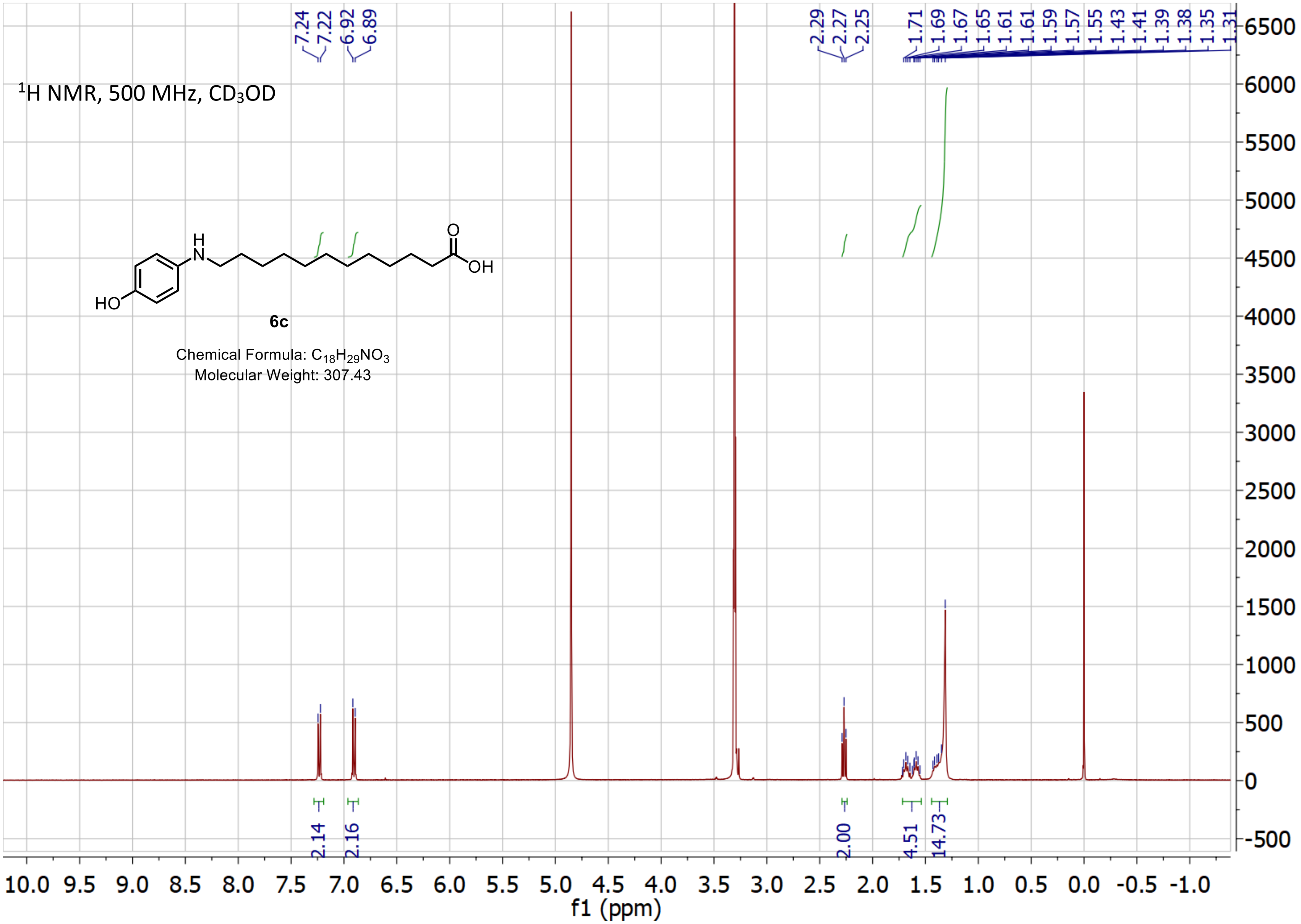

¹H NMR, 500 MHz, CD3OD
6c
Chemical Formula: C18H29NO3
Molecular Weight: 307.43
HO
H
N
O
OH
7.24
7.22
6.92
6.89
2.29
2.27
2.25
1.71
1.69
1.67
1.65
1.61
1.61
1.59
1.57
1.55
1.43
1.41
1.39
1.38
1.35
1.31
2.14
2.16
2.00
4.51
14.73
f1 (ppm)

^{13}C NMR, 125 MHz, CD_3OD

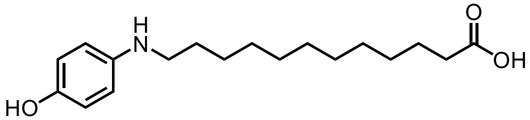

6c

Chemical Formula: $C_{18}H_{29}NO_3$
Molecular Weight: 307.43

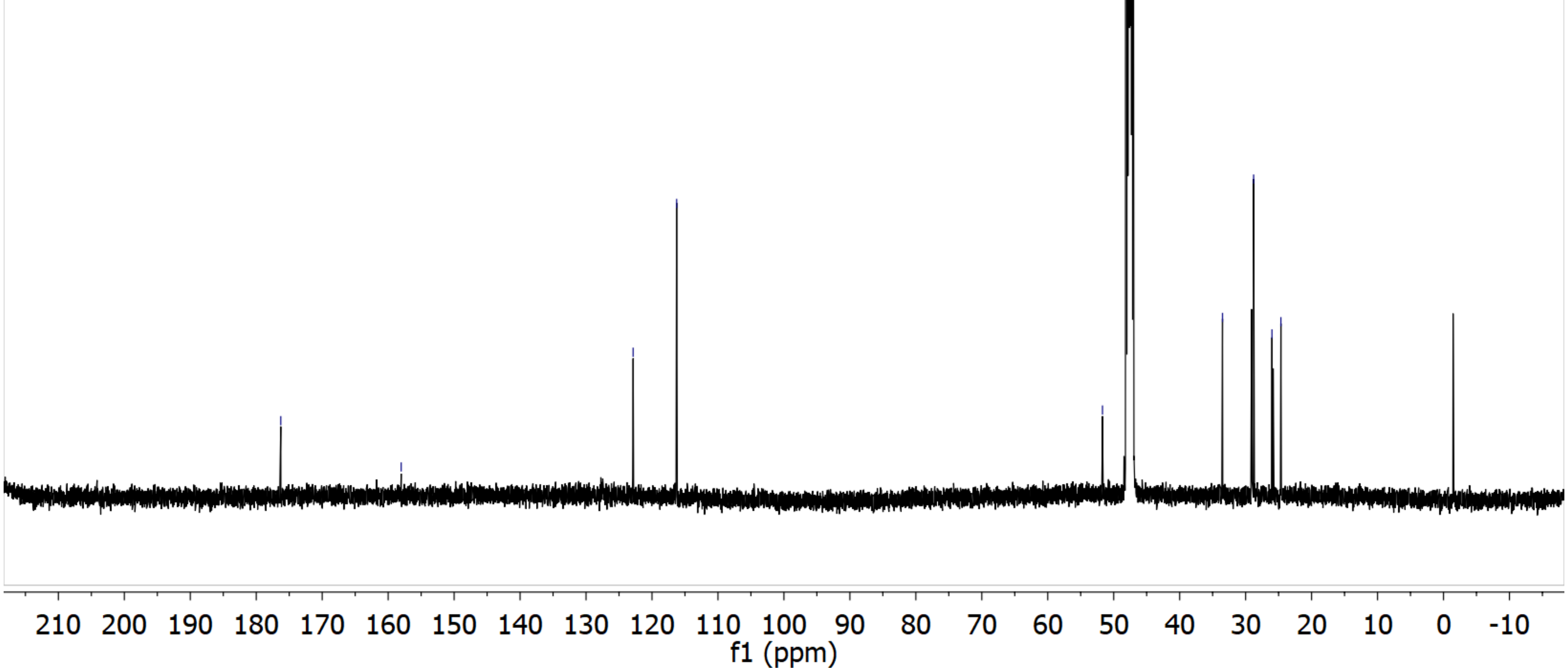

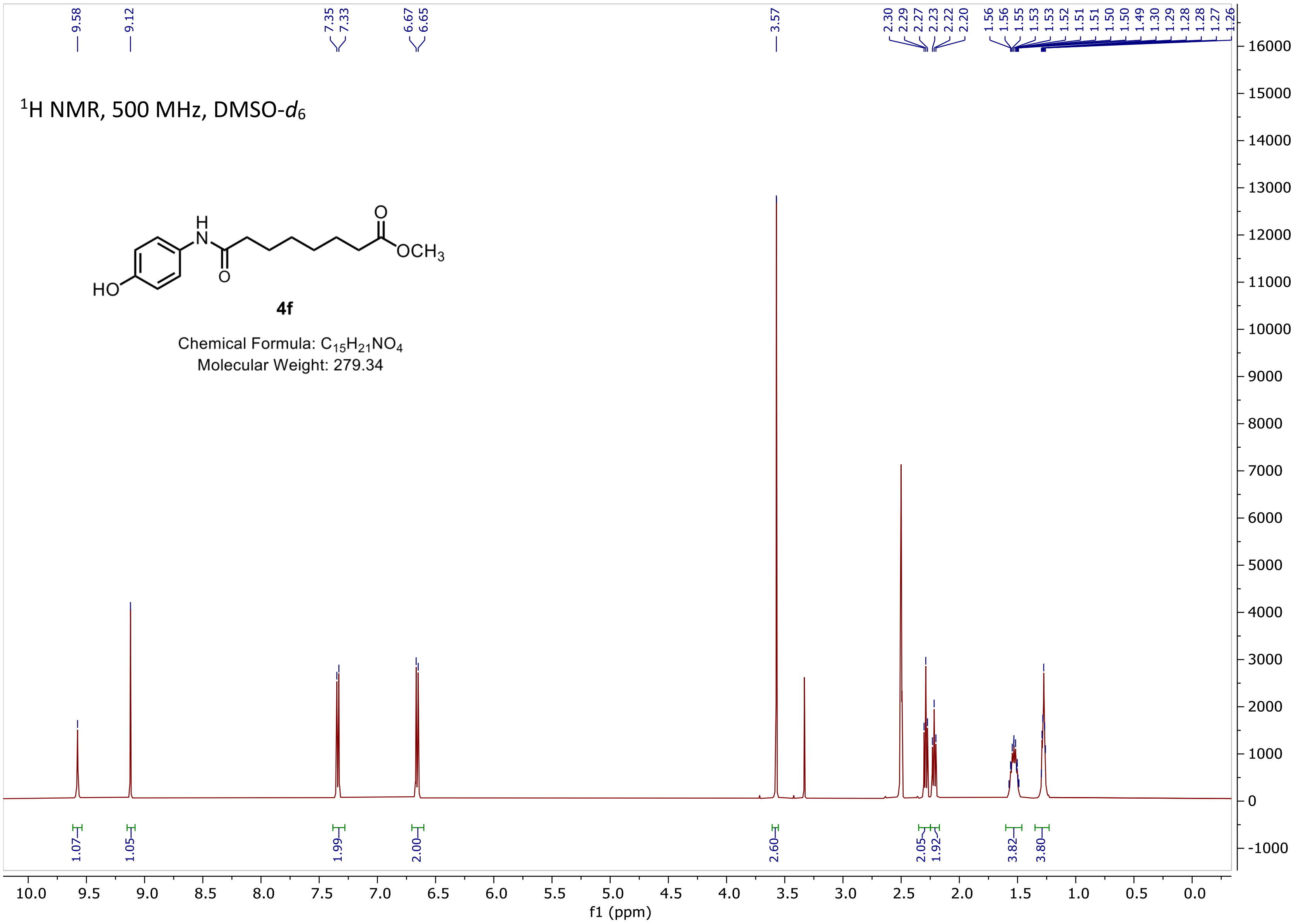

1H NMR, 500 MHz, DMSO-d6
HO
H
N
O
O
OCH3
4f
Chemical Formula: C15H21NO4
Molecular Weight: 279.34
f1 (ppm)

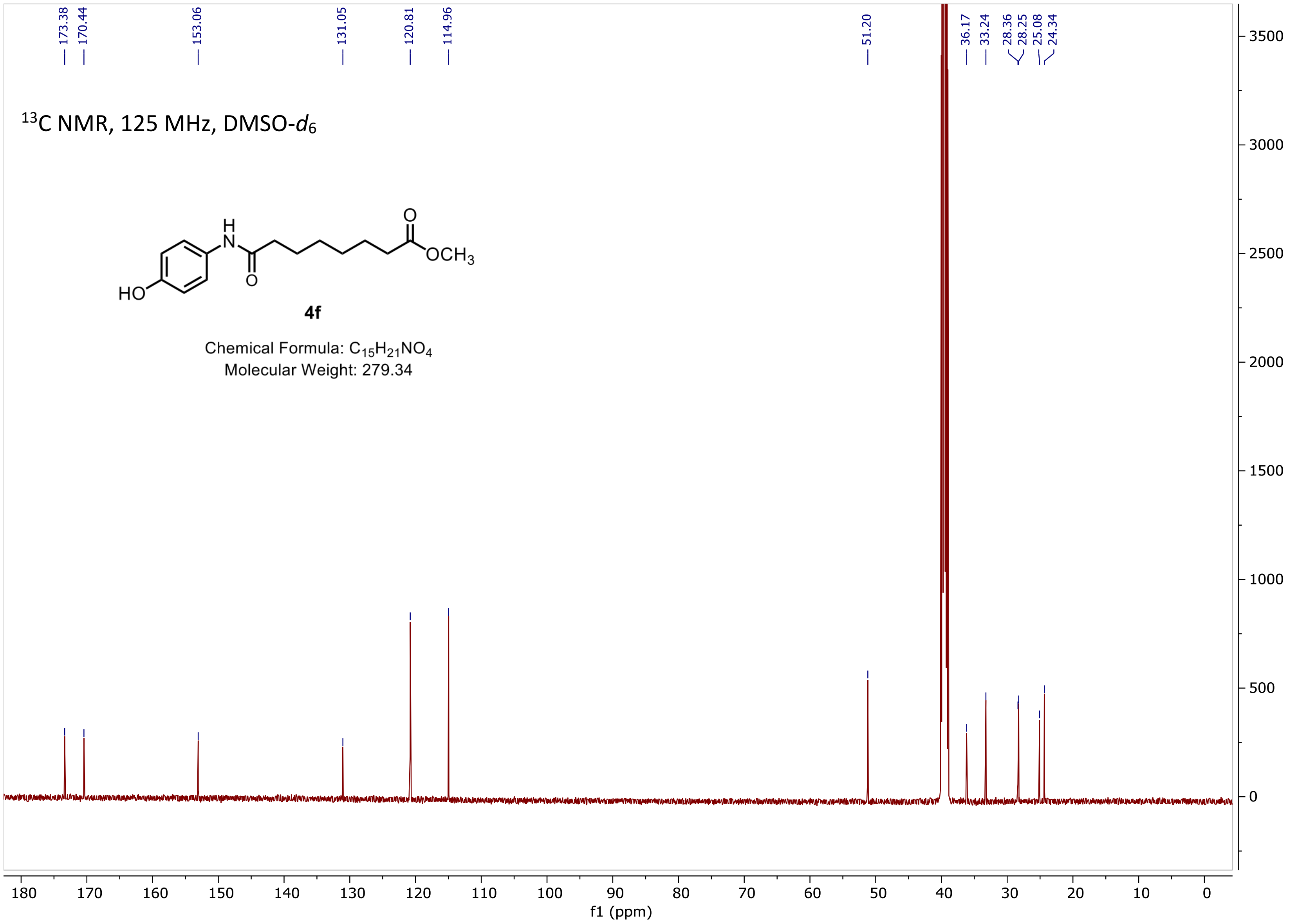

173.38
170.44
153.06
131.05
120.81
114.96
51.20
36.17
33.24
28.36
28.25
25.08
24.34
13C NMR, 125 MHz, DMSO-d6
H
N
O
O
OCH3
HO
4f
Chemical Formula: C15H21NO4
Molecular Weight: 279.34
3500
3000
2500
2000
1500
1000
500
0
180
170
160
150
140
130
120
110
100
90
80
70
60
50
40
30
20
10
0
f1 (ppm)

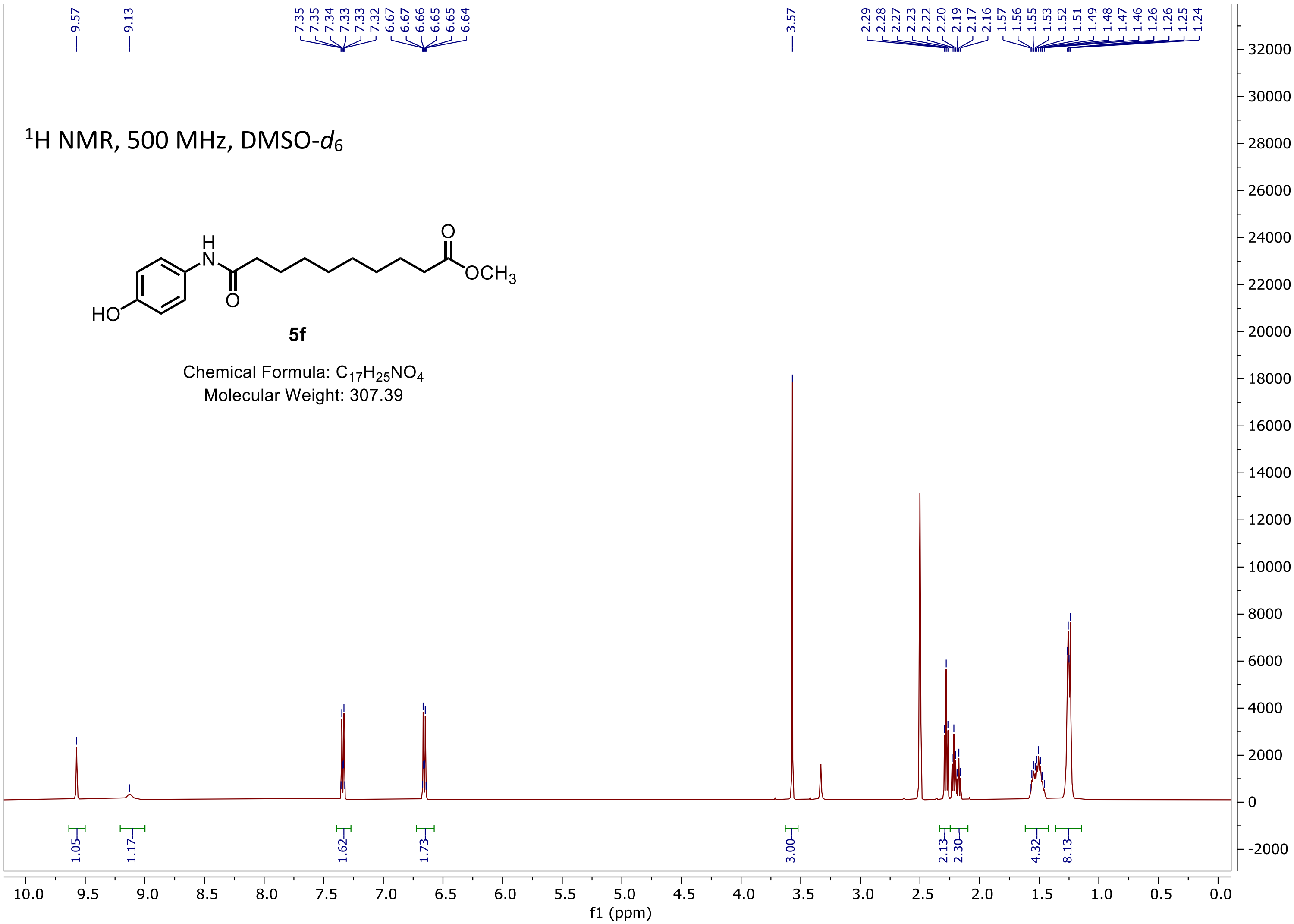

^{1}H NMR, 500 MHz, DMSO-d_6
5f
Chemical Formula: $C_{17}H_{25}NO_4$
Molecular Weight: 307.39
f1 (ppm)

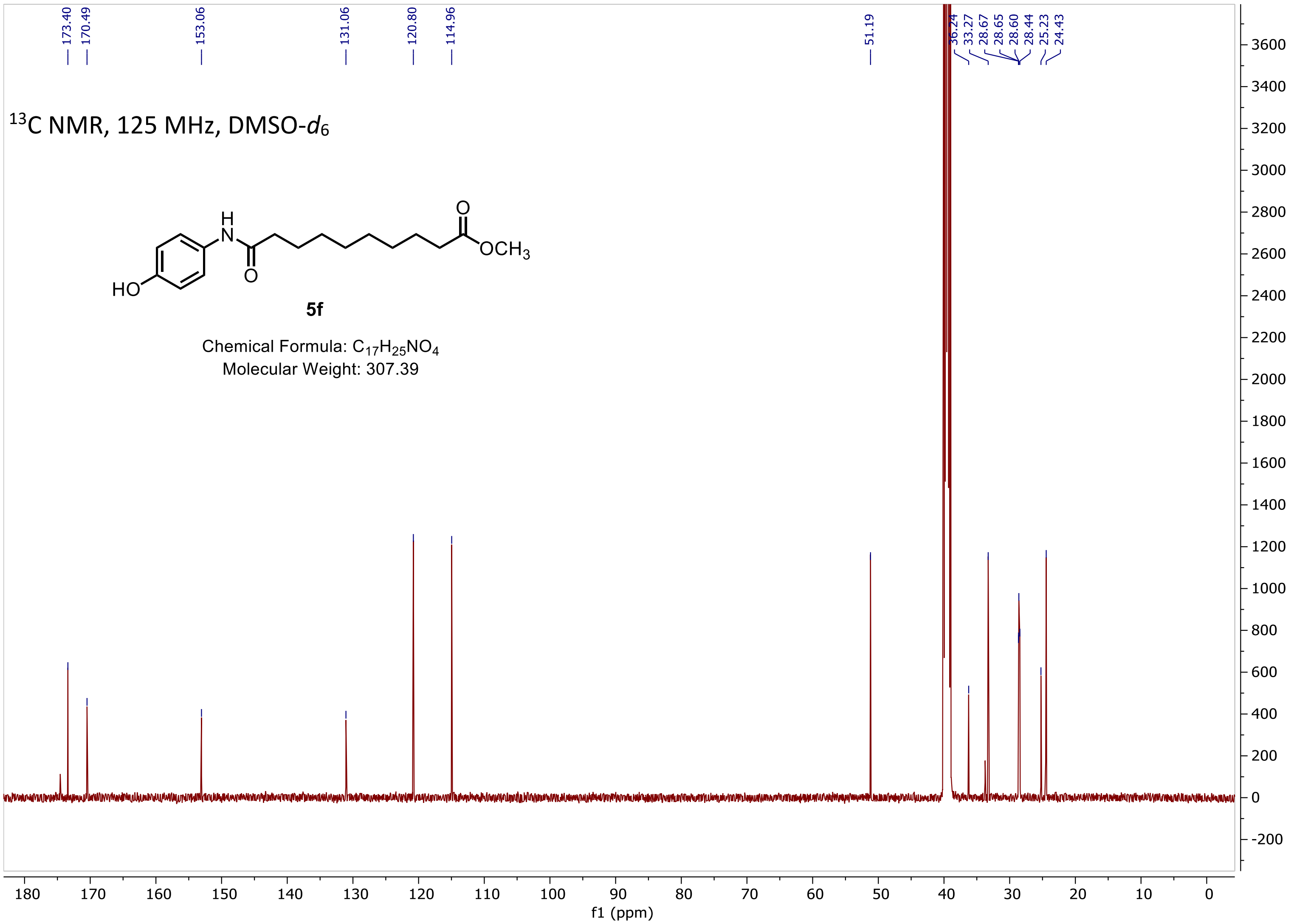

173.40
170.49
153.06
131.06
120.80
114.96
51.19
36.24
33.27
28.67
28.65
28.60
28.44
25.23
24.43
13C NMR, 125 MHz, DMSO-d6
H
N
O
O
OCH3
HO
5f
Chemical Formula: C17H25NO4
Molecular Weight: 307.39
f1 (ppm)

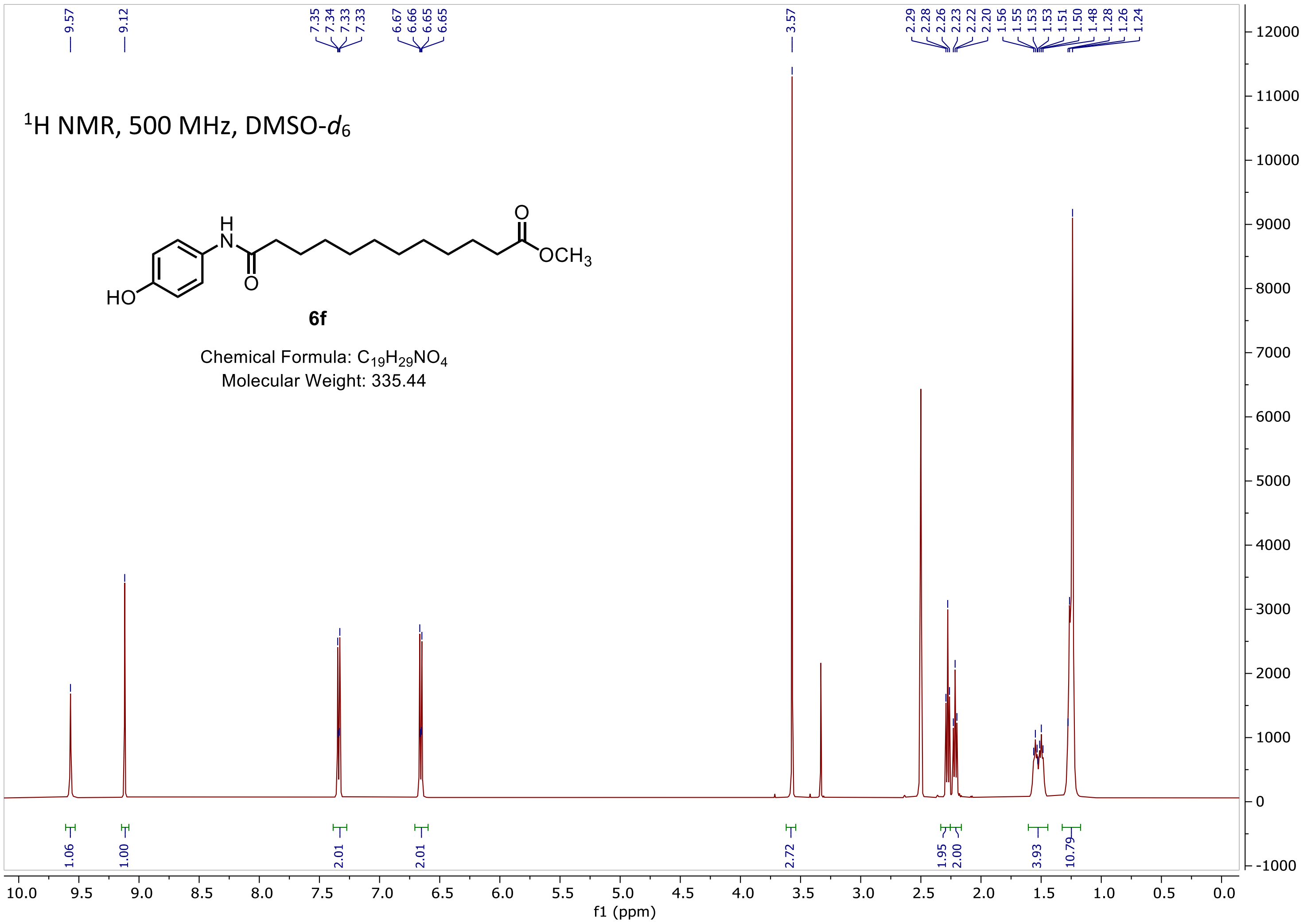

1H NMR, 500 MHz, DMSO-d6
6f
Chemical Formula: C19H29NO4
Molecular Weight: 335.44
f1 (ppm)

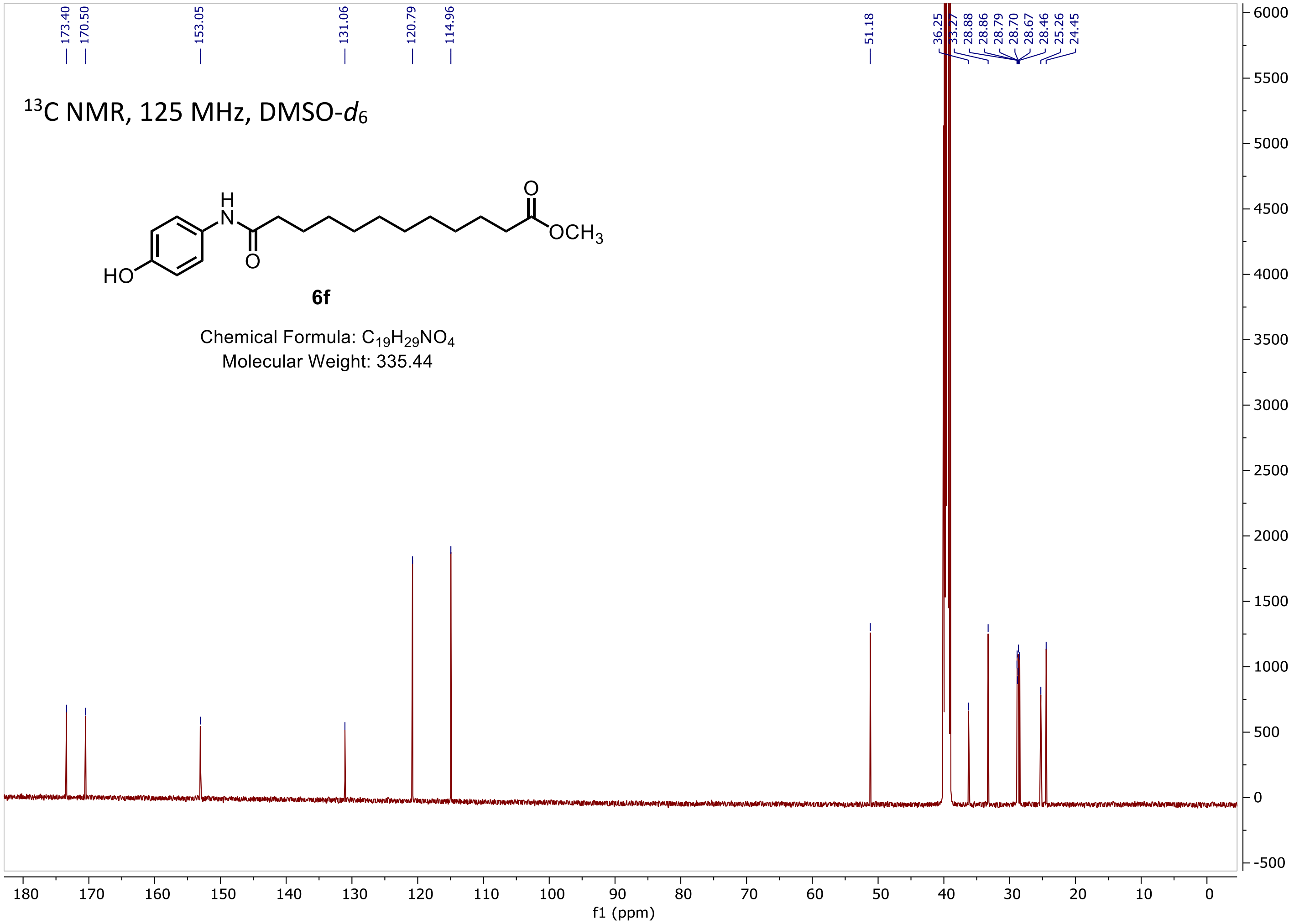

^{13}C NMR, 125 MHz, DMSO-d_6
HO
H
N
O
O
OCH3
6f
Chemical Formula: C19H29NO4
Molecular Weight: 335.44
173.40
170.50
153.05
131.06
120.79
114.96
51.18
36.25
33.27
28.88
28.86
28.79
28.70
28.67
28.46
25.26
24.45
f1 (ppm)

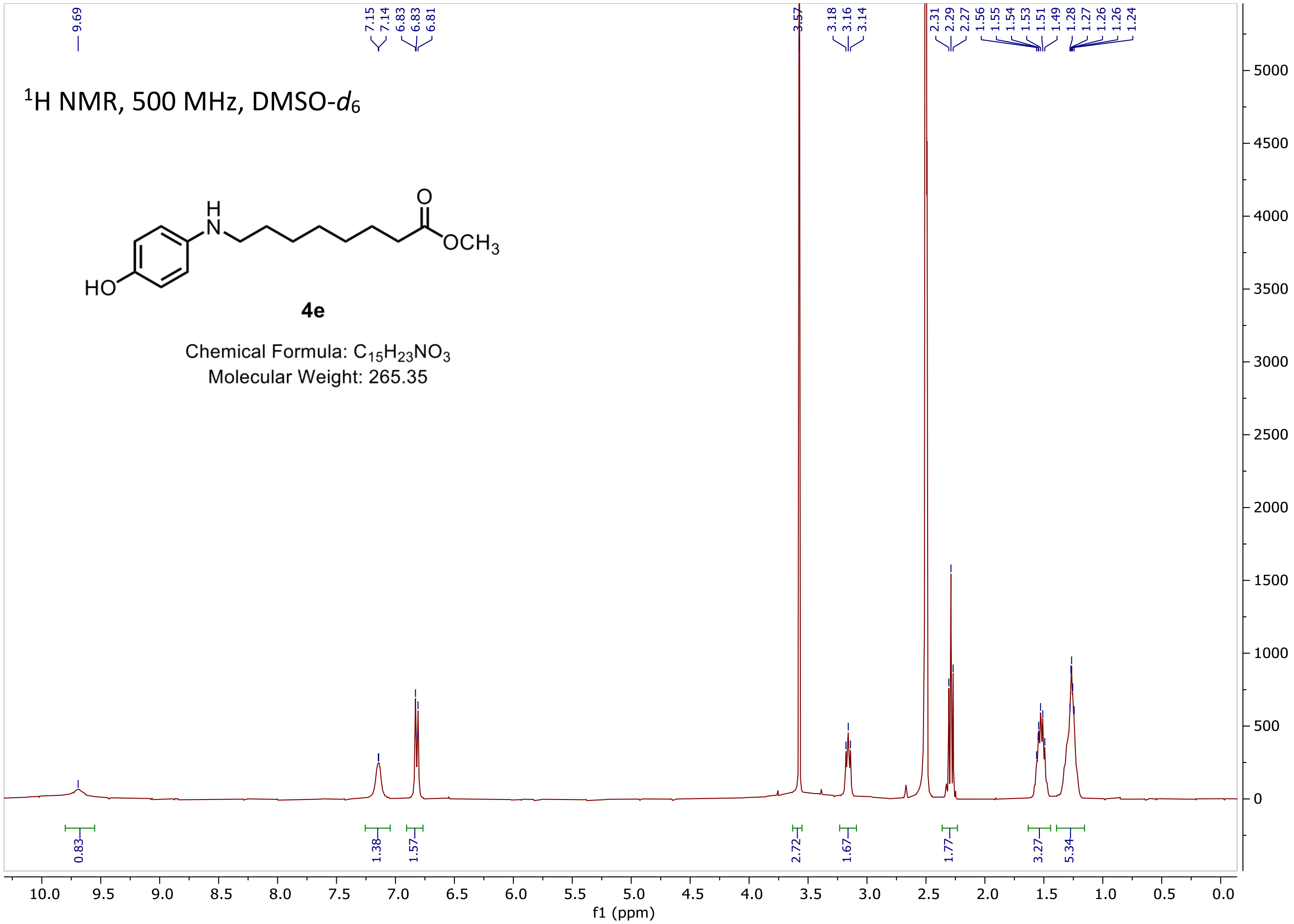

1H NMR, 500 MHz, DMSO-d6
4e
Chemical Formula: C15H23NO3
Molecular Weight: 265.35
f1 (ppm)

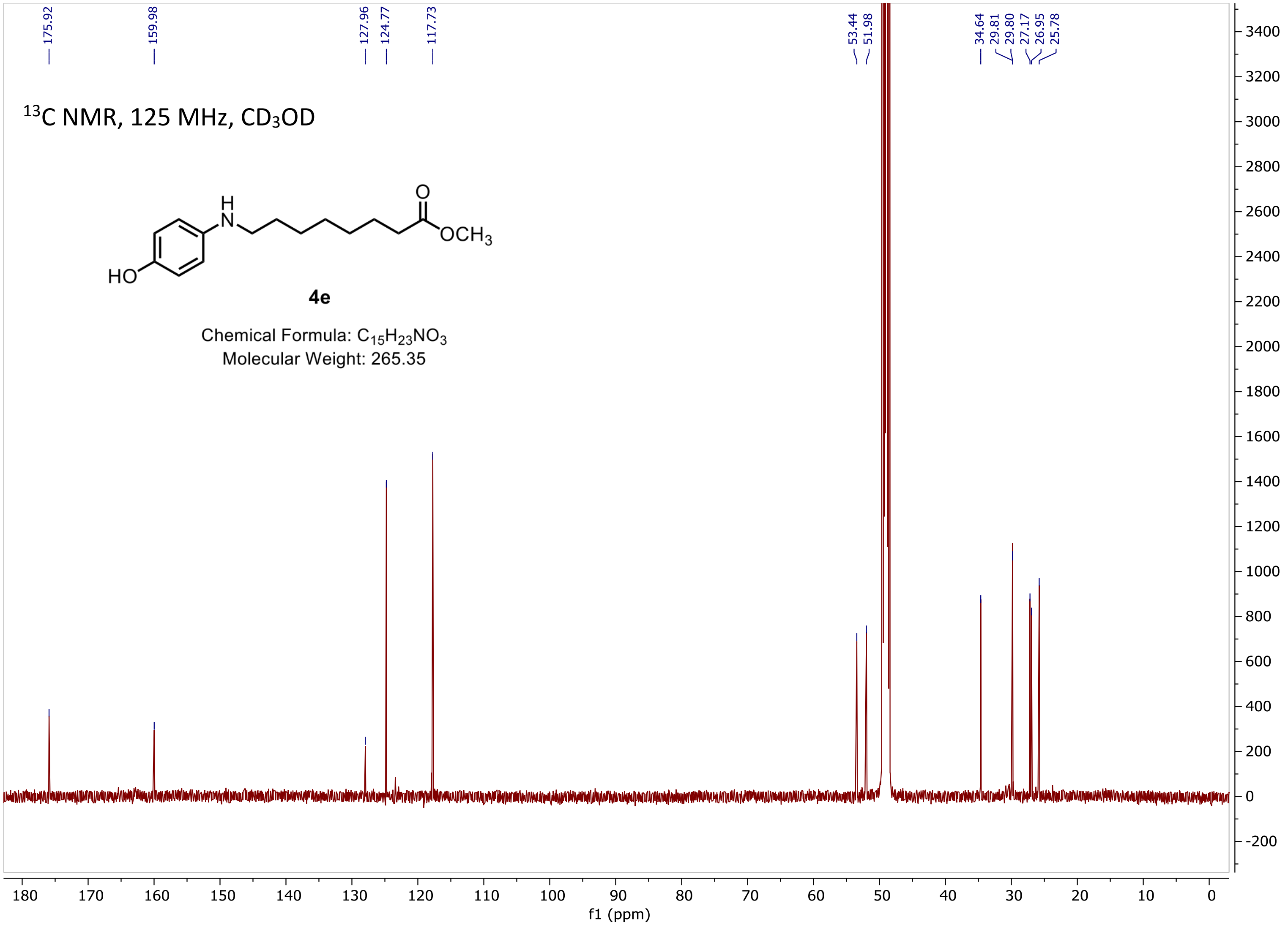

175.92
159.98
127.96
124.77
117.73
53.44
51.98
34.64
29.81
29.80
27.17
26.95
25.78
13C NMR, 125 MHz, CD3OD
HO
H
N
O
OCH3
4e
Chemical Formula: C15H23NO3
Molecular Weight: 265.35
3400
3200
3000
2800
2600
2400
2200
2000
1800
1600
1400
1200
1000
800
600
400
200
0
-200
180 170 160 150 140 130 120 110 100 90 80 70 60 50 40 30 20 10 0
f1 (ppm)

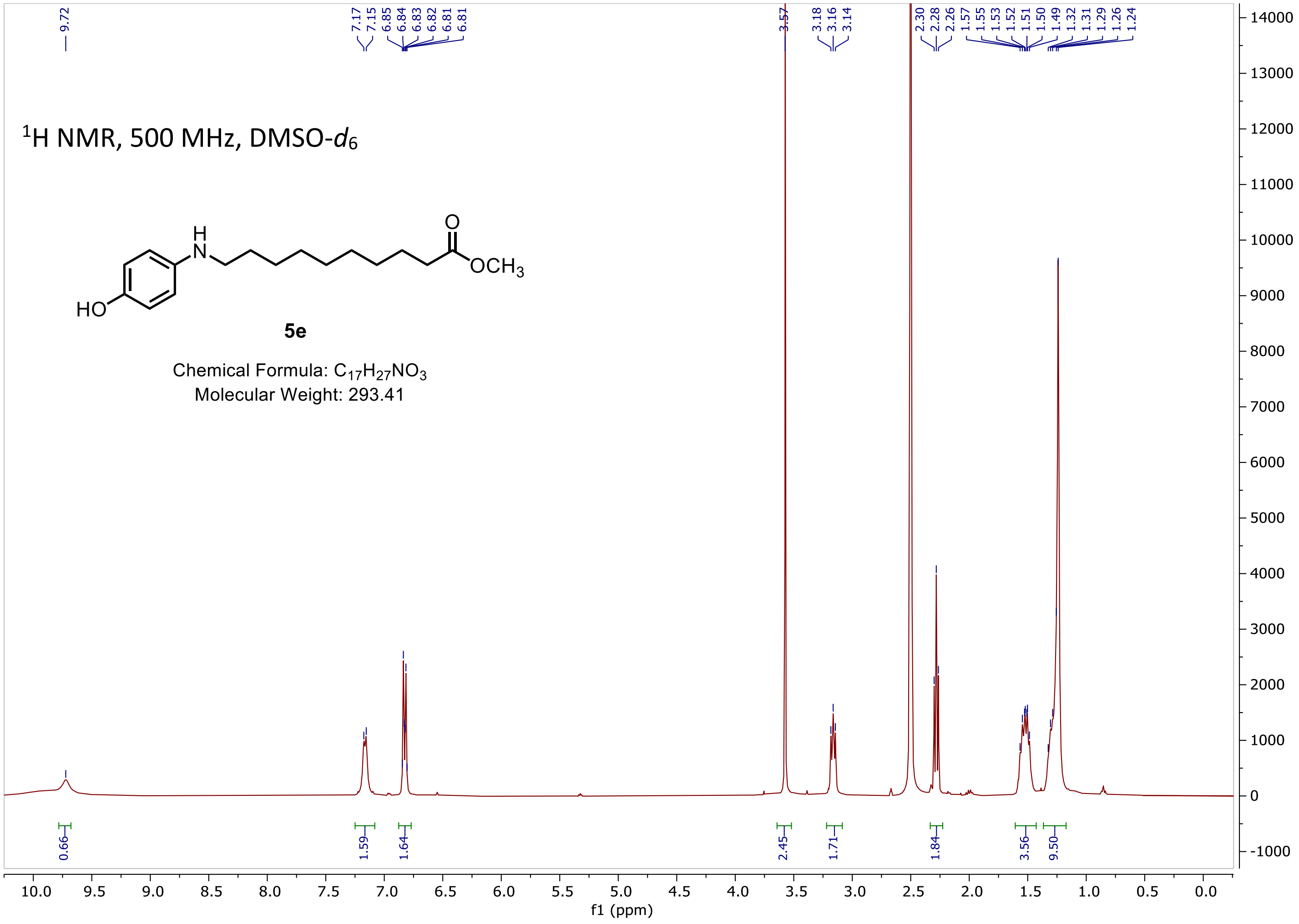

^{1}H NMR, 500 MHz, DMSO-d_6
HO
H
N
O
OCH$_3$
5e
Chemical Formula: $C_{17}H_{27}NO_3$
Molecular Weight: 293.41
9.72
7.17
7.15
6.85
6.84
6.83
6.82
6.81
6.81
3.57
3.18
3.16
3.14
2.30
2.28
2.26
1.57
1.55
1.53
1.52
1.51
1.50
1.49
1.32
1.31
1.29
1.26
1.24
0.66
1.59
1.64
2.45
1.71
1.84
3.56
9.50
f1 (ppm)

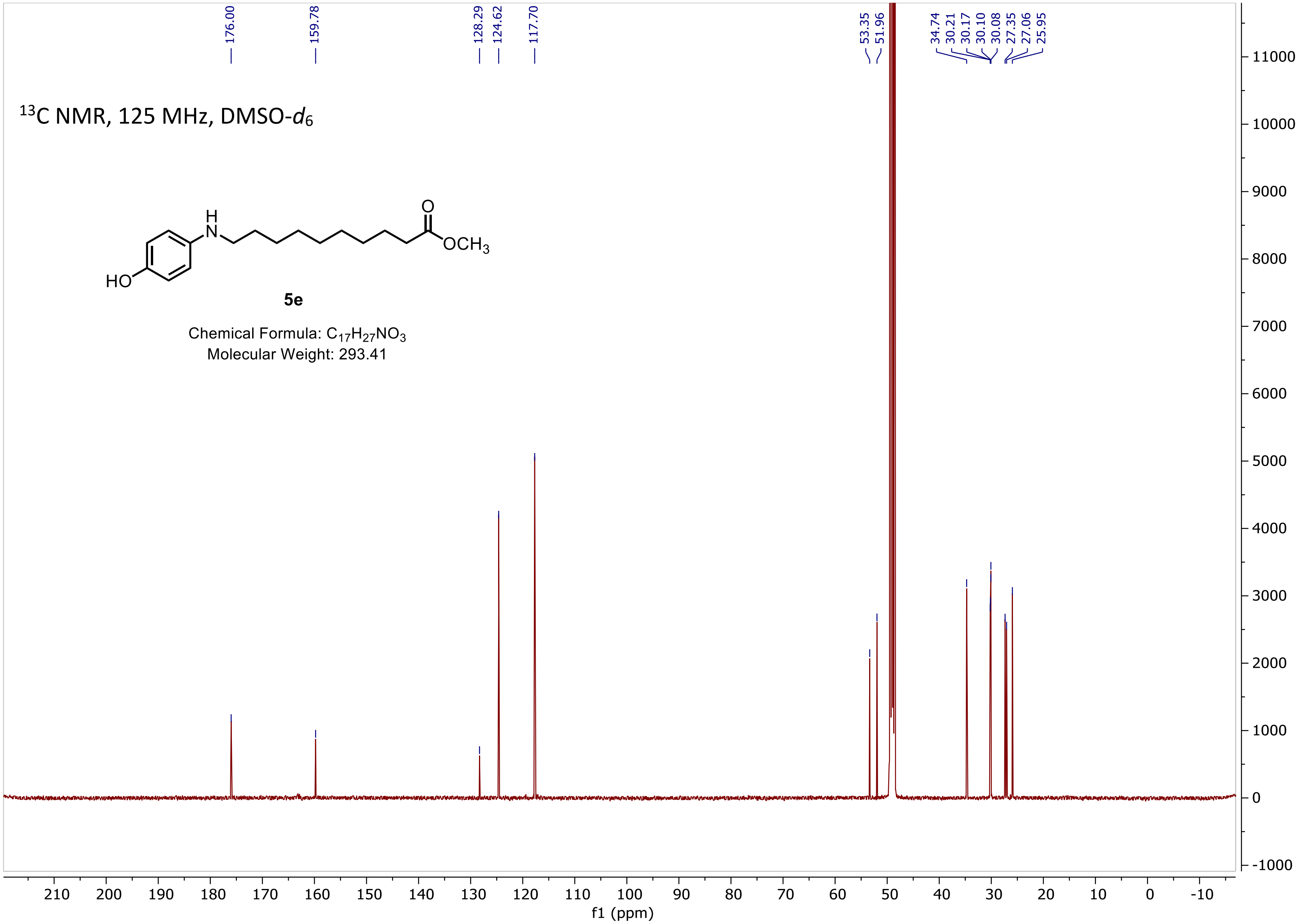

^{13}C NMR, 125 MHz, DMSO-d_6
HO
H
N
O
OCH_3
5e
Chemical Formula: $C_{17}H_{27}NO_3$
Molecular Weight: 293.41
176.00
159.78
128.29
124.62
117.70
53.35
51.96
34.74
30.21
30.17
30.10
30.08
27.35
27.06
25.95
f1 (ppm)

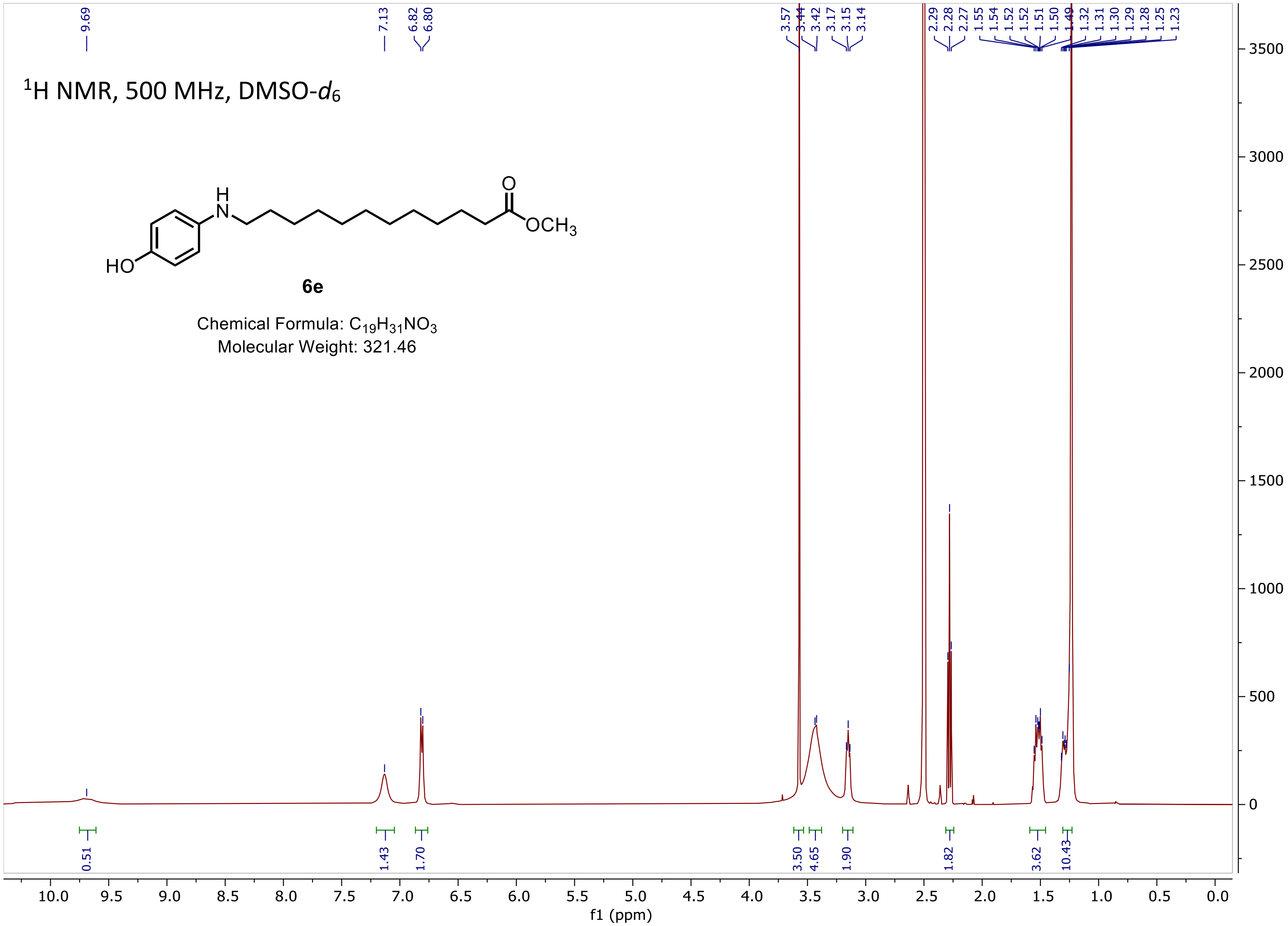

^{1}H NMR, 500 MHz, DMSO-d_6
H
N
O
OCH3
HO
6e
Chemical Formula: $C_{19}H_{31}NO_3$
Molecular Weight: 321.46
9.69
7.13
6.82
6.80
3.57
3.44
3.42
3.17
3.15
3.14
2.29
2.28
2.27
1.55
1.54
1.52
1.52
1.51
1.50
1.49
1.32
1.31
1.30
1.29
1.28
1.25
1.23
0.51
1.43
1.70
3.50
4.65
1.90
1.82
3.62
10.43
10.0
9.5
9.0
8.5
8.0
7.5
7.0
6.5
6.0
5.5
5.0
4.5
4.0
3.5
3.0
2.5
2.0
1.5
1.0
0.5
0.0
f1 (ppm)
3500
3000
2500
2000
1500
1000
500
0

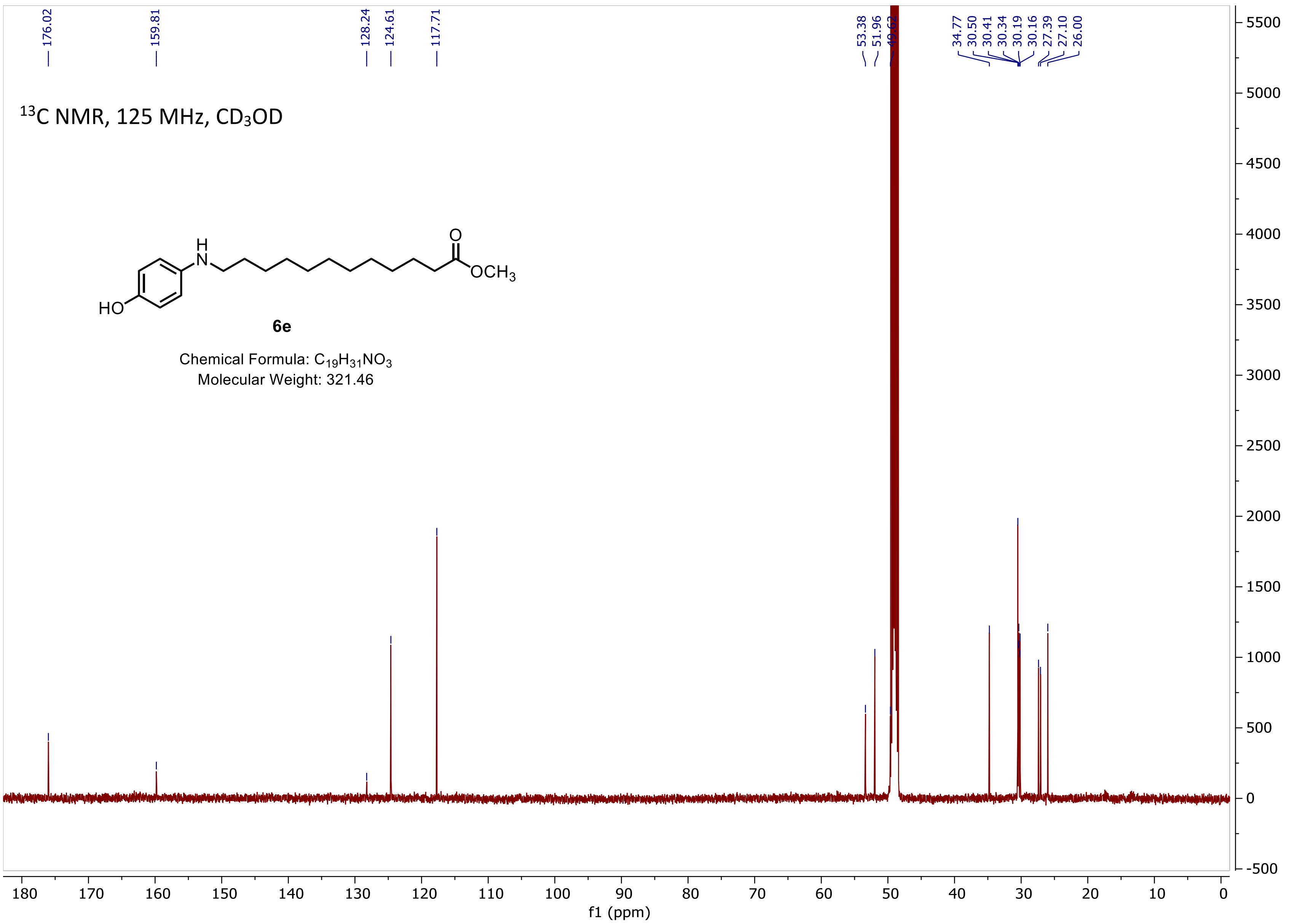
176.02
159.81
128.24
124.61
117.71
53.38
51.96
34.77
30.50
30.41
30.34
30.19
30.16
27.39
27.10
26.00
13C NMR, 125 MHz, CD3OD
H
N
O
OCH3
HO
6e
Chemical Formula: C19H31NO3
Molecular Weight: 321.46
f1 (ppm)
